# Human amyotrophic lateral sclerosis/motor neuron disease: The disease‐associated microglial pathway is upregulated while *APOE* genotype governs risk and survival

**DOI:** 10.1111/bpa.70019

**Published:** 2025-06-12

**Authors:** Bridget A. Ashford, Julie E. Simpson, Charlotte Dawson, Delphine Boche, Johnathan Cooper‐Knock, Paul R. Heath, Daniel Fillingham, Charlie Appleby‐Mallinder, Wenbin Wei, Mark Dunning, J. Robin Highley

**Affiliations:** ^1^ SITraN University of Sheffield Sheffield UK; ^2^ Neuroscience Institute University of Sheffield Sheffield UK; ^3^ Clinical Neurosciences, Clinical and Experimental Sciences, Faculty of Medicine University of Southampton Southampton UK; ^4^ Advanced Manufacturing Research Centre University of Sheffield Rotherham UK; ^5^ Department of Biosciences University of Durham Durham UK; ^6^ Bioinformatics Core, School of Medicine and Population Health University of Sheffield Sheffield UK

**Keywords:** amyotrophic lateral sclerosis, APOE, disease‐associated microglia, inflammation, motor neuron disease

## Abstract

A key role for inflammation in amyotrophic lateral sclerosis/motor neuron disease (ALS/MND) has been identified. It is vital to assess which central nervous system structures are most affected and which inflammatory processes are responsible in humans. The inflammatory transcriptome was characterized in the cervical spinal cord and motor cortex in post‐mortem frozen and formalin‐fixed paraffin‐embedded specimens from human sporadic ALS/MND and control cases using the nCounter® Neuroinflammation Panel. Archival data were reanalyzed and compared with the nCounter data. Immunohistochemistry was used to examine the inflammatory response in the spinal cord and motor cortex and validate changes found during transcriptomic analyses. In the spinal cord, marked inflammation was observed, while less inflammation was detected in the motor cortex. Examination of differentially expressed genes in the spinal cord highlighted *TREM2*, *TYROBP*, *APOE*, and *CD163*, as well as phagocytic pathways. In sporadic ALS/MND spinal cord, significant microglial reactivity and involvement of TREM2, ApoE (encoded by *APOE*), and TYROBP were confirmed, suggesting the involvement of the disease‐associated microglial (DAM) phenotype. The corticospinal tracts showed greater inflammation than the ventral horns. The precentral gyrus of ALS/MND again showed less immune reactivity to disease when compared to controls. Finally, in the largest cohort assessed to date, we demonstrate an association between the *APOE* variant and ALS/MND risk, age of onset, and survival. We find confirmed associations between *APOE* ε3/ε3 and disease and between ε2/ε2 and absence of disease. Further, ε4/ε4 appears to be associated with earlier disease onset and a more aggressive course. We conclude that while there is widespread inflammation in the CNS in sporadic ALS/MND, this is more marked in the spinal cord, especially the corticospinal tract. The specific markers stress the DAM phenotype as having a key role together with a possible influx of somatic macrophages. In addition, *APOE* function and genotype may be relevant in ALS/MND.

## Graphical abstract

ALS/MND‐related inflammation is investigated in the motor cortex and spinal cord by both transcriptomics and immunohistochemistry. This reveals greater inflammation in the spinal cord and highlights the disease‐associated microglial (DAM) phenotype characterized by ApoE, TREM2, and TYROBP. Genetic analyses reveal that APOE variants are related to the risk of ALS/MND and severity.

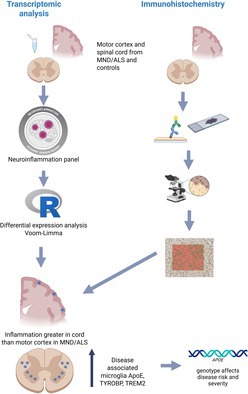

## 1 INTRODUCTION

Motor Neuron Disease (MND) is a fatal neurodegenerative condition, characterized by the progressive degeneration of motor neurons [1] with an incidence of 1.5–2 diagnoses in 100,000 people per year [2]. Amyotrophic lateral sclerosis (ALS) is the most common clinical manifestation of MND in adults (accounting for 80%–90% of cases) and as such, the terms ALS and MND are often used interchangeably. The majority (90%–95%) of cases are sporadic. Survival time varies considerably, although 80% of patients survive only 2–5 years after diagnosis [3]. Pathologically, ALS/MND is characterized by motor neuron and pyramidal tract degeneration, together with various proteinaceous inclusions composed principally of TDP‐43 (MND‐TDP) or cystatin C.

Microglia are the resident immune cells of the central nervous system (CNS), and account for between 5% and 12% of cells within the brain. Microglial density and function vary greatly with CNS region and age [4, 5]. Under physiological conditions, microglia have a ramified morphology with small soma and fine processes [6]. Under pathological conditions, the cells react to pathogens and damaged endogenous cells via Damage‐Associated Molecular Patterns (DAMPs) with changes both in biological activity (with associated molecular markers) and morphology [7]. This results in swollen and shortened cytoplasmic processes (hyper‐ramification) and ultimately adopts a large, “amoeboid” morphology. Microglia present multifaceted signaling responses, leading to a complex phenotype. This changes with several factors, including the region of the CNS [8], age [9], activating stimuli, and disease [10].

The microglial response is critical for CNS protection through the destruction and removal of damaged or dysfunctional cells or pathogens and the provision of trophic support. This response is highly effective. However, where the initial trigger is not resolved, a destructive cycle of microglial activation and neuron death is initiated: DAMPs that are released by degenerate cells can cause further inflammatory microglial activation, resulting in neuronal death [11]. This can drive the progression of neurodegeneration [12].

There is good evidence that neuroinflammation is a key feature of human ALS/MND. Firstly, transcriptomic analyses of *post‐mortem* spinal cord have highlighted inflammation as one of the most altered pathways [13]. At sites of neuronal loss in *post‐mortem* tissues, CD68 and Iba1 immunohistochemistry (IHC) reveal microglia to transition from their ramified morphology to an “activated” morphology [14, 15, 16]. Microglial activation correlates with the extent of TDP‐43 pathology, executive dysfunction, upper motor neuron symptoms, and the rate of disease progression [15, 17]. Secondly, in vivo positron emission tomography (PET) shows increased signal in both motor and extra‐motor regions [18], and an association between microglial activation and cortical thinning and a worse disease phenotype [17]. Finally, CSF from ALS/MND patients has high levels of inflammatory cytokines [19, 20, 21], the expression of which has been correlated with the disease progression rate [21, 22, 23].

Human studies have established the presence of a generalized microglial reaction in motor neuron disease/amyotrophic lateral sclerosis. Attempts to specify which of the many molecular behaviors of microglia are relevant have predominantly utilized transgenic animal models of familial (not sporadic) ALS/MND. The most characterized and widely used models are those overexpressing *SOD1* mutations [24, 25, 26, 27]. More recently, models have been created that contain transgenes with mutations in other genes (summarized by Lutz, 2018 [28]), as well as in other species, including rats [29] and zebrafish [30, 31, 32]. Such studies have highlighted both toxic and neuroprotective microglial functions. However, there have been considerable inconsistencies. For example, older studies indicated a tendency toward an early increase in trophic and anti‐inflammatory gene expression prior to symptom onset, followed by a switch to a more toxic, proinflammatory phenotype. In contrast, more recent studies have provided evidence indicating microglia express both protective and toxic factors consistently throughout disease (see [13]).

Given the conflicting data from animal studies, it is worthwhile attempting to profile the inflammatory responses using human tissue. There are other good reasons to study MND/ALS‐related neuroinflammation in human tissue. Thus, many animal studies have focused on pathology related to *SOD1* mutations. However, in human MND/ALS, patients with *SOD1* mutations (MND‐SOD1) have a different pathological profile, lacking TDP‐43 proteinopathy and having less extramotor disease, and have a subtly different clinical profile compared to more common forms of disease. It is thus arguably not comparable with “classical” ALS/MND pathology where there is TDP43 proteinopathy (MND‐TDP). This issue, in combination with significant interspecies differences in anatomy and immunity [9] raises questions about the validity of animal models to study inflammation in MND. Finally, transferring microglia from native tissue to in vitro culture has been shown to alter their transcriptome [33]. This artefactual gene expression shift particularly impacts genes relevant to studies of neurodegeneration [34].

For this reason, it is crucial to supplement the study of microglia in model systems with studies conducted in humans. The study that we report characterizes the neuroinflammatory and microglial phenotype in the motor system in human sporadic MND.

We performed a transcriptomic analysis of the motor regions of the CNS (ventral horn of the spinal cord and precentral gyrus—motor cortex) from control and sporadic ALS/MND cases to identify a neuroinflammatory signature. We then proceeded to use immunohistochemistry for microglial and macrophage markers to assess inflammation in the motor system of sporadic MND. Finally, we accessed publicly available data from the project MinE database [35] and demonstrated a very significant relationship between APOE haplotype and MND, which affects both disease risk and severity.

## 2 METHODS

### 2.1 Tissue collection, preparation, and RNA extraction for nCounter analysis

*Post‐mortem* snap‐frozen and formalin‐fixed paraffin‐embedded (FFPE) cervical spinal cord tissue and snap‐frozen motor cortex were obtained from the Sheffield Brain and Tissue Bank. Each cohort consisted of 16 sporadic ALS/MND cases of varying survival times from diagnosis to death and 8 normal control cases (Tables S1–S3).

Frozen tissue was brought up to −20°C from −80°C for dissection in a freezer cabinet where the ventral horns and motor cortex were isolated from surrounding tissue by a qualified neuropathologist (JRH). Tissue was sampled unstained from the cervical enlargement. The tissue was visualized wearing a headband‐mounted, illuminated magnification visor with 3× magnification. This allowed good visualization of the gray/white matter interface, even without staining. A cross‐section slice of cord approximately 1 mm thick was first cut freehand with a razor blade. The ventral horns were dissected from this using a scalpel. All dissections were performed freehand. Approximately 20–40 mg of tissue was collected per sample. For the formalin‐fixed, paraffin‐embedded (FFPE) spinal cord tissue, 10 sections of 50 μM thickness were cut, and the ventral horns were isolated as for the frozen spinal cord in a similar manner.

RNA was extracted from frozen tissue using the Zymo Research Direct‐zol™ RNA Miniprep kit (Zymo Research, R2050) and from FFPE tissue using the Qiagen® RNeasy FFPE kit (Qiagen, 73504) following manufacturers' instructions.

### 2.2 nCounter gene expression assay

The Bruker Spatial Biology (previously NanoString) Sprint Profiler Gene expression assay using the nCounter® Human Neuroinflammation Panel (XT‐CSO‐HNROI1‐12) was used on samples of 100 ng of RNA (5 μL at 20 ng/μL on the advice of nCounter technical staff) according to the manufacturer's protocols. The nCounter data are available at the University of Sheffield Online Research Data facility (https://hdl.handle.net/10779/sheffield.27908097.v1).

The nCounter data were analyzed using the limma‐voom method [36]. Briefly, data was normalized using the Trimmed Mean of M Values method and transformed using the voom function. A linear model for each gene was fitted using the lmFit function. The effect of age and sex was controlled for by adding these as covariates in the analysis. Tests for significance were performed using the eBayes function. Differentially expressed genes were identified with the criteria of Benjamini‐Hochberg adjusted *p*‐value (false discovery rate) <0.05 and absolute fold change >|1.5|.

For the frozen tissue, all cases were kept for analysis. FFPE spinal cord data for three cases (two controls and one MND) were discarded due to low count number.

The nCounter Neuroinflammation panel annotates each gene with associated KEGG pathways. The KEGG pathways from all significantly differentially expressed genes were counted to identify those that appeared most frequently. Pathway analysis was not performed on the motor cortex as insufficient genes reached significance following false discovery rate correction.

### 2.3 Length of survival analyzes

The association of genes with the length of survival from onset to death in sporadic ALS/MND cases was assessed using Cox proportional hazards regression and the log‐rank test in the R survival package (https://cran.r‐project.org/web/packages/survival/index.html). Cases were split into high‐ or low‐expressing groups for each gene based on the median expression across cases. The effect of age and sex on survival was also analyzed using Cox proportional hazards regression. Age and sex did not have a significant influence on survival in this dataset.

### 2.4 Microglial/macrophage deconvolution

Those genes most associated with microglial/macrophage expression were determined using the online Brain RNASeq tool (https://www.brainrnaseq.org/; [37]). Each of the differentially expressed genes was entered into the database, and the fragments per kilobase of transcript per million mapped reads for the main glial/CNS cell types were given. A gene was determined to be microglial/macrophage specific if expression was greatest in the microglial/macrophage population and not highly expressed by any other cell population.

### 2.5 Bulk RNA‐seq analysis

To further assess the robustness of the nCounter dataset, we compared our results to the RNAseq data from another laboratory (NCBI sequence read archive SRP064478, [38]). This cohort consisted of six sporadic ALS/MND cases (three male and three female) with a median age of 68.5 years and eight control cases (four male, four female) with a median age of 66.5 years. One case (ALS4, male) was excluded as this case has a pathogenic *SOD1* mutation.

The publicly available RNAseq reads (fastq files) from this study were quantified and aligned using the Salmon method. The resulting quant files were then imported into R for further analysis. Genes expressed over background in less than two cases were classed as low‐expressing and were filtered out. Box plots were used to visualize the spread of the raw data and normalized data, which showed similar distribution across all samples. Heat maps were plotted, and PCA was performed to look for outliers. However, no clear outliers were identified; therefore, all cases were kept. Differential expression was performed using the generalized linear model framework of DESeq2 with the control group as the baseline [39].

### 2.6 Immunohistochemistry for neuroinflammatory markers in MND/ALS

FFPE *post‐mortem* tissue from the precentral gyrus and cervical spinal cord was obtained from the Sheffield Brain and Tissue Bank for immunohistochemistry. Tissue sections were used for assessment of the spinal cord (*n* = 28 sporadic ALS/MND, *n* = 14 control; Table S4). These were largely different cases from those that had been used for nCounter assessment: Only three ALS/MND cases and two controls had been assessed by nCounter on frozen tissue. The motor cortex and underlying white matter were assessed using tissue microarrays (*n* = 63 sporadic ALS/MND, *n* = 7 control; Table S5), with two cores sampled from each tissue using the Beecher MTA‐1 Tissue Microarrayer [40]. Again, these were largely different cases from those that had been used for nCounter assessment: Only five ALS/MND cases and no controls had been assessed by nCounter on frozen tissue.

Immunohistochemistry was performed using the avidin‐biotin complex (ABC) method on 5 μm‐thick sections using antibodies, antibody concentrations, and antigen retrieval protocols detailed in Table S6. Slides were scanned using the Hamamatsu NanoZoomer slide scanner at ×40 magnification (Hamamatsu Photonics, Japan).

TREM2 immunohistochemistry was performed in bone marrow and spleen in order to explore specificity (Figure S1).

### 2.7 Analysis of co‐expression

ApoE expression in the CNS is known to be primarily astrocytic [41]. However, as TREM2 and TYROBP are less well characterized, we performed double staining to determine which cell type was expressing these markers. Slides were dewaxed, rehydrated through alcohols, and endogenous peroxidases were blocked in 3% hydrogen peroxide for 20 mins. The slides underwent antigen retrieval and a standard ABC immunohistochemistry protocol. The first protein of interest was visualized using the ImmPACT AMEC red substrate kit (Vector Laboratories, SK‐4285). Slides were washed in tap water and mounted using aqueous mounting media, dried, and digitized. Following this, slides were decoverslipped, and the dye was dissolved in graduated alcohols. Unbound avidin and biotin from the first stain were blocked using avidin and biotin blocking solution (Vector Laboratories, SP‐2001). The slides were then rehydrated and stained using a standard ABC protocol using a second primary antibody, visualized with DAB, coverslipped, and rescanned.

### 2.8 Image analysis

Visiopharm (Hoersholm, Denmark) image analysis software was used to quantify immunoreactivity using a previously developed “modular analysis unit” (referred to as an APP by Visiopharm), which assesses area density (percentage of a region of interest that is positive for immunoreactivity) [40]. Following pre‐processing, modular analysis units were trained specifically for each protein of interest, using the Visiopharm training wizard.

In spinal cord sections, the ventral horns, the dorsal column, and the lateral corticospinal tracts were selected for quantification by manually drawing around these structures on the whole microscope slide images within Visiopharm, using an adjacent Luxol fast blue‐stained section to delineate the appropriate anatomy where necessary.

In spinal cord sections stained for TREM2, motor neurons showed evidence of lipofuscin staining by DAB, which the Visiopharm software was unable to differentiate from true signal. To prevent bias in quantification—as sporadic ALS/MND cases will have fewer motor neurons compared to controls, neurons were excluded from analysis using a filter that reclassifies DAB‐positive areas larger than 200 μM as areas in the background (Figure S2).

In the spinal cord, specific regions were selected for quantification: the ventral horns, the lateral corticospinal tracts, and the dorsal column. The latter is an ascending sensory tract that we expected to be less affected by MND pathology.

All statistical analysis was performed using GraphPad Prism (versions 7,8,9). For all statistical analyzes, the significance value was set at alpha <0.05.

As many data sets were not normally distributed (by Shapiro‐Wilk), non‐parametric statistics were used for intergroup comparisons: The Kruskal‐Wallis test followed by post hoc Mann–Whitney *U* tests was used for most intergroup comparisons.

To examine the relationship with patient survival, sporadic ALS/MND cases were split into fast‐ or slow‐progressing groups based on the median length of survival for each cohort, again using Kruskal–Wallis followed by post hoc Mann–Whitney *U* tests.

### 2.9 Genotyping of APOE

Phased genotyping data for rs429358 and rs7412 were obtained from the latest ALS/MND GWAS consisting of 29,612 ALS/MND patients and 122,656 controls [35]. Samples were assigned to APOE haplotypes ε4, ε4/ε3, ε3/ε3, ε4/ε2, ε3/ε2, and ε2/ε2. Analysis of the relationship between ALS/MND status and APOE genotype was conducted by multivariable logistic regression using sex and the first 10 principal components of genetic variation as covariates. For this analysis, homozygous and heterozygote individuals were considered equivalent. Cox regression was used to assess the relationship with survival and age of disease onset in a subset of 4897 patients where phenotypic information was available; this analysis used sex, age (for survival only), and the first 10 principal components of genetic variation as covariates.

## 3 RESULTS

### 3.1 The transcriptomic profile shows considerable inflammation of the spinal cord in MND/ALS and highlights the ApoE‐TYROBP‐TREM2 pathway

**FIGURE 1:**
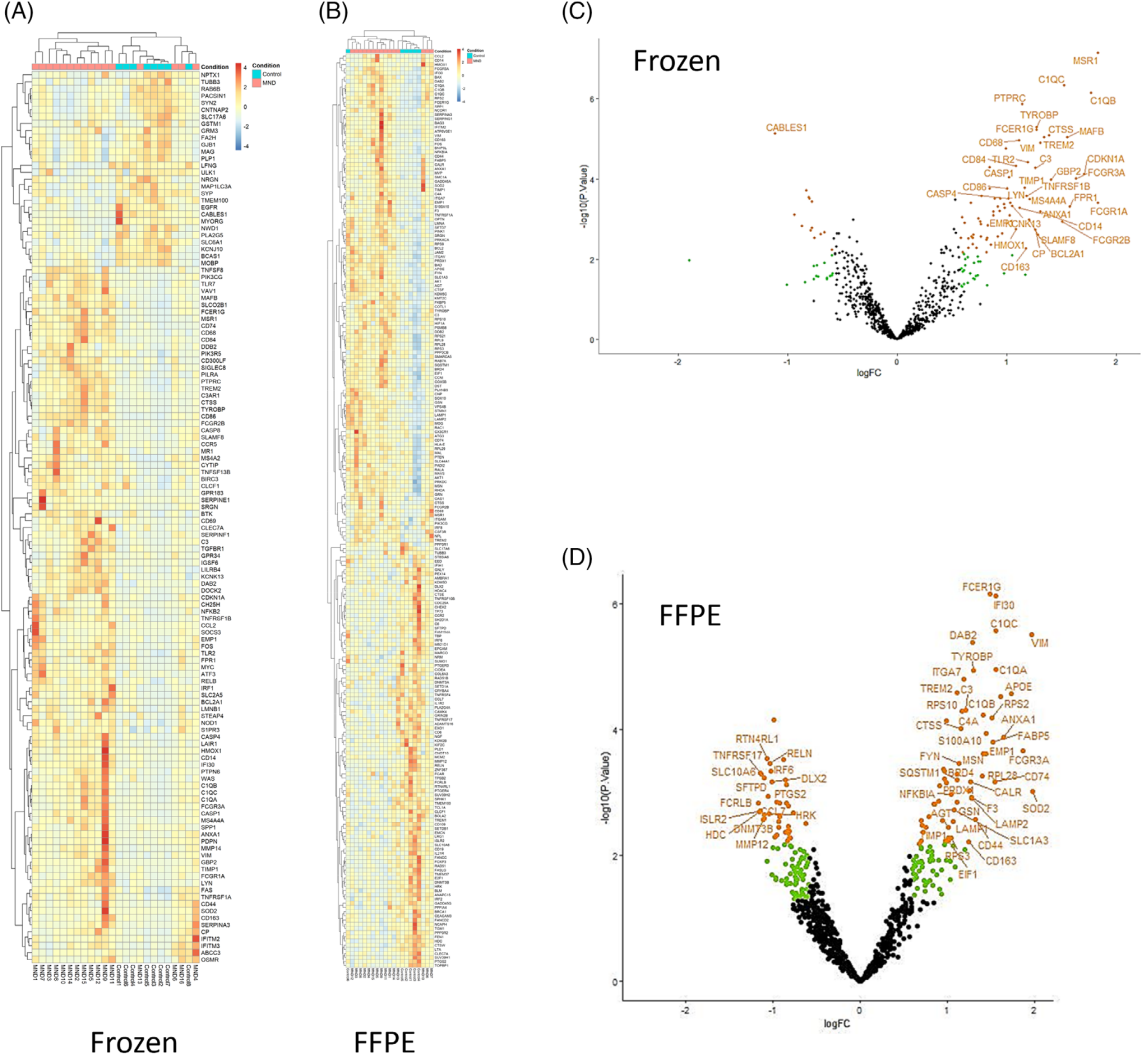
sMND is associated with an increase in inflammatory signaling in the spinal cord—frozen and FFPE tissue. (A) Heatmap displaying the normalized and row‐scaled expression of 128 differentially expressed genes (*p* < 0.05 and FC >1.5) between MND and neurologically healthy cases. Genes are shown on the y‐axis. (B) Heatmap displaying the normalized and row‐scaled expression of 219 differentially expressed genes (*p* < 0.05 and FC >1.5) between MND and neurologically healthy cases. Genes are shown on the y‐axis. (C) Volcano plot for frozen spinal cord tissue showing fold change against the significance value. Of all the significant genes, 72 were upregulated, and 13 genes were downregulated (green points *p* < 0.05; FC >1.5; orange points FDR adjusted *p* < 0.05; FC >1.5, labeled points FDR adjusted *p* < 0.05; FC >2). (D) Volcano plot for FFPE spinal cord tissue showing fold change against the significance value. Of all the significant genes, 62 were upregulated, and 38 genes were downregulated (green points *p* < 0.05; FC >1.5; orange points FDR adjusted *p* < 0.05; FC >1.5, labeled points FDR adjusted *p* < 0.05; FC >2).

The nCounter Neuroinflammation Panel was used to assess the spinal cord ventral horn in two tissue cohorts (one snap‐frozen and one FFPE). Significantly more genes were upregulated (76 in the frozen tissue dataset and 62 in the FFPE tissue dataset) than downregulated (13 in the frozen tissue and 38 in the FFPE tissue; Figure 1, Tables S7–S10).

The fold changes of all 770 genes (including those that were not significantly affected by disease) from the frozen spinal cord data set were significantly correlated with the fold changes from both the FFPE dataset (*R* = 0.148, *p* < 0.001) and another RNASeq spinal cord MND dataset from the literature used for validation [38] (*R* = 0.25, *p* < 0.0011). Importantly, all three datasets (our frozen and FFPE nCounter datasets and the RNAseq dataset [38]) showed upregulation of *APOE*, *TYROBP*, and *TREM2*. These three components form a pathway with a key role in inflammatory regulation and can induce a disease‐associated state in microglia (see below).

Of the 89 differentially expressed genes from the frozen dataset that were known to have cell‐specific expression [37], 50 genes were known to be primarily expressed by microglia or macrophages. The KEGG pathways associated with the differentially expressed transcripts from sporadic ALS/MND spinal cord were ranked by the number of genes associated with each pathway (see Table S11).

By Cox Proportional Hazards Regression, 23 and 17 genes were associated with longer survival in the frozen and FFPE datasets, respectively. Shorter survival was associated with 21 and 23 genes in the frozen and FFPE datasets, respectively (Tables S12–S15). Functionally, genes associated with longer survival were involved in both the adaptive and innate immune response, cytokine signaling, growth factor signaling, autophagy, and apoptosis. Genes associated with shorter survival had overlapping functions, namely innate and adaptive immunity, cytokine signaling, growth factors, and microglial function.

**FIGURE 2:**
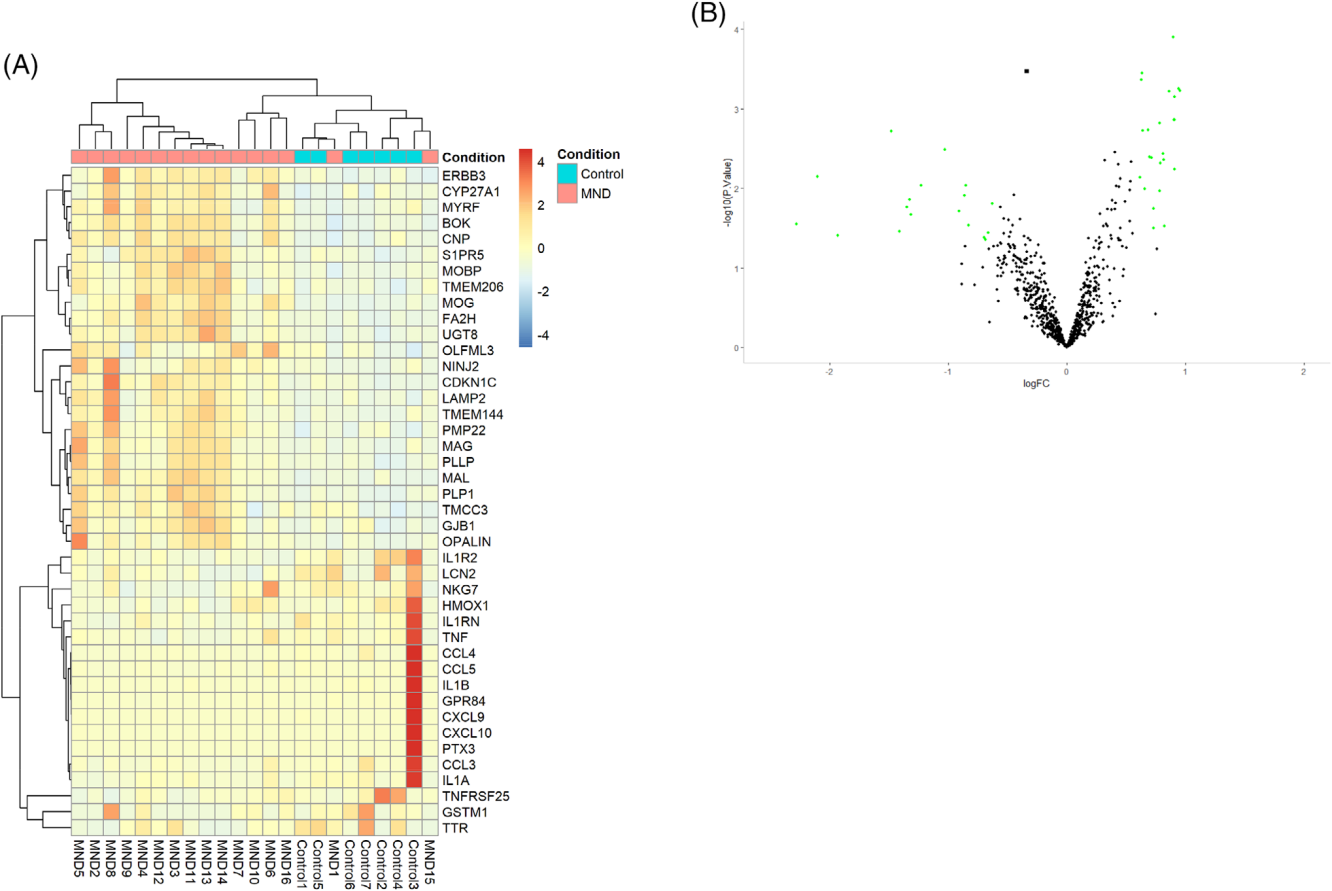
The motor cortex shows little inflammatory signaling in sMND. (A) Heatmap displaying the normalized and row‐scaled expression of 42 differentially expressed genes (*p* < 0.05 and FC >1.5) between MND and neurologically healthy cases. (B) Volcano plot showing fold change against the significance value. Green Points *p* < 0.05; FC >1.5. No genes reached significance following FDR correction.

Inflammation in the *motor cortex* was studied using the nCounter platform in one snap‐frozen tissue cohort (*n* = 16 sporadic ALS/MND cases and 8 neurologically healthy controls for each dataset). In contrast to the spinal cord, none of the 770 genes assessed were significantly altered following false discovery rate correction (Figure 2).

In summary, RNA‐based gene expression analysis shows concordance between nCounter datasets obtained from FFPE and frozen tissue, and the nCounter analyses are consistent with an archival RNA‐seq dataset from the literature. The inflammation in the spinal cord appears more severe than in the motor cortex in MND. The analysis highlighted several key inflammatory processes, including *APOE‐TYROBP/DAP12‐TREM2* signaling.

### 3.2 Immunohistochemistry for inflammatory markers in sporadic ALS/MND

Immunohistochemistry was performed to elucidate and confirm the findings from the nCounter‐based investigation above: Ionized calcium‐binding adaptor molecule 1 IBA1, a fairly universal marker of microglia and monocytes [42], MHCII/HLA‐DR (a marker of activated microglia [42], and CD68 a marker of microglial phagocytosis [43]) were assessed. In addition, Cluster of Differentiation 163 (CD163, primarily expressed by perivascular macrophages [44]) was assessed as highlighted by the nCounter data.

### 3.3 There is substantial spinal cord inflammation in sporadic ALS/MND, which is variable in anatomical extent and severity

**FIGURE 3:**
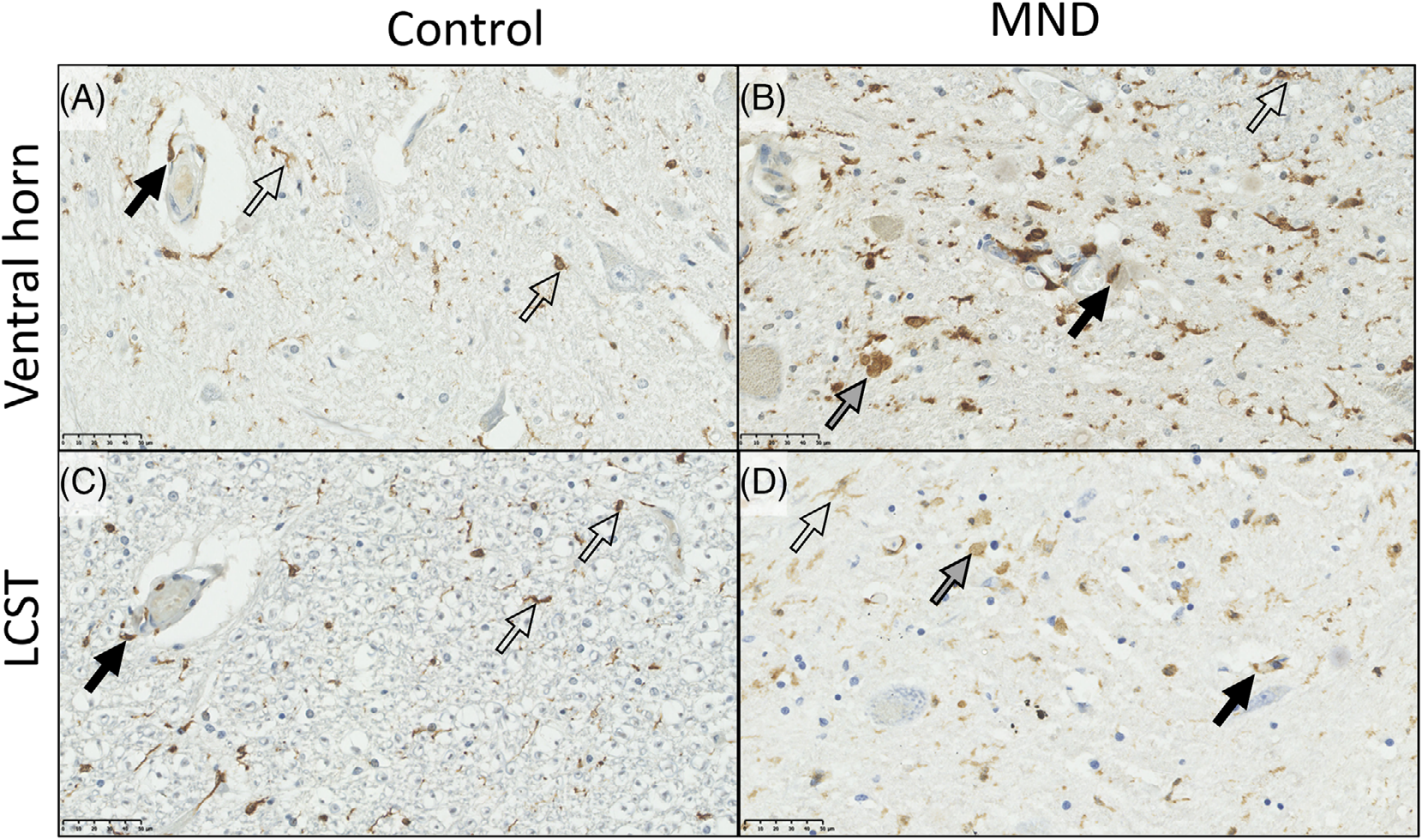
IBA1 in the spinal cord of controls and ALS/MND cases. IBA1 labels perivascular macrophages (black arrows) and microglia (open arrows). In a control spinal cord, microglia tend to be ramified. In MND spinal cord, perivascular macrophages were swollen in some cases compared to the control. Microglia tended to show a mixture of ramified (open arrows) and amoeboid (gray arrows) morphology. (A) control ventral horn; (B) MND/ALS ventral horn; (C) control lateral corticospinal tract (LCST); D, sMND/ALS corticospinal tract. Scale bars = 50 μm.

**FIGURE 4:**
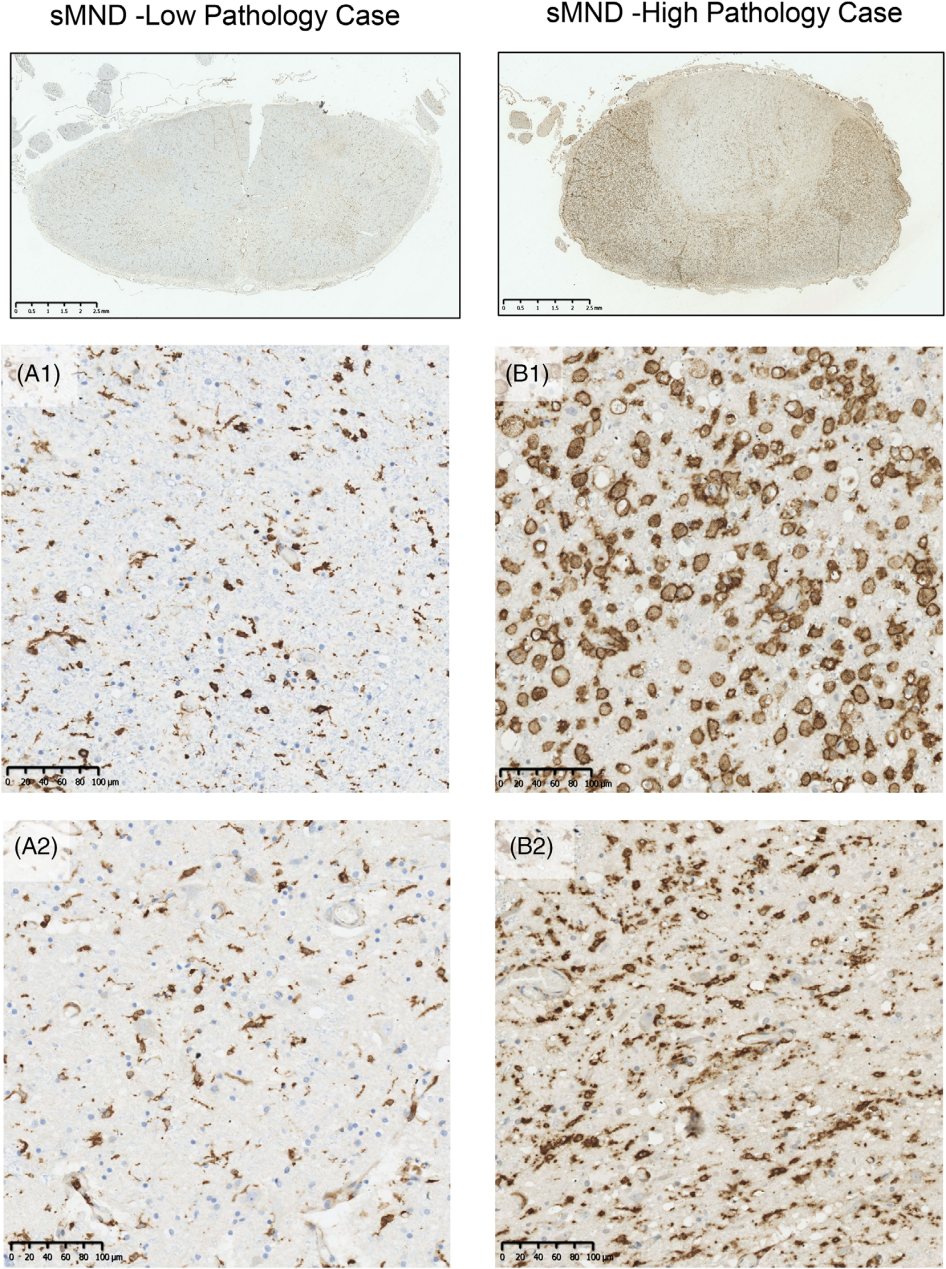
Heterogeneity of HLA‐DR immunoreactivity in sMND cases. In the spinal cord, sMND cases varied greatly in the level of HLA‐DR staining present. Both cases (A & B) are sMND cases. However, as visible from the low magnification image, these cases showed very different levels of HLA‐DR‐positive microgliosis. (A1 and B1) show higher magnification images of the lateral corticospinal tract for these cases. In the low pathology case (A1) microglia were rounded (when compared to control) and showed thickened processes. In the high pathology case (B2) microglia were completely rounded and showed much denser staining. (A2 and B2) show higher magnification images of the ventral horn. In case A (low pathology) microglia again showed thick processes, swollen cell bodies, and an increased number of HLA‐DR‐positive microglia. In case B, HLA‐DR‐positive microglia were much rounder, many becoming ameboid, and microgliosis was much more severe. A and B scale bars =2.5 mm; A1, A2, B1, and B2 scale bars =100 μm.

**FIGURE 5:**
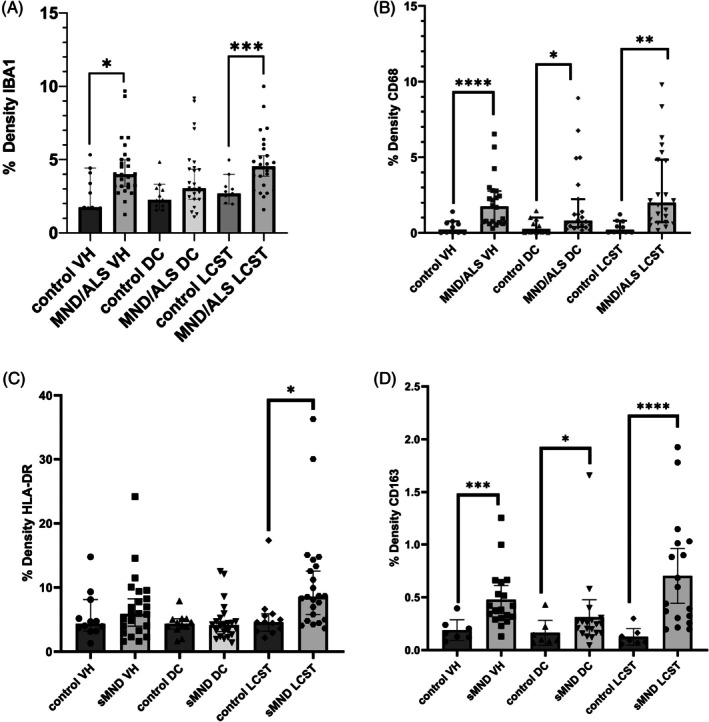
Area density for a variety of monocyte/microglial markers in MND/ALS spinal cord. There is greater inflammation in the motor structures of the cord, namely the ventral horns (VH) and lateral corticospinal tracts (LCST) in MND/ALS, as shown by IBA1 (A), CD68 (B), HLA‐DR (C), and CD163 (D). There is some lower‐level inflammation seen in the sensory dorsal columns when assessed by CD163 and CD68.

In qualitative terms, IBA1, CD68, and HLA‐DR labeled microglia and perivascular macrophages in both control and sporadic MND/ALS cases. However, in sporadic ALS/MND, IBA1^+^ microglia had a more reactive/activated morphology characterized by thicker processes and larger soma, with some cells acquiring an amoeboid form. This was most marked in the motor regions (ventral horns and corticospinal tracts; Figure 3 and Figures S3–S5). This reaction showed variation in anatomical extent, severity of inflammation, and cellular morphology within the MND cohort. While the inflammation was most prominent in motor regions, in some cases, the reaction was present throughout most of the spinal cord, albeit with lesser involvement of the dorsal columns (Figure 4). Thus, classically sensory tracts such as the spinothalamic tract were involved in some individuals. HLA‐DR showed more florid inflammation in terms of the area density of labeling (proportion of the tissue positive for this marker) compared to other markers (Figure 5C and Figure S5).

CD163 was largely confined to perivascular macrophages with minimal microglial expression in the control spinal cord (Figure S6). In MND/ALS cases, there was a variable increase in the number of positive cells in the ventral horns and corticospinal tracts both by perivascular macrophages and also by small numbers of cells within the spinal cord parenchyma.

By image analysis, the ventral horn, dorsal column, and lateral corticospinal tract showed significant upregulation of expression of IBA1, CD68, HLA‐DR, and CD163 in the lateral corticospinal tracts in MND/ALS compared to controls (Table 1). Significant upregulation was seen in the ventral horn for Iba1, CD68, and CD163, but not HLA‐DR. There was also significant upregulation in the dorsal column for CD68 and CD163 (Figure 5). MND/ALS patients with shorter survival had greater HLA‐DR expression in the ventral horns and lateral corticospinal tracts (*p* = 0.0148 and 0.005, respectively).

**TABLE 1:** Median area density in sMND/ALS and neurologically healthy controls of inflammatory markers assessed by immunohistochemistry.

Marker	Cord region	Disease status	Median area density (%)	Kruskal–Wallis H(5)	Kruskal–Wallis *p*	Post hoc Mann–Whitney *U*	Post hoc Mann–Whitney *p*
Iba1	Ventral horn	sMND/ALS	1.745	50	0.0002	61	0.03
		Control	3.999				
	Dorsal column	sMND/ALS	2.253			75	0.0963
		Control	3.04				
	Corticospinal tract	sMND/ALS	2.683			50	0.0007
		Control	4.538				
CD68	Ventral horn	sMND/ALS	0.206	32.1	<0.0001	24	<0.0001
		Control	1.761				
	Dorsal column	sMND/ALS	0.254			26	0.0139
		Control	0.798				
	Corticospinal tract	sMND/ALS	0.204			23	<0.0001
		Control	2.000				
HLA‐DR	Ventral horn	sMND/ALS	4.351	21.5	<0.001	111	0.181
		Control	5.898				
	Dorsal column	sMND/ALS	4.343			131	0.412
		Control	4.122				
	Corticospinal tract	sMND/ALS	4.581			59	0.003
		Control	8.549				
CD163	Ventral horn	sMND/ALS	0.158	36.66	<0.0001	14	0.0007
		Control	0.383				
	Dorsal column	sMND/ALS	0.100			33	0.028
		Control	0.244				
	Corticospinal tract	sMND/ALS	0.091			4	<0.0001
		Control	0.597				
APOE	Ventral horn	sMND/ALS	0.3115	25.57	<0.0001	32	0.0036
		Control	1.659				
	Dorsal column	sMND/ALS	0.0838			51	0.949
		Control	0.6082				
	Corticospinal tract	sMND/ALS	0.115			20	0.0003
		Control	1.456				
TYROBP	Ventral horn	sMND/ALS	0.496	15.51	0.008	11	0.009
		Control	2.889				
	Dorsal column	sMND/ALS	0.4715			20	0.075
		Control	1.364				
	Corticospinal tract	sMND/ALS	0.8215			12	0.018
		Control	4.148				
TREM2	Ventral horn	sMND/ALS	0.86	8.449	0.133	N/A	N/A
		Control	0.942				
	Dorsal column	sMND/ALS	0.93			N/A	N/A
		Control	0.545				
	Corticospinal tract	sMND/ALS	0.439			N/A	N/A
		Control	0.989				

### 3.4 The apolipoprotein E‐TREM2‐TYROBP pathway is upregulated in motor components of the spinal cord

**FIGURE 6:**
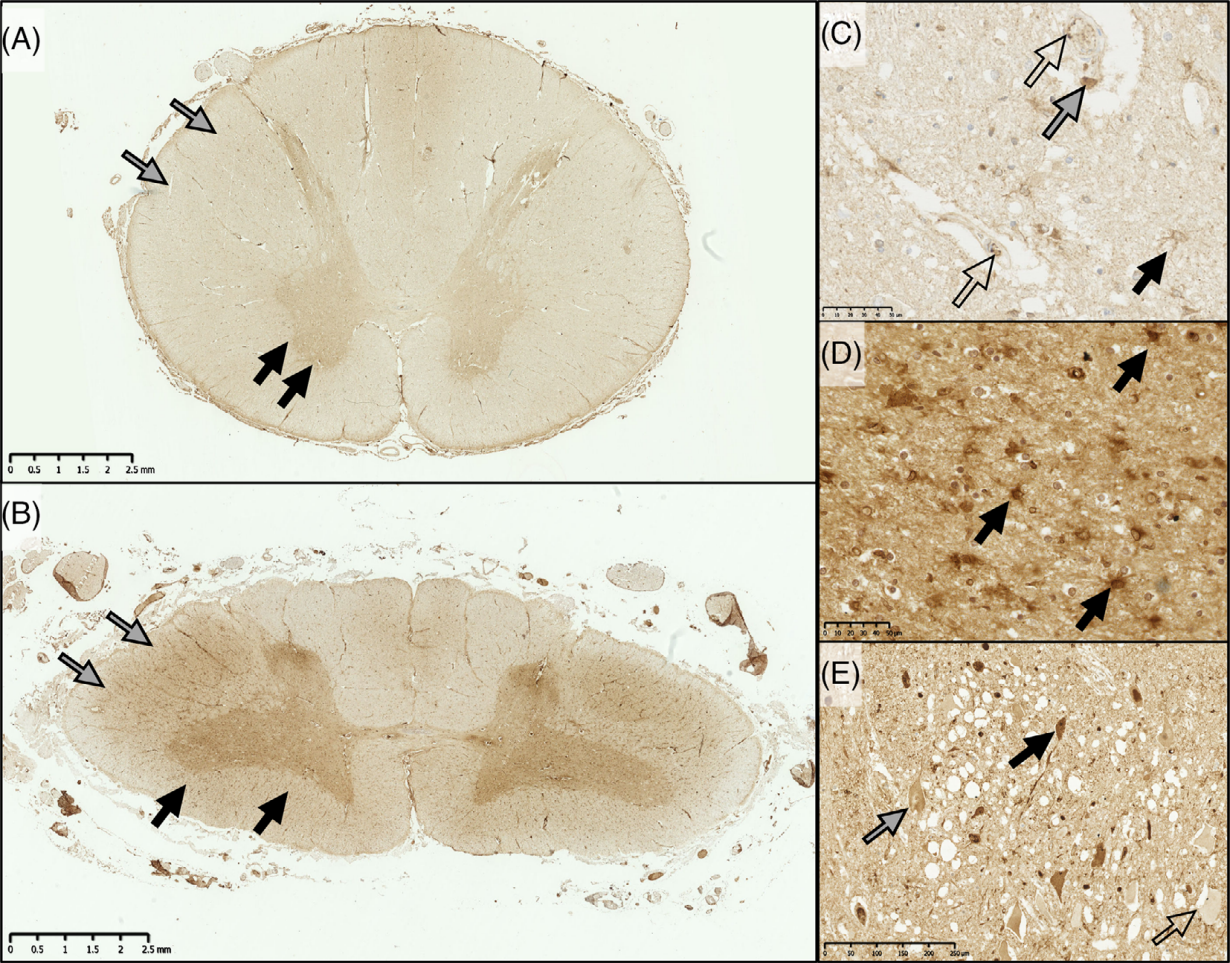
ApoE immunoreactivity in the spinal cord. In control (A) and MND/ALS (B) spinal cord, at low power, ApoE is seen in higher levels in the gray matter compared to the white matter. In MND/ALS cases, ApoE is increased in the corticospinal tract (gray arrows) and ventral horns (black arrows). At higher power (C, control ventral horn; D, MND/ALS ventral horn), ApoE is present in endothelial cells (open arrows), perivascular macrophages (gray arrows) and parenchymal glial cells (black arrows) that have the appearance of astrocytes, as well as the background neuropil. There is also variable motor neuron staining (E): Some having a high signal (black arrow), others have a similar signal to the surrounding parenchyma (gray arrows) and a small number of neurons have no immunoreactivity (open arrows). This differential ApoE expression varied greatly between cases and was not associated with disease/control status. Scale bars: A,B = 2.5 mm; B,C = 50 μm; D = 100 μm.

ApoE immunohistochemistry revealed expression throughout the spinal cord that was more marked in gray than white matter when viewed at low magnification. While there was labeling of the cord neuropil, there was especially strong labeling in glial cells predominantly of astrocytic morphology (Figure 6, Figure S7). A subset of motor neurons was also strongly stained. In MND/ALS cases, there was a greater number of glial cells expressing ApoE in the corticospinal tract and in the ventral horns. The proportion of motor neurons that were positive or negative for ApoE did not differ between MND/ALS cases and controls.

Digital image analyses showed significantly greater cellular ApoE area density in the lateral corticospinal tract (U = 20, *p* = 0.0003) and ventral horns (U = 32, *p* = 0.0036) but not in the dorsal columns in MND/ALS (U = 51, *p* = 0.0949).

**FIGURE 7:**
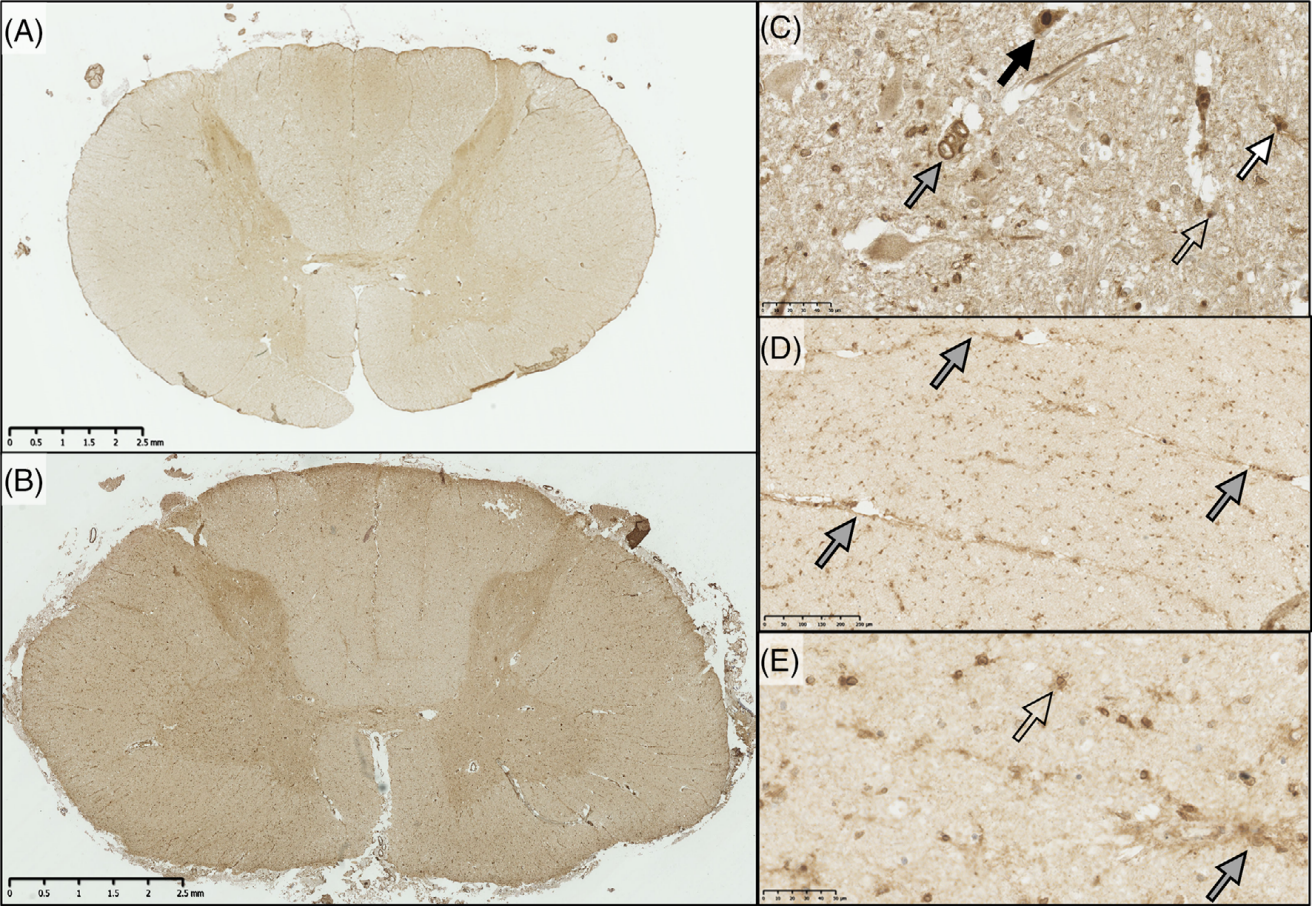
Expression of TYROBP in the spinal cord. In control (A) and MND/ALS (B) spinal cord at low magnification, TYROBP immunoreactivity was present in the neuropil, at higher levels in the gray matter compared to the white matter. Glial staining in the white matter, particularly in the corticospinal tracts, was increased in MND/ALS cases. At higher magnification (C), TYROBP‐labeled motor neurons in the ventral horn (black arrow), and blood vessels (gray arrow), as well as various ramified (white arrow) and unramified glia (open arrow). Image taken from the ventral horn. In MND/ALS ventral horn (D,E), there was greater TYROBP expression in perivascular cells. Scale bars: A,B = 2.5 mm; D = 250 μm; C,E = 50 μm.

TYROBP (also known as DAP12), showed a similar pattern of expression to ApoE, being present in neuropil, neurons, glia, and blood vessels with greater expression in gray than white matter in both control and sporadic ALS/MND groups (Figure 7, Figure S8). In blood vessels, endothelial cells were faintly labeled, with stronger labeling of the smooth muscle in the media of arterioles and arteries. Veins showed very little staining.

**FIGURE 8:**
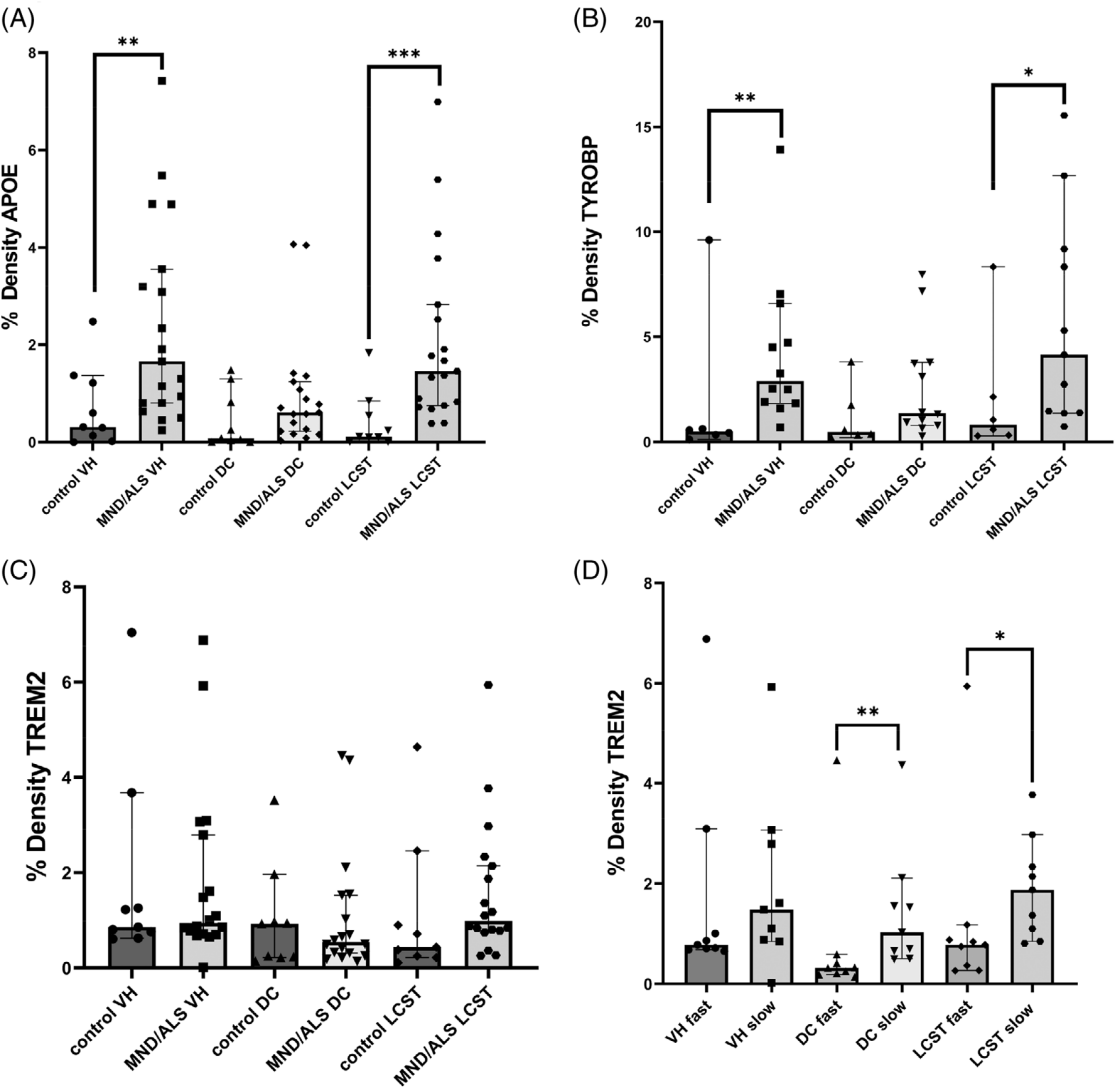
Area density for a variety of ApoE, TYROBP, and TREM2 in MND/ALS spinal cord. There is greater expression of ApoE (A) and TYROBP (B) in the motor structures of the cord, namely the ventral horns (VH) and lateral corticospinal tracts (LCST) in MND/ALS. There were no such intergroup differences in TREM2 (C). However, excess TREM2 expression was associated with slower disease progression (D).

In sporadic ALS/MND cases, microscopy revealed an increase of both perivascular macrophages and interstitial glial TYROBP staining, especially in the corticospinal tracts and the ventral horns. Ramified cells tended to be observed in the ventral horns and more amoeboid cells in the corticospinal tracts (Figure S8). Glial upregulation was especially marked in perivascular locations (Figure 7). Image analysis confirmed increased TYROBP in the ventral horns (U = 11, *p* = 0.009) and corticospinal tracts (U = 12, *p* = 0.018), but not dorsal columns in MND/ALS (U = 20, *p* = 0.075; Figure 8). There was no relationship with survival time (*p* = 0.932).

**FIGURE 9:**
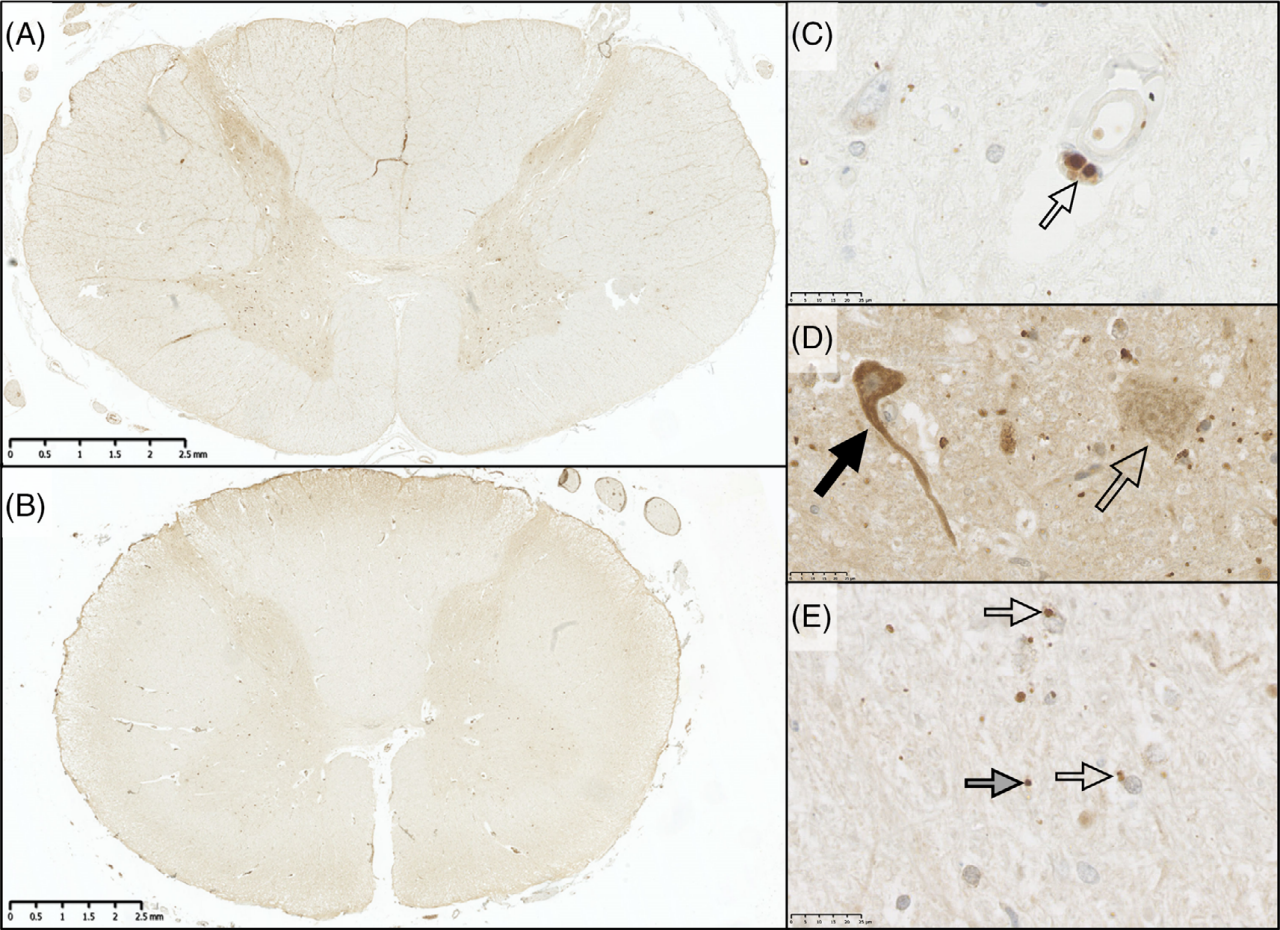
TREM2 expression was minimal in the parenchyma. TREM2 immunoreactivity was minimal in the parenchyma, although some cases did show slightly greater labeling of the gray matter compared to white. At low power, there was little difference between control cases (A) and sMND cases (B). At higher power, TREM2 was seen in a few small, rounded cells, likely perivascular macrophages (C). The majority of neurons showed minimal TREM2 signal (open arrow, D). However, TREM2 immunoreactivity did label a small number of motor neurons more strongly in the ventral horn (black arrow). These were not present in all cases and did not appear to be associated with either sMND or control cases specifically. In the parenchyma (E), small TREM2 + ve granules were observed. Some granules were associated with glial or monocyte cells (open arrow). Others were not (gray arrow). Scale bars: B = 2.5 mm; C,D,E = 25 μm.

**FIGURE 10:**
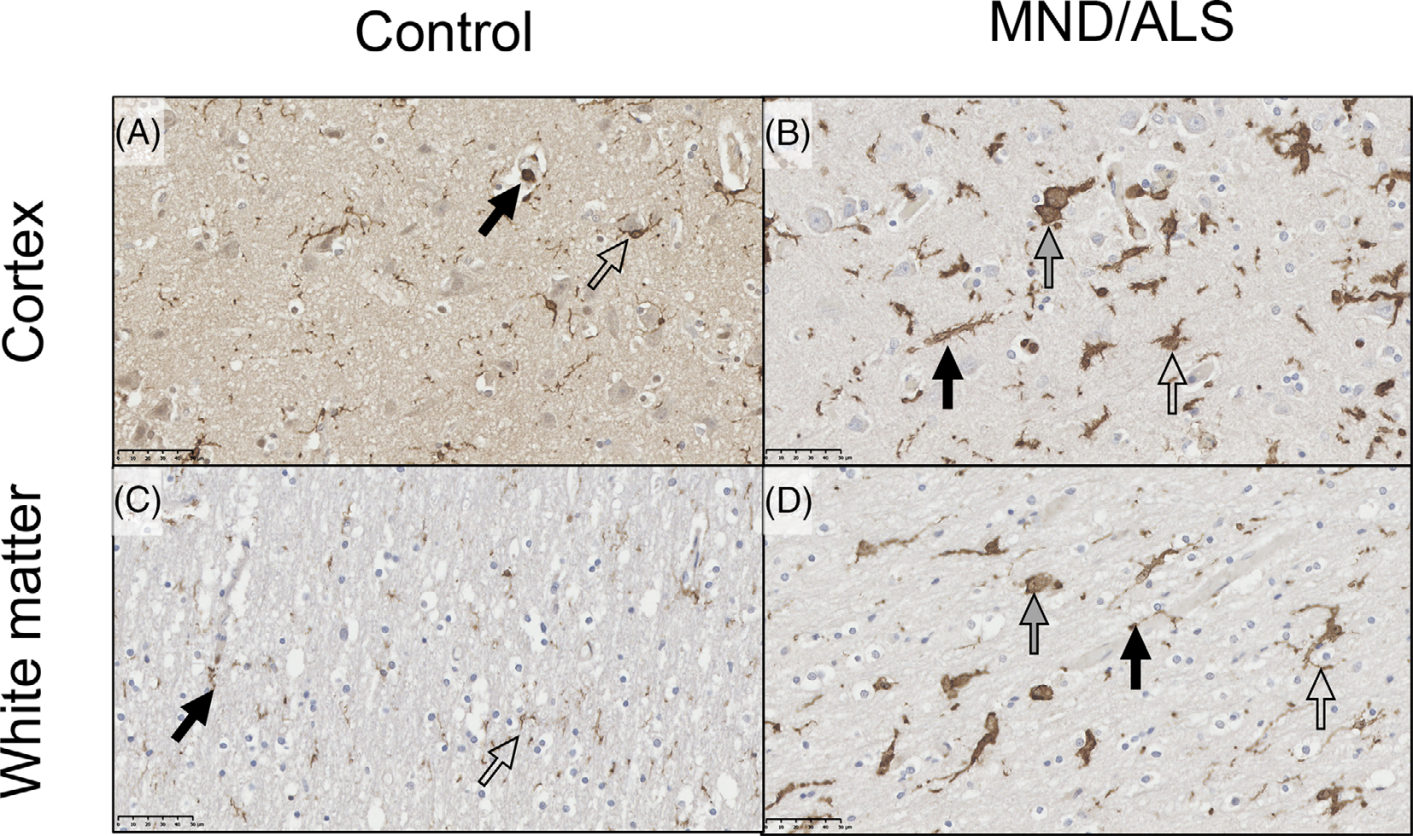
IBA1 in motor cortex. IBA1‐labeled perivascular macrophages (black arrows) and microglia (open arrows). In control cases, microglia were ramified with small cell bodies and fine processes. Similar patterns of expression were observed in the gray matter (A) and white matter (C). In MND/ALS (B, cortex; D, white matter), microglia had an activated morphology, with thicker cell bodies and swollen processes (open arrows); some microglia had also transitioned to the fully amoeboid state (gray arrows). Perivascular macrophages are labeled with black arrows. Expression was similar in gray matter (C) and white matter (D). Scale bar = 50 μm.

TREM2 expression was seen in a few perivascular macrophages, a variable proportion of motor neurons, and a few glial cells in the parenchyma (Figure 9, Figure S9). There was greater expression in the gray compared to the white matter. Control and MND/ALS cases showed similar patterns of TREM2 expression. Image analysis found no difference between groups in the degree of TREM2 expression (H(5) = 8.449, *p* = 0.133; Figure 10). There was a significant relationship between longer survival and greater expression in the white matter tracts (dorsal column, U = 11, *p* = 0.008; corticospinal tract, U = 15, *p* = 0.024), but not the ventral horn (U = 27, *p* = 0.258) by digital image analysis.

To determine which cells were expressing ApoE, TYROBP, and TREM2, sections of spinal cord from MND/ALS individuals were serially immunostained for ApoE, TYROBP, or TREM2, then IBA1 (Figure S10). This revealed that most of the cells that were strongly positive for these markers were IBA1‐positive microglia and perivascular macrophages.

### 3.5 The neuroinflammatory response in the motor cortex is less than that in the spinal cord in MND

In both sporadic ALS/MND and control cases, as was observed in the spinal cord, IBA1, CD68, and HLA‐DR label microglia and perivascular macrophages with considerable individual variation in the number of cells labeled. CD163 was largely confined to perivascular macrophages (Figure S11).

In control cases, microglia were ramified with fine processes in both white and gray matter. In sporadic ALS/MND cases, there were activated microglia with thickened processes as well as some amoeboid cells in both the white and gray matter (Figure 10). In contrast to the spinal cord, CD163 was limited to perivascular macrophages with no labeling of parenchymal cells (Figure S11). While there were morphological differences in microglia, there were no intergroup differences in the area density of IBA1, HLA‐DR, or CD163 (*p* ≥ 0.141). There was no relationship between IBA1 or HLA‐DR area density and survival (H [3]=1.015, *p* = 0.798). While Kruskal‐Wallis suggested relationships between CD68 and diagnosis (*p* = 0.0315) and survival in MND cases (*p* = 0.0006), this was not supported by post hoc tests (all *p* ≥ 0.319).

ApoE (Figure S12) and TYROBP (Figure S13) immunohistochemistry in the motor cortex showed a similar pattern to that of the spinal cord: In controls, there was variable expression in neurons, glia, and vascular structures with significant expression of ApoE in the neuropil that was less marked for TYROBP. For TYROBP, the area density in both cortex and white matter was significantly greater in sporadic ALS/MND than in control cases in both gray and white matter.

There was an apparent relationship between TYROBP staining density and patient survival by Kruskal–Wallis (H(3) = 12.44, *p* = 0.006). However, post hoc Mann–Whitney *U* tests showed no effect of survival in either gray or white matter (*p* ≥ 0.077).

In the motor cortex, TREM2 expression was largely confined to perivascular macrophages with minimal staining of neurons and negligible difference between control and MND/ALS cases (Figure S14). There were no differences between control and MND/ALS cases.

The relationship between mRNA and protein levels is often unclear [45]. For that reason, it is necessary to validate alterations seen in mRNA using protein‐based technologies. As noted above, the transcriptomic analyses of mRNA by nCounter revealed the spinal cord to be an area of much greater inflammatory signaling than the motor cortex. On that basis, we wished to investigate if this was borne out at the protein level by examining our immunohistochemistry area density data by comparing the white matter of the precentral gyrus with the lateral corticospinal tracts and the motor cortex with the spinal cord ventral horn. This revealed significantly greater area density of expression (percentage of section positively labeled for the marker in question) in the spinal cord than in the motor cortex for Iba1 (U = 7, *p* = 0.006), HLA‐DR (U = 0, *p* = 0.0001), TYROBP (U = 0, *p* = 0.002), and TREM2 (U = 0, *p* = 0.0004) for control white matter, HLA‐DR (U = 0, *p* = 0.0001), CD163 (U = 4, *p* = 0.014), and TREM2 (U = 0, *p* = 0.0004) for control gray matter, and all markers in the spinal cord compared to the precentral gyrus in the ALS/MND cases (all *p* ≤ 0.012).

### 3.6 The APOE ε3/3 variant is over‐represented within sporadic ALS/MND patients, while ε2/ε2 is under‐represented

Having established upregulation in components of the ApoE‐TYROBP/DAP12‐TREM2 pathway in MND and an association between TREM2 and disease severity (as indexed by survival time), we were interested in the genotype of *APOE*. This was assessed using data from project MinE, including 29,612 ALS patients and 122,656 controls [26].

ALS patients were significantly enriched with the APOE ε3/ε3 variant (OR = 3.6, beta = +1.29, *p* = <2e‐16) but depleted of the ε2/ε2 variant (OR = 0.74, beta = −0.3, *p* = 0.01). There was a non‐significant depletion of the ε4/ε4 genotype (OR = 0.92, beta = −0.08, *p* = 0.17; Table 2).

**TABLE 2:** Relationship between *APOE* gene variant on odds ratio (OR) for diagnosis and hazard ratio (HR) survival time and age of onset showing odds ratio enrichment of ε3/ε3 in sMND/ALS cases and ε2/ε2 in controls. ε4/ε4 is associated with shorter survival and earlier age of onset.

Genotype	sMND/ALS vs. Control	Survival	Age of onset
ε4/ε4	*p* = 0.17 (*b* = −0.08, OR = 0.92)	*p* = 0.05 (coeff = 0.2, HR = 1.2)	*p* = 0.04 (coef = 0.22, HR = 1.25)
ε4/ε3	*p* = 1.5e‐4 (*b* = −0.07, OR = 0.93)	*p* = 0.08	*p* = 0.73
ε4/ε2	None	*p* = 0.4	*p* = 0.06 (coeff = −0.18, HR = 0.84)
ε3/ε3	*p* < 2e‐16 (*b* = 1.29, OR = 3.6)	*p* = 0.32	*p* = 0.17
ε3/ε2	*p* = 5.7e‐10 (*b* = −0.1, OR = 0.90)	*p* = 0.27	*p* = 0.17
ε2/ε2	*p* = 0.01 (*b* = −0.3, OR = 0.74)	*p* = 0.63	*p* = 0.34

In a subset of ALS patients for whom survival data was available (*n* = 4897), the ε4/ε4 haplotype was associated with earlier age of ALS onset (coef = 0.22, HR = 1.25, *p* = 0.04) and a shorter survival (coef = 0.2, HR = 1.2, *p* = 0.05). Over the disease course, carrying the ε4/ε4 variant compared to not carrying the haplotype was associated with a hazard ratio (HR) of 1.25 indicating an increased risk of death. The ε3/ε3 and ε2/ε2 variants were not significantly associated with age of onset or survival.

## 4 DISCUSSION

The inflammatory mRNA profile of ALS/MND was characterized in the *post‐mortem* spinal cord and motor cortex. Two datasets were generated for the spinal cord using the nCounter platform. These were compared with a third RNAseq dataset from the literature. There was a good correlation between the three datasets, with the inflammatory response in the spinal cord greater than that in the motor cortex. A number of neuroinflammatory pathways were highlighted in the spinal cord, notably all three elements of the ApoE‐TYROBP‐TREM2 pathway.

Following this, immunohistochemistry was used to elucidate neuroinflammation at the protein level. We tested the following hypotheses on the basis of the transcriptomic data:

1. Expression of microglial and macrophage markers (IBA1, CD68, HLA‐DR, and CD163) will be increased in sporadic ALS/MND compared to controls. Microglia will display activated morphology characterized by swelling of the processes and cell body and/or the presence of amoeboid microglia in sporadic ALS/MND CNS regions. This will be more marked in the spinal cord than in the motor cortex. Within the spinal cord, there will be greater inflammation in motor regions (ventral horns and corticospinal tracts) than in sensory regions.
2. ApoE, TREM2, and TYROBP expression will be increased in the spinal cord in sporadic ALS/MND cases compared to control cases.
3. There will be a greater microglial response and increased expression of target proteins in the spinal cord overall compared to the motor cortex brain in sporadic ALS/MND cases.
4. Expression of proteins of interest will be associated with survival, particularly in the spinal cord.

This confirmed that inflammation is far greater in the spinal cord than in the motor cortex and showed marked inter‐individual variation within the sporadic ALS/MND cohort. In the spinal cord, motor structures (ventral horns and corticospinal tracts) were most severely affected, although the corticospinal tracts showed more marked inflammation than the ventral horns. Furthermore, there was involvement of extra‐motor regions (such as the spinothalamic tract) of the cord in many cases. This may be a pathological correlate of non‐motor symptoms such as pain, which are increasingly recognized [46]. The dorsal columns were the least affected but not completely spared.

The immunostaining confirmed spinal cord upregulation of ApoE and TYROBP (most pronounced in the corticospinal tracts) in sporadic ALS/MND, while higher TREM2 expression was associated with patients with longer survival times, suggesting a possible protective role for this pathway.

Data from the project MINE were accessed to assess the influence of *APOE* genotype on MND inheritance. It was found that, in contrast to Alzheimer's disease, where the ε4 haplotype is a risk factor, in MND, ε3 was a risk factor for MND, while ε2 and ε4 appear to be underrepresented in the MND population.

### 4.1 Inflammation in MND/ALS is greater in the spinal cord than in the motor cortex

While the motor cortex had less inflammation than the spinal cord, it was not completely unaffected—there were changes in microglial morphology as well as upregulated TYROBP expression. Importantly, there was considerable inter‐individual variation in the degree of inflammatory marker expression. This suggests there may be a small but variable inflammatory response that requires large sample sizes to be detected reliably by array technologies. This is consistent with the literature where a small post‐mortem study (*n* = 11 sporadic ALS/MND and 9 controls) reported little evidence for motor cortex inflammation [47]. In contrast, a larger study of 31 sporadic ALS/MND and 10 controls found a significant upregulation of genes associated with immune pathways [48].

The finding of marked spinal cord inflammation fits well with a recent transcriptomic study that highlighted an increase of microglial and inflammatory markers in the spinal cord in a large cohort of ALS/MND cases that included both sporadic and familial cases [49].

### 4.2 Disease‐associated microglia and MND/ALS

We found upregulation of TREM2 mRNA in sporadic ALS/MND, with the protein levels associated with longer survival. This is in line with transcriptomic studies that have consistently found upregulated TREM2 in human MND and have associated soluble TREM2 with neuroprotection [50, 51].

In the CSF, soluble TREM2 protein is highly expressed in the early stages of disease and diminishes with progression [51]. In late‐stage disease, soluble TREM2 expression positively correlates with survival time, suggesting this may be protective.

TREM2 is a cell surface receptor that regulates the inflammatory phenotype in myeloid cells [52]. When activated by ApoE, the cytoplasmic domain complexes with TYRO protein tyrosine kinase‐binding protein (TYROBP, also known as DAP12), which signals through an intracellular immunoreceptor tyrosine activation motif, which can result in an anti‐inflammatory phenotype and phagocytosis [53, 54, 55]. In addition, TREM2 binds TDP‐43, and TREM2 depletion in microglia causes loss of the ability to phagocytose TDP‐43 inclusions, thereby enhancing motor dysfunction [56]. An analogous phenomenon has been observed for amyloid ß animal models of Alzheimer's disease [57], leading to early‐stage trials of TREM2 agonism to treat Alzheimer's disease [36]. The possibility of using existing agents to treat MND/ALS is an exciting prospect.

TREM2 is widely expressed in the brain, and due to its expression by somatic human macrophages and confirmed murine microglial expression, it has been assumed that human microglia also express TREM2. However, while human microglia may express TREM2 mRNA [50, 58], immunohistochemistry studies have failed to find microglial TREM2 protein in human *post‐mortem* brain. In contrast, there have been demonstrations of TREM2‐positive cells in intravascular monocytes, neurons, and perivascular macrophages [59, 60, 61]. We have found TREM2 to label intravascular and perivascular macrophages as well as some neurons with few parenchymal cells expressing this protein in the motor cortex. There was a greater number of TREM2‐positive parenchymal cells in the spinal cord and increased expression of spinal cord TREM2 in ALS/MND with longer survival. The TREM2‐positive cells were also IBA1^+^ amoeboid cells, likely representing recruited macrophages, consistent with the existing literature [59].

TYROBP expression in MND has not been as widely studied. We observed a significant upregulation of TYROBP mRNA in the spinal cord, as well as TYROBP protein in the motor regions of the spinal cord and in both the white and gray matter of the precentral gyrus.

The literature to date suggests a toxic role for TYROBP: Knockdown in a mouse model of hypoglossal nerve injury resulted in reduced proinflammatory cytokine production and reduced neuron death [62]. Similarly, reduced TYROBP function in mouse models confers resistance to demyelination [63] as well as tau hyperphosphorylation and dystrophic neurites in Alzheimer's disease [64]. Finally, TYROBP deficiency in mice seems to confer resilience to Alzheimer‐type tau and amyloid ß pathology [64].

Upregulation of ApoE protein and *APOE* mRNA was detected in the spinal cord in MND, consistent with previous studies [65, 66, 67]. ApoE is a fat‐binding protein involved in lipid transport, neuronal survival and plasticity, and neurite outgrowth [68, 69, 70, 71]. In the brain, it is mostly expressed by astrocytes and microglia, with lesser expression by neurons [41, 70, 72, 73, 74, 75] consistent with our own histological observations. It is unclear whether the upregulation of ApoE reflects a reactive astrocytosis, either reflecting increased expression per astrocyte or a greater number of astrocytes. Elucidating this is an interesting avenue for downstream studies.

Extracellular ApoE protein can act as a ligand to the TREM2 receptor, triggering microglial phagocytosis [76]. Knockdown of *APOE* results in ineffective neuronal debris clearance in a model of prion pathology [77], implying a neuroprotective role for ApoE.

TREM2, TYROBP, and ApoE together form a well‐characterized pathway responsible for the disease‐associated microglial (DAM) phenotype, which has a common signature across several models of neurodegeneration, e.g., [78, 79, 80]. Through activation of TREM2 signaling, often by ApoE, TYROBP results in the downregulation of transforming growth factor β (TGFβ)‐mediated microglial genes and a simultaneous upregulation of the DAM genes (Figure 1: Discussion). These DAM‐associated markers regulate inflammation, lipid metabolism, phagocytosis, and lysosomal pathways [79, 81].

DAM has been identified in many mouse models, particularly of AD [78, 79, 82], but also including other neurodegenerative models such as ALS/MND [78, 79, 80, 83]. *Post‐mortem* studies have also found evidence of the DAM signature in AD [10, 84]. However, while this DAM phenotype has been identified in models, its relevance is only emerging in human ALS/MND. Importantly, using the R Shiny app to interrogate transcriptomic data from a recent transcriptomic study [49] highlights findings that accord with our own, namely an upregulation of all three of *TREM2*, *TYROBP*, and *APOE* in the spinal cord (*p* ≤ 0.00057) in MND after false discovery rate correction. All three correlated negatively with disease survival in the cervical (*p* ≤ 0.016) but not lumbar (*p* ≥ 0.054) cord.

### 4.3 The APOE haplotype and amyotrophic lateral sclerosis/motor neuron disease

The *APOE* ε4 haplotype is one of the most important risk factors for the development of late‐onset AD [85]. With respect to the relationship between MND and *APOE*, a 2014 meta‐analysis of 4249 MND patients and 10,397 controls from North America, Scandinavia, Europe, Israel, and Guam has found no increased risk of the ε4 haplotype (either as 4/4 or 4/X) [86]. However, a Chinese study (*n* = 683 MND patients and 369 controls) reported a modest association between MND and ε4 (odds ratio 1.42; 95% CI, 1.02–1.98; *p* = 0.02) [87]. We addressed this question in the largest cohort analyzed to date, including 29,612 ALS/MND patients and 122,656 controls, and found that MND patients were significantly enriched with the *APOE* ε3/ε3 haplotype but depleted of the ε2/ε2 and the ε4/ε4 haplotypes. Given that the ε3/ε3 haplotype is the most common in the population, we wondered whether its enrichment in ALS was actually a reflection of which patients tolerated genotyping, i.e., other genotypes may be associated with more severe disease. Consistent with this, the ε4 haplotype was underrepresented in the ALS/MND cohort and was related to more aggressive disease. This is consistent with previous studies of ε4 favoring bulbar onset disease, which itself is associated with more severe disease [88, 89].

Collectively, our *APOE* data suggest that the different haplotypes are associated with different disease phenotypes with unique clinical presentations, risks, and severity. This shift in *APOE* genotype in the ALS/MND population may be partially responsible for the altered expression levels seen [90].

### 4.4 Strengths, limitations, and future directions

This human *post‐mortem* study of ALS/MND, provides a unique set of data. The strengths of the study include the fact that this centered on human sporadic disease and is thus not hindered by questionable assumptions that mutation‐based animal and cell culture models are representative of humans or human disease. Furthermore, given the often poor relationship between mRNA and the translated effector protein, the current study has the advantage of immunohistochemical validation. This has allowed both validation at the protein level as well as anatomical and cytological mapping of the neuroinflammatory response.

However, the limitations of the study include that this is a snapshot of the end stage of the disease, and as such, it cannot assess the inflammatory status at earlier stages and cannot determine directions of causation. Nevertheless, the data support better experimental models.

We have hopefully highlighted some key features of neuroinflammation that contrast between disease and controls. A larger, more powerful study, beyond the scope of the current work, could have been more successful at finding correlates of disease severity within the ALS/MND cases.

We have avoided the assumption that sporadic and mutation‐related ALS/MND represent the same disease by focusing on sporadic disease alone. While this informs our understanding of sporadic disease, it limits our contribution to the understanding of other motor neuron diseases, such as those related to mutations of *SOD1*, *C9or72*, or *FUS*.

The corticospinal tracts run from the motor cortex to the spinal cord. Increased inflammation in the spinal cord compared to the primary motor strip may suggest retrograde axonal damage. On that basis, a more detailed study to map out the anatomical extent of neuroinflammation in greater spatial detail by examining the corticospinal tract at various levels, including the brainstem, midbrain, and internal capsule, would be informative in the future.

This is, to our knowledge, the first study to highlight the ApoE‐TREM2‐TYROBP pathway in human ALS/MND. This is an obvious candidate for therapeutic intervention, as currently performed it is already in Alzheimer's disease. However, the impact of increased expression remains to be examined in sporadic ALS/MND.

The relationship between *APOE* genotype and immune pathology is a key question. Specifically, the APOE haplotype was not available for the 16 cases investigated in this study. Unfortunately, this project was not resourced for this, and this will form the focus of future studies.

In conclusion, we have demonstrated marked and variable neuroinflammation in human sporadic MND‐TDP that is most florid in the spinal cord and significantly more subtle in the motor cortex and highlighting the APOE‐TREM2‐TYROBP pathway in particular. Finally, we have performed the most high‐powered study to date of the relationship between APOE genotype and sporadic ALS/MND and found that the ε2 and ε4 haplotypes appear protective, while the ε3 haplotype was a risk factor.

## AUTHOR CONTRIBUTIONS

Conceptualization, BAA, JES, DB, JCK, PRH, JRH; Data curation, BAA, JCK, PRH, WW, JRH; Formal analysis, BAA, JCK, WW, MD, JRH; Funding acquisition, JES, DB, PRH, JRH; Investigation, BAA, JES, CD, PRH, DF, CAM, JRH; Methodology, BAA, JES, CD, DB, JCK, PRH, DF, CAM, WW, MD, JRH; Project administration, BAA, JES, CD, DB, PRH, DF, JRH; Resources, JES, CD, PRH, DF, CAM, JRH; Software, BAA, WW, MD; Supervision, JES, PRH, JRH; Validation, BAA, CD, PRH, WW, MD; Visualization, BAA, WW, JRH; Writing—original draft, BAA, JRH; Writing—review & editing, all authors.

## FUNDING INFORMATION

The Pathological Society of Great Britain, the British Neuropathological Society, and Bruker Spatial Biology, who provided two nCounter Neuroinflammation panels.

## CONFLICT OF INTEREST STATEMENT

None.

## ETHICS STATEMENT

The majority of the data presented here formed the basis of a PhD project undertaken by BAA. The Sheffield Brain Tissue Bank (SBTB), which provided the tissue used here, has ethical permission to function as a research tissue bank. At the time the work was undertaken, this was covered by a favorable opinion from the Scotland A Research Ethics Committee (Reference 19/SS/0029). SBTB adheres to consenting protocols laid down by the UK Human Tissue Authority and agreed to by the Research Ethics Committee.

## CONSENT

SBTB adheres to consenting protocols laid down by the UK Human Tissue Authority and agreed to by the Research Ethics Committee.

## Supporting information


**Data S1.** Supplementary tables.

**Data S2.** Supplementary figure.

## Data Availability

nCounter gene expression data are available at the University of Sheffield Online Research Data facility (https://hdl.handle.net/10779/sheffield.27908097.v1).
