# Supplementary material for: Human amyotrophic lateral sclerosis/motor neuron disease: The disease‐associated microglial pathway is upregulated while APOE genotype governs risk and survival
Source: Brain Pathol. 2025 Jun 12;35(6):e70019. doi: 10.1111/bpa.70019 (PMC12488259; doi:10.1111/bpa.70019)
Supplement: Supplementary file 1 — Data S1. Supplementary tables. [file BPA-35-e70019-s002.docx]

## Supplementary table 1: FFPE Spinal Cord NanoString Cohort Details

| Case Number | Sex | Status | Onset age | Duration (months) | Age at Death (Years) | Site of Symptom Onset | Post-mortem Delay (hours) |
| --- | --- | --- | --- | --- | --- | --- | --- |
| 085/2007 | f | control |  |  | 59 |  | 5 |
| 335/1990 | f | control |  |  | 29 |  | 20 |
| 035/1996 | f | control |  |  | 87 |  | 14 |
| 014/1999 | f | control |  |  | 86 |  | 52 |
| 056/1990 | m | control |  |  | 51 |  | 25 |
| 309/1990 | m | control |  |  | 82 |  | 24 |
| 144/1991 | m | control |  |  | 65 |  | 34 |
| 098/2007 | m | control |  |  | 67 |  | 63 |
| 009/2017 | f | sALS/MND |  | 6 | 78 |  | - |
| 025/2013 | f | sALS/MND | 71 | 22 | 72 | Respiratory | 60 |
| 723/1989 | f | sALS/MND | 62 | 23 | 64 | Limb | 40 |
| 105/2007 | f | sALS/MND | 81 | 24 | 83 | Bulbar | 44 |
| 041/2008 | f | sALS/MND | 57 | 38 | 60 | - | 18 |
| 023/2010 | f | sALS/MND | 34 | 89 | 42 | Limb | 24 |
| 004/2006 | f | sALS/MND | 64 | 66 | 69 | Limb | 12 |
| 059/2009 | f | sALS/MND | 72 | 104 | 80 | Bulbar | 50 |
| 072/2005 | m | sALS/MND | 66 | 10 | 66 | Limb | 8 |
| 094/2006 | m | sALS/MND | 71 | 9 | 71 | Limb | 53 |
| 094/2009 | m | sALS/MND | 62 | 14 | 63 | Bulbar | 48 |
| 005/2010 | m | sALS/MND | 38 | 32 | 40 | Limb | 96 |
| 091/2008 | m | sALS/MND | 50 | 44 | 53 | Limb | 16 |
| 074/2009 | m | sALS/MND | 65 | 46 | 69 | Bulbar | 48 |
| 072/2002 | m | sALS/MND | 46 | 61 | 51 | Limb | 48 |
| 099/2009 | m | sALS/MND | 74 | 63 | 79 | Limb | 24 |

## Supplementary table ‎2: Frozen Spinal Cord NanoString Cohort

| Case ID | Sex | Disease Status | Onset age | Duration (months) | Age at Death (Years) | Post-mortem Delay (Hours) | Site of Symptom Onset |
| --- | --- | --- | --- | --- | --- | --- | --- |
| 085/2007 | f | control |  |  | 59 | 5 |  |
| 080/1992 | f | control |  |  | 62 | 36 |  |
| 071/1992 | f | control |  |  | 75 | 16 |  |
| 135/1982 | f | control |  |  | 89 | 24 |  |
| 109/1995 | m | control |  |  | 46 | 20 |  |
| 1028/1989 | m | control |  |  | 56 | 34 |  |
| 025/1995 | m | control |  |  | 67 | 20 |  |
| 138/1994 | m | control |  |  | 75 | 17 |  |
| 150/1993 | f | sALS/MND | 52 | 4 | 52 | 37 | limb |
| 050/2008 | f | sALS/MND | 68 | 26 | 70 | 35 | bulbar |
| 037/2000 | f | sALS/MND | 78 | 29 | 81 | 16 | bulbar |
| 041/2008 | f | sALS/MND | 57 | 38 | 60 | 18 | limb |
| 024/2008 | f | sALS/MND | 59 | 46 | 63 | 36 | limb |
| 209/1995 | f | sALS/MND | 54 | 48 | 58 | 4 | limb |
| 023/2010 | f | sALS/MND | 34 | 89 | 42 | 24 | limb |
| 059/2009 | f | sALS/MND | 72 | 104 | 80 | 50 | bulbar |
| 295/1991 | m | sALS/MND | 52 | 2 | 53 | 10 | bulbar |
| 141/2003 | m | sALS/MND | 75 | 7 | 75 | 24 | bulbar |
| 261/1990 | m | sALS/MND | 68 | 13 | 69 | 9 | bulbar |
| 024/2004 | m | sALS/MND | 58 | 19 | 60 | 4 | bulbar |
| 115/2002 | m | sALS/MND | 50 | 25 | 52 | <24 | limb |
| 034/2005 | m | sALS/MND | 60 | 38 | 63 | 16 | limb |
| 131/1995 | m | sALS/MND | 63 | 53 | 67 | 6 | limb |
| 072/2002 | m | sALS/MND | 46 | 61 | 51 | 48 | limb |

## Supplementary table ‎3: Frozen Motor Cortex NanoString Cohort

| Case ID | Sex | Disease Status | Onset age | Duration (months) | Age at Death (Years) | Post-mortem Delay (Hours) | Site of Symptom Onset | |
| --- | --- | --- | --- | --- | --- | --- | --- | --- |
| 335/1990 | f | control |  |  | 29 | 20 |  |  |
| 178/1995 | f | control |  |  | 63 | 24 |  |  |
| 072/1992 | f | control |  |  | 76 | 32 |  |  |
| 023/1992 | f | control |  |  | 84 | 39 |  |  |
| 147/1995 | m | control |  |  | 47 | 15 |  |  |
| 111/1990 | m | control |  |  | 52 | 13 |  |  |
| 293/1991 | m | control |  |  | 65 | 17 |  |  |
| 309/1990 | m | control |  |  | 82 | 24 |  |  |
| 150/1993 | f | sALS/MND | 52 | 6 | 52 | 37 | limb |  |
| 223/1999 | m | sALS/MND | 50 | 5 | 50 | 35 | multifocal |  |
| 193/1990 | f | sALS/MND | 58 | 10 | 59 | 30 | limb |  |
| 261/1990 | m | sALS/MND | 68 | 13 | 69 | 9 | bulbar |  |
| 203/1994 | f | sALS/MND | 57 | 19 | 58 | 5 | multifocal |  |
| 069/2006 | m | sALS/MND | 48 | 22 | 50 | 48 | bulbar |  |
| 050/2008 | f | sALS/MND | 68 | 26 | 70 | 35 | bulbar |  |
| 005/2010 | m | sALS/MND | 38 | 32 | 40 | 96 | limb |  |
| 041/2008 | f | sALS/MND | 57 | 38 | 60 | 18 | limb |  |
| 024/2008 | f | sALS/MND | 59 | 46 | 63 | 36 | limb |  |
| 088/1996 | m | sALS/MND | 66 | 48 | 70 | 86 | limb |  |
| 209/1995 | f | sALS/MND | 54 | 48 | 58 | 6 | limb |  |
| 131/1995 | m | sALS/MND | 63 | 53 | 67 | 6 | limb |  |
| 150/1997 | m | sALS/MND | 27 | 60 | 32 | 27 | limb |  |
| 043/2005 | m | sALS/MND | 31 | 66 | 37 | 35 | limb |  |
| 137/1996 | f | sALS/MND | 56 | 99 | 65 | 9 | multifocal |  |

## Supplementary table 4: Spinal Cord IHC cohort

| Case Number | Sex | Disease Status | Age at death | Survival time (Months) |
| --- | --- | --- | --- | --- |
| 109/1995 | M | control | 46 |  |
| 014/1999 | F | control | 86 |  |
| 098/2007 | M | control | 68 |  |
| 135/2018 | F | control | 85 |  |
| 335/1990 | F | control | 29 |  |
| 019/1991 | M | control | 54 |  |
| 118/1993 | M | control | 51 |  |
| 072/1992 | F | control | 76 |  |
| 144/1991 | M | control | 65 |  |
| 025/1995 | M | control | 67 |  |
| 023/1992 | F | control | 84 |  |
| 147/1995 | M | control | 47 |  |
| 035/1996 | F | control | 87 |  |
| 129/1994 | M | control | 63 |  |
| 261/1990 | M | sALS/MND | 69 | 13 |
| 187/1991 | M | sALS/MND | 60 | 38 |
| 175/1995 | F | sALS/MND | 75 | 13 |
| 099/2003 | M | sALS/MND | 78 | 10 |
| 043/2005 | M | sALS/MND | 31 | 66 |
| 042/2005 | M | sALS/MND | 60 | 57 |
| 049/2005 | F | sALS/MND | 61 | 42 |
| 049/2006 | M | sALS/MND | 91 | 97 |
| 088/2006 | F | sALS/MND | 51 | 23 |
| 027/2008 | F | sALS/MND | 70 | 44 |
| 075/2008 | F | sALS/MND | 61 | 82 |
| 096/2008 | F | sALS/MND | 69 | 50 |
| 098/2008 | F | sALS/MND | 75 | 50 |
| 059/2009 | F | sALS/MND | 80 | 104 |
| 099/2009 | M | sALS/MND | 79 | 63 |
| 005/2010 | M | sALS/MND | 41 | 32 |
| 023/2010 | F | sALS/MND | 41 | 89 |
| 041/2010 | F | sALS/MND | 69 | 38 |
| 064/2010 | M | sALS/MND | 79 | 24 |
| 082/2010 | F | sALS/MND | 75 | 20 |
| 005/2011 | F | sALS/MND | 76 | 52 |
| 036/2012 | M | sALS/MND | 65 | 19 |
| 048/2012 | M | sALS/MND | 43 | 34 |
| 054/2015 | F | sALS/MND | 62 | 20 |
| 060/2016 | M | sALS/MND | 75 | 26 |
| 046/2013 | M | sALS/MND | 78 | 11 |
| 058/2012 | F | sALS/MND | 65 | 62 |
| 008/2013 | M | sALS/MND | 71 | 17 |

## Supplementary table 5: Motor Cortex IHC cohort.

| Case Number | Block Number | Sex | Disease Status | Age at death | Suvival time (Months) | Post-mortem Delay |
| --- | --- | --- | --- | --- | --- | --- |
| 224/2016 | a28 | F | control | 82 | - |  |
| 135/2018 | a3 | F | control | 85 | 72 |  |
| 005/2007 | af | M | control | 63 | - |  |
| 085/2007 | b & c | F | control | 59 | 5 |  |
| 058/2022 | a19 | F | control | 68 | 91 |  |
| 018/2009 | o | M | control | 69 | - |  |
| 027/2008 | a | F | sALS/MND | 70 | 44 | 24 |
| 043/2005 | b | M | sALS/MND | 31 | 66 | 35 |
| 087/1992 | a2 | M | sALS/MND | 72 | 48 | 18 |
| 088/2006 | c | F | sALS/MND | 49 | 23 | 24 |
| 034/2005 | l | M | sALS/MND | 60 | 38 | 16 |
| 099/2009 | b | M | sALS/MND | 74 | 63 | 24 |
| 042/2005 | e | M | sALS/MND | 60 | 57 | 9 |
| 064/2009 | a | M | sALS/MND | 63 | 43 | 30 |
| 098/2008 | c | F | sALS/MND | 71 | 50 | 24 |
| 096/2008 | g | F | sALS/MND | 67 | 21 | 40 |
| 014/2008 | j | F | sALS/MND | 73 | 31 | 48 |
| 075/2008 | d | F | sALS/MND | 55 | 82 | 24 |
| 099/2003 | b | M | sALS/MND | 78 | 10 | - |
| 023/2010 | c | F | sALS/MND | 34 | 89 | 24 |
| 041/2010 | d | F | sALS/MND | 65 | 38 | 72 |
| 054/2015 | a1 | F | sALS/MND | 76 | 20 | 96 |
| 064/2010 | c | M | sALS/MND | 76 | 24 | 30 |
| 082/2010 | a | F | sALS/MND | 73 | 20 | 48 |
| 005/2010 | c | F | sALS/MND | 71 | 17 | 48 |
| 094/2006 | a | M | sALS/MND | 71 | 9 | 53 |
| 008/2013 | a23 | F | sALS/MND | 71 | 17 | 48 |
| 005/2011 | a | F | sALS/MND | 71 | 52 | 40 |
| 036/2012 | a4 | M | sALS/MND | 58 | 19 | 24 |
| 048/2012 | a3 | M | sALS/MND | 39 | 34 | 48 |
| 046/2013 | a3 | M | sALS/MND | 78 | 11 | 48 |
| 058/2012 | a4 | F | sALS/MND | 65 | 62 | 24 |
| 049/2006 | e | M | sALS/MND | 83 | 97 | 44 |
| 060/2016 | a4 | M | sALS/MND | 75 | 26 | 96 |
| 054/2005 | a | M | sALS/MND | 80 | 24 | 96 |
| 049/2005 | a | M | sALS/MND | 91 | 97 | 44 |
| 261/1990 | x1 | M | sALS/MND | 68 | 13 | 9 |
| 175/1995 | u | F | sALS/MND | 74 | 13 | 48 |
| 187/1991 | b1 | M | sALS/MND | 60 | 38 | 39 |
| 104/2004 | b | M | sALS/MND | 63 | 26 | - |
| 168/2018 | a1 | F | sALS/MND | 66 | 26 | 120 |
| 062/2016 | a1 | M | sALS/MND | 72 | 24 | 96 |
| 138/2014 | a1 | M | sALS/MND | 78 | - | 100+ |
| 055/2012 | a1 | F | sALS/MND | 71 | - | 48 |
| 077/2011 | a | M | sALS/MND | 60 | 21 | 66 |
| 014/2011 | c | M | sALS/MND | 49 | 28 | 40 |
| 128/2014 | a1 | F | sALS/MND | 29 | 65 | 96 |
| 046/2017 | b | - | sALS/MND | - | - | - |
| 086/2008 | b | M | sALS/MND | 64 | 21 | 46 |
| 050/2008 | b | F | sALS/MND | 68 | 26 | 35 |
| 024/2008 | a | F | sALS/MND | 59 | 46 | 36 |
| 105/2014 | a4 | M | sALS/MND | - | - | 80+ |
| 009/2008 | d | M | sALS/MND | 76 | 11 | - |
| 187/1996 | w | F | sALS/MND | 51 | 32 | 5 |
| 066/1996 | j1 | F | sALS/MND | 70 | 37 | 10 |
| 141/1993 | a1 | F | sALS/MND | 66 | 21 | 13 |
| 345/1990 | x1 | M | sALS/MND | 68 | 18 | 11 |
| 026/1994 | p1 | M | sALS/MND | 56 | 32 | - |
| 223/2005 | l1 | - | sALS/MND | - | - | - |
| 082/1999 | n1 | - | sALS/MND | - | - | - |
| 150/1997 | e1 | M | sALS/MND | 27 | 60 | 39 |
| 015/1998 | h1 | F | sALS/MND | 86 | 5 | 13 |
| 212/1999 | j1 | M | sALS/MND | 60 | 40 | 32 |
| 086/2006 | 4b | M | sALS/MND | 49 | 5 | 55 |
| 200/1997 | e1 | M | sALS/MND | 69 | 47 | 18 |
| 072/2005 | b | M | sALS/MND | 66 | 10 | 8 |
| 113/2008 | a | M | sALS/MND | 49 | 29 | 48 |
| 025/2013 | a3 | F | sALS/MND | 72 | 22 | 60 |
| 026/2013 | A2 | M - | sALS/MND | 62 | 15 | 90 |

## Supplementary table 6: Antibodies used for Immunohistochemistry

| Protein of Interest | Manufacturer | Catalogue Reference | Species | Clone Name | Isotype | Clone | Optimised Antigen Retrieval Conditions | Optimised Primary Antibody Concentration |
| --- | --- | --- | --- | --- | --- | --- | --- | --- |
| APOE | Abcam | ab1906 | Mouse | D6E10 | IgG1 | Monoclonal | Pressure Cooker with Borg Decloaker buffer (pH 9.5) | 1:1000 |
| CD163 | BioRad | MCA1853 | Mouse | edhu1 | IgG1 | Monoclonal | Pressure Cooker with Reveal buffer (pH 6) | 1:1000 |
| CD68 | DAKO | M0876 | Mouse | PGM1 | IgG3 | Monoclonal | Microwave with TSC (pH6) buffer | 1:100 |
| CTSS | Sigma | HPA002988 | Rabbit | - | IgG | Polyclonal | Pressure Cooker with Reveal buffer (pH 6) | 1:800 |
| HLA-DR | DAKO | m0746 | Mouse | TAL.1B5 | IgG | Monoclonal | Pressure Cooker with Reveal buffer (pH 6) | 1:100 |
| IBA1 | Abcam | ab5076 | Goat | - | IgG | Polyclonal | Microwave with TSC (pH6) buffer | 1:200 |
| TREM2 | LSBio | LS-B16999 | Mouse | - | IgG | Monoclonal | Pressure Cooker with Reveal buffer (pH 6) | 1:1600 |
| TYROBP | Thermofisher | PA5-83577 | Rabbit | - | IgG | Polyclonal | Pressure Cooker with Borg Decloaker buffer (pH 9.5) | 1:100 |

## Supplementary table 7: Genes Upregulated in MND/ALS Frozen Spinal Cord.

| *Gene Name* | *Gene Symbol* | *Log2 Fold Change* | *Fold Change* | *P Value* | *Q* |
| --- | --- | --- | --- | --- | --- |
| Serpin Family A Member 3 | SERPINA3 | 2.367 | 5.157 | <0.001 | 0.004 |
| C-C Motif Chemokine Ligand 2 | CCL2 | 2.330 | 5.029 | <0.001 | 0.004 |
| Suppressor Of Cytokine Signalling 3 | SOCS3 | 2.143 | 4.416 | 0.003 | 0.025 |
| IFI30 Lysosomal Thiol Reductase | IFI30 | 2.073 | 4.208 | <0.001 | <0.001 |
| macrophage scavenger receptor 1 | *MSR1* | 3.568 | 1.835 | <0.001 | <0.001 |
| Fc fragment of IgG receptor Ia | *FCGR1A* | 3.561 | 1.832 | <0.001 | 0.008 |
| complement C1q B chain | *C1QB* | 3.422 | 1.775 | <0.001 | <0.001 |
| cyclin dependent kinase inhibitor 1A | *CDKN1A* | 3.270 | 1.709 | <0.001 | 0.003 |
| Fc fragment of IgG receptor IIIa | *FCGR3A* | 3.105 | 1.635 | <0.001 | 0.004 |
| formyl peptide receptor 1 | *FPR1* | 2.988 | 1.579 | <0.001 | 0.008 |
| MAF bZIP transcription factor B | *MAFB* | 2.937 | 1.555 | <0.001 | 0.001 |
| complement C1q C chain | *C1QC* | 2.880 | 1.526 | <0.001 | <0.001 |
| Fc fragment of IgG receptor IIb | *FCGR2B* | 2.844 | 1.508 | 0.001 | 0.015 |
| guanylate binding protein 2 | *GBP2* | 2.653 | 1.408 | <0.001 | 0.004 |
| cathepsin S | *CTSS* | 2.623 | 1.391 | <0.001 | 0.001 |
| triggering receptor expressed on myeloid cells 2 | *TREM2* | 2.536 | 1.342 | <0.001 | 0.001 |
| CD14 molecule | *CD14* | 2.479 | 1.310 | 0.001 | 0.010 |
| vimentin | *VIM* | 2.474 | 1.307 | <0.001 | 0.001 |
| BCL2 related protein A1 | *BCL2A1* | 2.436 | 1.284 | 0.003 | 0.025 |
| transmembrane immune signaling adaptor TYROBP | *TYROBP* | 2.418 | 1.274 | <0.001 | 0.001 |
| Fc fragment of IgE receptor Ig | *FCER1G* | 2.417 | 1.273 | <0.001 | 0.001 |
| complement C3 | *C3* | 2.405 | 1.266 | <0.001 | 0.002 |
| ceruloplasmin | *CP* | 2.400 | 1.263 | 0.002 | 0.021 |
| membrane spanning 4-domains A4A | *MS4A4A* | 2.313 | 1.210 | <0.001 | 0.007 |
| SLAM family member 8 | *SLAMF8* | 2.309 | 1.207 | 0.001 | 0.014 |
| toll like receptor 2 | *TLR2* | 2.286 | 1.193 | <0.001 | 0.002 |
| TNF receptor superfamily member 1B | *TNFRSF1B* | 2.270 | 1.182 | <0.001 | 0.007 |
| Cluster of Differentiation 163 | *CD163* | 2.265 | 1.179 | 0.005 | 0.042 |
| TIMP metallopeptidase inhibitor 1 | *TIMP1* | 2.249 | 1.169 | <0.001 | 0.005 |
| protein tyrosine phosphatase receptor type C | *PTPRC* | 2.208 | 1.143 | <0.001 | <0.001 |
| annexin A1 | *ANXA1* | 2.176 | 1.122 | 0.001 | 0.009 |
| CD68 molecule | *CD68* | 2.167 | 1.116 | <0.001 | 0.001 |
| heme oxygenase 1 | *HMOX1* | 2.126 | 1.088 | 0.002 | 0.021 |
| CD84 molecule | *CD84* | 2.120 | 1.084 | <0.001 | 0.002 |
| potassium two pore domain channel subfamily K member 13 | *KCNK13* | 2.078 | 1.055 | <0.001 | 0.008 |
| LYN proto-oncogene, Src family tyrosine kinase | *LYN* | 2.046 | 1.033 | <0.001 | 0.008 |
| epithelial membrane protein 1 | *EMP1* | 2.045 | 1.032 | 0.001 | 0.012 |
| caspase 1 | *CASP1* | 2.034 | 1.024 | <0.001 | 0.003 |
| caspase 4 | *CASP4* | 2.022 | 1.016 | <0.001 | 0.007 |
| CD86 molecule | *CD86* | 2.006 | 1.004 | <0.001 | 0.005 |
| Fos proto-oncogene, AP-1 transcription factor subunit | *FOS* | 1.993 | 0.995 | <0.001 | 0.001 |
| complement C1q A chain | *C1QA* | 1.976 | 0.982 | 0.001 | 0.009 |
| STEAP4 metalloreductase | *STEAP4* | 1.970 | 0.978 | <0.001 | 0.008 |
| G protein-coupled receptor 183 | *GPR183* | 1.944 | 0.959 | 0.002 | 0.024 |
| serpin family F member 1 | *SERPINF1* | 1.920 | 0.941 | <0.001 | 0.007 |
| MYC proto-oncogene, bHLH transcription factor | *MYC* | 1.913 | 0.936 | 0.001 | 0.010 |
| leukocyte associated immunoglobulin like receptor 1 | *LAIR1* | 1.897 | 0.924 | <0.001 | 0.008 |
| sialic acid binding Ig like lectin 8 | *SIGLEC8* | 1.897 | 0.924 | 0.003 | 0.027 |
| RELB proto-oncogene, NF-kB subunit | *RELB* | 1.856 | 0.892 | <0.001 | 0.007 |
| Vav guanine nucleotide exchange factor 1 | *VAV1* | 1.843 | 0.882 | 0.001 | 0.012 |
| complement C3a receptor 1 | *C3AR1* | 1.841 | 0.880 | 0.001 | 0.010 |
| Serglycin | *SRGN* | 1.812 | 0.858 | 0.004 | 0.037 |
| Fas cell surface death receptor | *FAS* | 1.802 | 0.849 | <0.001 | 0.002 |
| dedicator of cytokinesis 2 | *DOCK2* | 1.798 | 0.847 | <0.001 | 0.005 |
| CD74 molecule | *CD74* | 1.780 | 0.832 | 0.001 | 0.018 |
| transforming growth factor beta receptor 1 | *TGFBR1* | 1.768 | 0.822 | 0.003 | 0.027 |
| paired immunoglobin like type 2 receptor alpha | *PILRA* | 1.765 | 0.820 | 0.003 | 0.029 |
| podoplanin | *PDPN* | 1.760 | 0.815 | 0.007 | 0.049 |
| TNF receptor superfamily member 1A | *TNFRSF1A* | 1.710 | 0.774 | <0.001 | 0.007 |
| matrix metallopeptidase 14 | *MMP14* | 1.702 | 0.768 | 0.001 | 0.017 |
| TNF superfamily member 13b | *TNFSF13B* | 1.702 | 0.767 | 0.003 | 0.027 |
| sphingosine-1-phosphate receptor 3 | *S1PR3* | 1.698 | 0.763 | 0.001 | 0.015 |
| leukocyte immunoglobulin like receptor B4 | *LILRB4* | 1.674 | 0.743 | 0.001 | 0.014 |
| oncostatin M receptor | *OSMR* | 1.654 | 0.726 | 0.005 | 0.042 |
| cytohesin 1 interacting protein | *CYTIP* | 1.621 | 0.697 | 0.002 | 0.025 |
| lamin B1 | *LMNB1* | 1.619 | 0.695 | 0.003 | 0.027 |
| protein tyrosine phosphatase non-receptor type 6 | *PTPN6* | 1.615 | 0.692 | 0.004 | 0.036 |
| solute carrier family 2 member 5 | *SLC2A5* | 1.601 | 0.679 | 0.003 | 0.027 |
| damage specific DNA binding protein 2 | *DDB2* | 1.601 | 0.679 | 0.003 | 0.027 |
| DAB adaptor protein 2 | *DAB2* | 1.599 | 0.678 | <0.001 | 0.008 |
| phosphatidylinositol-4,5-bisphosphate 3-kinase catalytic subunit gamma | *PIK3CG* | 1.574 | 0.654 | 0.003 | 0.027 |
| baculoviral IAP repeat containing 3 | *BIRC3* | 1.573 | 0.653 | 0.005 | 0.042 |
| WASP actin nucleation promoting factor | *WAS* | 1.533 | 0.616 | 0.001 | 0.016 |
| caspase 8 | *CASP8* | 1.516 | 0.600 | 0.007 | 0.049 |
| nucleotide binding oligomerization domain containing 1 | *NOD1* | 1.513 | 0.597 | 0.001 | 0.013 |
| solute carrier organic anion transporter family member 2B1 | *SLCO2B1* | 1.501 | 0.586 | 0.003 | 0.027 |

## Supplementary table 8: Genes Downregulated in sMND Frozen Spinal Cord

| *Gene Name* | *Gene Symbol* | *Log_2_ Fold Change* | *Fold Change* | *P Value* | *Q* |
| --- | --- | --- | --- | --- | --- |
| Cdk5 and Abl enzyme substrate 1 | *CABLES1* | -2.165 | -1.114 | <0.001 | 0.001 |
| myogenesis regulating glycosidase (putative) | *MYORG* | -1.911 | -0.935 | 0.001 | 0.012 |
| NACHT and WD repeat domain containing 1 | *NWD1* | -1.822 | -0.865 | 0.001 | 0.018 |
| tubulin beta 3 class III | *TUBB3* | -1.773 | -0.826 | <0.001 | 0.005 |
| epidermal growth factor receptor | *EGFR* | -1.749 | -0.806 | <0.001 | 0.007 |
| transmembrane protein 100 | *TMEM100* | -1.741 | -0.800 | <0.001 | 0.007 |
| solute carrier family 6 member 1 | *SLC6A1* | -1.726 | -0.787 | 0.002 | 0.022 |
| phospholipase A2 group V | *PLA2G5* | -1.714 | -0.778 | 0.002 | 0.020 |
| potassium inwardly rectifying channel subfamily J member 10 | *KCNJ10* | -1.712 | -0.775 | <0.001 | 0.008 |
| LFNG O-fucosylpeptide 3-beta-N-acetylglucosaminyltransferase | *LFNG* | -1.685 | -0.753 | 0.003 | 0.027 |
| brain enriched myelin associated protein 1 | *BCAS1* | -1.618 | -0.695 | 0.002 | 0.025 |
| microtubule associated protein 1 light chain 3 alpha | *MAP1LC3A* | -1.584 | -0.664 | 0.002 | 0.024 |
| myelin associated oligodendrocyte basic protein | *MOBP* | -1.509 | -0.594 | 0.006 | 0.045 |

## Supplementary table 9: Upregulated Genes from MND/ALS FFPE Spinal Cord

| *Gene Name* | *Gene Symbol* | *Fold Change* | *P* | *Q* |
| --- | --- | --- | --- | --- |
| serpin family A member 3 | SERPINA3 | 6.427 | <0.001 | 0.001 |
| secreted phosphoprotein 1 | SPP1 | 4.744 | <0.001 | 0.001 |
| superoxide dismutase 2 | SOD2 | 3.958 | 0.001 | 0.015 |
| vimentin | VIM | 3.926 | <0.001 | 0.001 |
| Fc fragment of IgG receptor IIIa | FCGR3A | 3.662 | <0.001 | 0.007 |
| apolipoprotein E | APOE | 3.330 | <0.001 | 0.002 |
| annexin A1 | ANXA1 | 3.122 | <0.001 | 0.005 |
| complement C1q B chain | C1QB | 3.058 | <0.001 | 0.002 |
| complement C1q C chain | C1QC | 2.952 | <0.001 | 0.001 |
| complement C1q A chain | C1QA | 2.951 | <0.001 | 0.001 |
| IFI30 lysosomal thiol reductase | IFI30 | 2.945 | <0.001 | <0.001 |
| CD74 molecule | CD74 | 2.917 | 0.001 | 0.012 |
| fatty acid binding protein 5 | FABP5 | 2.874 | <0.001 | 0.005 |
| ribosomal protein S2 | RPS2 | 2.866 | <0.001 | 0.003 |
| Fc fragment of IgE receptor Ig | FCER1G | 2.822 | <0.001 | <0.001 |
| S100 calcium binding protein A10 | S100A10 | 2.723 | <0.001 | 0.004 |
| epithelial membrane protein 1 | EMP1 | 2.719 | <0.001 | 0.007 |
| complement C4A (Rodgers blood group) | C4A | 2.671 | <0.001 | 0.003 |
| moesin | MSN | 2.655 | <0.001 | 0.007 |
| ribosomal protein L28 | RPL28 | 2.652 | 0.001 | 0.011 |
| CD44 molecule (Indian blood group) | CD44 | 2.514 | 0.003 | 0.028 |
| transmembrane immune signalling adaptor TYROBP | TYROBP | 2.468 | <0.001 | 0.001 |
| DAB adaptor protein 2 | DAB2 | 2.444 | <0.001 | 0.001 |
| lysosomal associated membrane protein 2 | LAMP2 | 2.437 | 0.001 | 0.018 |
| coagulation factor III, tissue factor | F3 | 2.431 | 0.001 | 0.018 |
| calreticulin | CALR | 2.411 | 0.001 | 0.012 |
| peroxiredoxin 1 | PRDX1 | 2.384 | 0.001 | 0.015 |
| Cluster of Differentiation 163 | CD163 | 2.370 | 0.006 | 0.047 |
| complement C3 | C3 | 2.314 | <0.001 | 0.003 |
| integrin subunit alpha 7 | ITGA7 | 2.275 | <0.001 | 0.001 |
| ribosomal protein S10 | RPS10 | 2.250 | <0.001 | 0.003 |
| cathepsin S | CTSS | 2.233 | <0.001 | 0.004 |
| FYN proto-oncogene, Src family tyrosine kinase | FYN | 2.197 | <0.001 | 0.009 |
| bromodomain containing 4 | BRD4 | 2.168 | 0.001 | 0.011 |
| sequestosome 1 | SQSTM1 | 2.164 | 0.001 | 0.012 |
| gelsolin | GSN | 2.159 | 0.001 | 0.019 |
| triggering receptor expressed on myeloid cells 2 | TREM2 | 2.158 | <0.001 | 0.002 |
| solute carrier family 1 member 3 | SLC1A3 | 2.117 | 0.002 | 0.023 |
| lysosomal associated membrane protein 1 | LAMP1 | 2.091 | 0.003 | 0.029 |
| NF-κB inhibitor alpha | NFKBIA | 2.064 | 0.001 | 0.017 |
| ribosomal protein S3 | RPS3 | 2.061 | 0.005 | 0.044 |
| TIMP metallopeptidase inhibitor 1 | TIMP1 | 2.017 | 0.005 | 0.044 |
| eukaryotic translation initiation factor 1 | EIF1 | 2.015 | 0.006 | 0.046 |
| angiotensinogen | AGT | 2.011 | 0.004 | 0.033 |
| coactosin like F-actin binding protein 1 | COTL1 | 1.988 | <0.001 | 0.003 |
| major vault protein | MVP | 1.986 | 0.001 | 0.012 |
| ribosomal protein L29 | RPL29 | 1.980 | 0.006 | 0.046 |
| RAB7A, member RAS oncogene family | RAB7A | 1.955 | 0.001 | 0.012 |
| proteasome 20S subunit beta 8 | PSMB8 | 1.955 | <0.001 | 0.011 |
| granulin precursor | GRN | 1.949 | <0.001 | 0.011 |
| integrin subunit alpha V | ITGAV | 1.932 | 0.003 | 0.029 |
| junctional adhesion molecule 2 | JAM2 | 1.889 | 0.001 | 0.013 |
| ATPase H+ transporting V0 subunit e1 | ATP6V0E1 | 1.870 | 0.001 | 0.019 |
| structural maintenance of chromosomes 1A | SMC1A | 1.816 | 0.002 | 0.019 |
| BCL2 associated agonist of cell death | BAD | 1.800 | 0.002 | 0.019 |
| macrophage scavenger receptor 1 | MSR1 | 1.730 | 0.002 | 0.027 |
| mitochondrial antiviral signalling protein | MAVS | 1.691 | 0.004 | 0.033 |
| BCL2 associated X, apoptosis regulator | BAX | 1.658 | 0.004 | 0.038 |
| AKT serine/threonine kinase 1 | AKT1 | 1.644 | 0.003 | 0.032 |
| lysine methyltransferase 2C | KMT2C | 1.631 | 0.006 | 0.046 |
| TNF receptor superfamily member 1A | TNFRSF1A | 1.630 | 0.003 | 0.029 |

## Supplementary table 10: Down Regulated Genes from MND/ALS FFPE Spinal Cord

| *Gene Name* | *Gene Symbol* | *Fold Change* | *P* | *Q* |
| --- | --- | --- | --- | --- |
| Fc receptor like B | FCRLB | -2.244 | 0.001 | 0.019 |
| histidine decarboxylase | HDC | -2.234 | 0.002 | 0.024 |
| solute carrier family 10 member 6 | SLC10A6 | -2.206 | <0.001 | 0.011 |
| immunoglobulin superfamily containing leucine rich repeat 2 | ISLR2 | -2.205 | 0.002 | 0.024 |
| TNF receptor superfamily member 17 | TNFRSF17 | -2.184 | <0.001 | 0.011 |
| DNA methyltransferase 3 beta | DNMT3B | -2.157 | 0.003 | 0.028 |
| surfactant protein D | SFTPD | -2.146 | 0.001 | 0.012 |
| C-C motif chemokine ligand 7 | CCL7 | -2.145 | 0.002 | 0.025 |
| matrix metallopeptidase 12 | MMP12 | -2.141 | 0.003 | 0.028 |
| reticulon 4 receptor like 1 | RTN4RL1 | -2.087 | <0.001 | 0.008 |
| prostaglandin-endoperoxide synthase 2 | PTGS2 | -2.079 | 0.001 | 0.018 |
| harakiri, BCL2 interacting protein | HRK | -2.058 | 0.002 | 0.025 |
| reelin | RELN | -2.042 | <0.001 | 0.009 |
| interferon regulatory factor 6 | IRF6 | -2.032 | <0.001 | 0.011 |
| distal-less homeobox 2 | DLX2 | -2.021 | 0.001 | 0.012 |
| suppressor of variegation 3-9 homolog 2 | SUV39H2 | -1.986 | <0.001 | 0.003 |
| CEA cell adhesion molecule 3 | CEACAM3 | -1.971 | 0.005 | 0.044 |
| triggering receptor expressed on myeloid cells 1 | TREM1 | -1.943 | 0.001 | 0.019 |
| cathepsin W | CTSW | -1.941 | 0.005 | 0.041 |
| phospholipase D1 | PLD1 | -1.905 | 0.004 | 0.033 |
| RAD51 paralog B | RAD51B | -1.901 | 0.003 | 0.029 |
| lymphotoxin alpha | LTA | -1.897 | 0.001 | 0.019 |
| E2F transcription factor 1 | E2F1 | -1.890 | 0.002 | 0.027 |
| C-type lectin domain containing 7A | CLEC7A | -1.832 | <0.001 | 0.008 |
| ADAM metallopeptidase with thrombospondin type 1 motif 16 | ADAMTS16 | -1.812 | 0.004 | 0.038 |
| flap structure-specific endonuclease 1 | FEN1 | -1.804 | 0.001 | 0.012 |
| suppressor of variegation 3-9 homolog 1 | SUV39H1 | -1.793 | 0.001 | 0.019 |
| family with sequence similarity 104 member A | FAM104A | -1.790 | 0.001 | 0.012 |
| SH2 domain containing 1A | SH2D1A | -1.783 | 0.006 | 0.046 |
| macrophage receptor with collagenous structure | MARCO | -1.768 | 0.004 | 0.039 |
| Fas ligand | FASLG | -1.765 | 0.004 | 0.036 |
| BLM RecQ like helicase | BLM | -1.761 | 0.003 | 0.032 |
| solute carrier family 17 member 6 | SLC17A6 | -1.761 | 0.002 | 0.020 |
| CD6 molecule | CD6 | -1.758 | 0.005 | 0.044 |
| transmembrane protein 100 | TMEM100 | -1.746 | 0.004 | 0.037 |
| granulysin | GNLY | -1.736 | 0.005 | 0.044 |
| prostaglandin E receptor 3 | PTGER3 | -1.705 | 0.002 | 0.025 |
| lysine demethylase 2B | KDM2B | -1.539 | 0.003 | 0.030 |

## Supplementary table 11: KEGG Pathways associated with differentially expressed genes from MND/ALS frozen spinal cord.

| *KEGG Symbol* | *KEGG Pathway* | *Number of Genes* | *Genes in Pathway* | | |
| --- | --- | --- | --- | --- | --- |
|  |  |  | ***Upregulated Genes*** | ***Down Regulated Genes*** |  |
| hsa04380 | Osteoclast differentiation | **12** | *PIK3CG, LILRB4, TNFRSF1A, TGFBR1, RELB, FOS, TYROBP, TREM2, FCGR2B, FCGR3A, FCGR1A, SOCS3,* |  |  |
| hsa05142 | Chagas disease | **12** | *CASP8, PIK3CG, TNFRSF1A, TGFBR1, FAS, C1QA, FOS, TLR2, C3, C1QC, C1QB, CCL2,* |  |  |
| hsa05152 | Tuberculosis | **11** | *CASP8, TNFRSF1A, CD74, TLR2, C3, FCER1G, CD14, CTSS, FCGR2B, FCGR3A, FCGR1A,* |  |  |
| hsa04668 | Tumour Necrosis Factor Signalling Pathway | **10** | *CASP8, BIRC3, PIK3CG, MMP14, TNFRSF1A, FAS, FOS, TNFRSF1B, SOCS3, CCL2,* |  |  |
| hsa05168 | Herpes Simplex virus 1 infection | **10** | *CASP8, TNFRSF1A, PILRA, CD74, FAS, FOS, TLR2, C3, SOCS3, CCL2,* |  |  |
| hsa04145 | Phagosome | **9** | *TLR2, C3, CD14, CTSS, FCGR2B, FCGR3A, FCGR1A, MSR1,* | *TUBB3* |  |
| hsa05150 | Staphylococcus aureus infection | **9** | *C3AR1, C1QA, C3, FCGR2B, C1QC, FPR1, FCGR3A, C1QB, FCGR1A,* |  |  |
| hsa05161 | Hepatitis B virus | **9** | *CASP8, PIK3CG, DDB2, TGFBR1, FAS, MYC, FOS, TLR2, CDKN1A,* |  |  |
| hsa05200 | Pathways in cancer | **9** | *CASP8, BIRC3, PIK3CG, TGFBR1, FAS, MYC, FOS, CDKN1A,* | *EGFR* |  |
| hsa04010 | MAPK signalling pathway | **8** | *TNFRSF1A, TGFBR1, FAS, RELB, MYC, FOS, CD14,* | *EGFR* |  |
| hsa04060 | Cytokine-Cytokine Receptor Interaction | **8** | *OSMR, TNFSF13B, TNFRSF1A, TGFBR1, FAS, TNFRSF1B, CCL2,* | *EGFR* |  |
| hsa04666 | Fc gamma R-mediated phagocytosis | **8** | *WAS, PIK3CG, DOCK2, VAV1, LYN, FCGR1A, FCGR3A, FCGR2B,* |  |  |
| hsa05133 | Pertussis | **8** | *NOD1, FOS, CASP1, CD14, C3, C1QA, C1QC, C1QB,* |  |  |
| hsa04064 | NF-kappa B signalling pathway | **7** | *BIRC3, TNFSF13B, TNFRSF1A, RELB, LYN, BCL2A1, CD14,* |  |  |
| hsa04650 | Natural killer cell mediated cytotoxicity | **7** | *PIK3CG, PTPN6, FAS, VAV1, FCER1G, TYROBP, FCGR3A,* |  |  |

## Supplementary table 12: Genes Associated with More Rapid Disease Progression from Frozen Spinal cord.

| *Full Gene Name* | *Official Gene Symbol* | *Mean Survival (Months)* | | *Cox Proportional Hazards coefficient* | *Log Rank P=* |
| --- | --- | --- | --- | --- | --- |
|  |  | ***Low Expressing Cases*** | ***High Expressing Cases*** |  |  |
| tubulin beta 4A class IVa | *TUBB4A* | 55.75 | 19.75 | 2.36 | <0.001 |
| potassium inwardly rectifying channel subfamily J member 10 | *KCNJ10* | 54.75 | 20.75 | 1.87 | <0.001 |
| epidermal growth factor receptor | *EGFR* | 54.875 | 20.625 | 1.54 | 0.01 |
| myelin associated oligodendrocyte basic protein | *MOBP* | 55.75 | 19.75 | 1.44 | 0.01 |
| fatty acid 2-hydroxylase | *FA2H* | 54.875 | 20.625 | 1.41 | 0.01 |
| protein kinase C theta | *PRKCQ* | 52.5 | 23 | 1.37 | 0.02 |
| 2',3'-cyclic nucleotide 3' phosphodiesterase | *CNP* | 53.625 | 21.875 | 1.36 | 0.02 |
| cytochrome P450 family 27 subfamily A member 1 | *CYP27A1* | 53.625 | 21.875 | 1.36 | 0.02 |
| endothelial cell adhesion molecule | *ESAM* | 51.5 | 24 | 1.33 | 0.03 |
| ring finger protein 8 | *RNF8* | 52.375 | 23.125 | 1.28 | 0.03 |
| inhibitor of nuclear factor kappa B kinase regulatory subunit gamma | *IKBKG* | 54.25 | 21.25 | 1.28 | 0.02 |
| peroxiredoxin 1 | *PRDX1* | 54.25 | 21.25 | 1.28 | 0.02 |
| Rac family small GTPase 1 | *RAC1* | 54.25 | 21.25 | 1.28 | 0.02 |
| peptidyl arginine deiminase 2 | *PADI2* | 53.375 | 22.125 | 1.25 | 0.02 |
| cyclin dependent kinase inhibitor 1C | *CDKN1C* | 54.75 | 20.75 | 1.22 | 0.02 |
| colony stimulating factor 2 receptor subunit beta | *CSF2RB* | 49.75 | 25.75 | 1.21 | 0.04 |
| proton activated chloride channel 1 | *TMEM206* | 53.25 | 22.25 | 1.17 | 0.03 |
| NACHT and WD repeat domain containing 1 | *NWD1* | 52.625 | 22.875 | 1.14 | 0.04 |
| cyclin I | *CCNI* | 53 | 22.5 | 1.14 | 0.04 |
| integrin subunit alpha V | *ITGAV* | 53 | 22.5 | 1.14 | 0.04 |
| erb-b2 receptor tyrosine kinase 3 | *ERBB3* | 50.625 | 24.875 | 1.12 | 0.05 |
| SPT7 like, STAGA complex subunit gamma | *SUPT7L* | 53.5 | 22 | 1.09 | 0.04 |
| IQ motif and Sec7 domain ArfGEF 1 | *IQSEC1* | 53.25 | 22.25 | 1.08 | 0.04 |

## Supplementary table 13: Genes Associated with Slower Disease Progression from Frozen Spinal cord

| *Full Gene Name* | *Official Gene Symbol* | *Mean Survival (Months)* | | *Cox Proportional Hazards coefficient* | *Log Rank P=* |
| --- | --- | --- | --- | --- | --- |
|  |  | ***Low Expressing Cases*** | ***High Expressing Cases*** |  |  |
| interleukin 2 receptor subunit gamma | *IL2RG* | 16.625 | 58.875 | -2.65 | <0.001 |
| serine peptidase inhibitor, Kunitz type 1 | *SPINT1* | 17.875 | 57.625 | -2.43 | <0.001 |
| DNA methyltransferase 1 | *DNMT1* | 20.875 | 54.625 | -2.05 | <0.001 |
| autophagy related 5 | *ATG5* | 23.875 | 51.625 | -1.88 | 0.01 |
| Cluster of Differentiation 163 | *CD163* | 20.75 | 54.75 | -1.87 | <0.001 |
| T-cell surface glycoprotein CD3 delta chain | *CD3D* | 20 | 55.5 | -1.79 | <0.001 |
| TNF receptor superfamily member 1A | *TNFRSF1A* | 23.125 | 52.375 | -1.58 | 0.01 |
| complement C1q A chain | *C1QA* | 23.375 | 52.125 | -1.56 | 0.02 |
| checkpoint kinase 2 | *CHEK2* | 20.25 | 55.25 | -1.52 | 0.01 |
| SRY-box transcription factor 4 | *SOX4* | 21.5 | 54 | -1.51 | 0.01 |
| serpin family A member 3 | *SERPINA3* | 25.25 | 50.25 | -1.49 | 0.02 |
| non-SMC condensin I complex subunit H | *NCAPH* | 21.875 | 53.625 | -1.46 | 0.01 |
| complement C1q B chain | *C1QB* | 24.5 | 51 | -1.45 | 0.02 |
| ceruloplasmin | *CP* | 24.5 | 51 | -1.45 | 0.02 |
| FA complementation group C | *FANCC* | 23 | 52.5 | -1.38 | 0.02 |
| neuroligin 2 | *NLGN2* | 26.25 | 49.25 | -1.34 | 0.04 |
| BH3 interacting domain death agonist | *BID* | 24.875 | 50.625 | -1.31 | 0.03 |
| killer cell lectin like receptor K1 | *KLRK1* | 23.125 | 52.375 | -1.28 | 0.03 |
| C-X-C motif chemokine ligand 9 | *CXCL9* | 21.25 | 54.25 | -1.28 | 0.02 |
| calcium/calmodulin dependent protein kinase IV | *CAMK4* | 22.125 | 53.375 | -1.25 | 0.02 |
| membrane spanning 4-domains A1 | *MS4A1* | 21.375 | 54.125 | -1.24 | 0.03 |
| nuclear factor kappa B subunit 1 | *NFKB1* | 20.75 | 54.75 | -1.22 | 0.02 |
| S100 calcium binding protein A12 | *S100A12* | 22.375 | 53.125 | -1.14 | 0.04 |

## Supplementary table 14: Genes Associated with more rapid disease progression from FFPE spinal cord

| *Full Gene Name* | *Official Gene Symbol* | *Mean Survival (Months)* | | *Cox Proportional Hazards coefficient* | *Log Rank P=* |
| --- | --- | --- | --- | --- | --- |
|  |  | ***Low Expression Cases*** | ***High Expression Cases*** |  |  |
| TNF Receptor Superfamily Member 17 | *TNFRSF17* | 59.143 | 18.75 | 2.259 | 0.001 |
| Inhibitor of Nuclear Factor Kappa B Kinase Subunit Epsilon | *IKBKE* | 58.857 | 19 | 1.881 | 0.003 |
| Killer Cell Lectin Like Receptor D1 | *KLRD1* | 57.714 | 20 | 1.824 | 0.004 |
| Proliferating Cell Nuclear Antigen | *PCNA* | 49.857 | 26.875 | 1.682 | 0.026 |
| Mitogen-Activated Protein Kinase Kinase Kinase 1 | *MAP3K1* | 57.571 | 20.125 | 1.675 | 0.005 |
| Interleukin 1 Receptor Associated Kinase 2 | *IRAK2* | 56.857 | 20.75 | 1.621 | 0.01 |
| Embryonic Ectoderm Development | *EED* | 54.571 | 22.75 | 1.605 | 0.013 |
| Sialic Acid Binding Ig Like Lectin 8 | *SIGLEC8* | 54.429 | 22.875 | 1.591 | 0.013 |
| Serpin Family F Member 1 | *SERPINF1* | 53.286 | 23.875 | 1.524 | 0.02 |
| DNA Ligase 1 | *LIG1* | 53.143 | 24 | 1.521 | 0.019 |
| Leucine Rich Alpha-2-Glycoprotein 1 | *LRG1* | 53.429 | 23.75 | 1.436 | 0.026 |
| Non-SMC Condensin I Complex Subunit H | *NCAPH* | 54.714 | 22.625 | 1.365 | 0.02 |
| PTPRF Interacting Protein Alpha 4 | *PPFIA4* | 54.714 | 22.625 | 1.365 | 0.02 |
| Lysyl Oxidase | *LOX* | 52.286 | 24.75 | 1.332 | 0.025 |
| Intercellular Adhesion Molecule 2 | *ICAM2* | 50.286 | 26.5 | 1.302 | 0.048 |
| Transglutaminase 1 | *TGM1* | 54.429 | 22.875 | 1.275 | 0.03 |
| DNA Methyltransferase 3 Beta | *DNMT3B* | 52.714 | 24.375 | 1.273 | 0.032 |
| Solute Carrier Family 2 Member 5 | *SLC2A5* | 53.571 | 23.625 | 1.222 | 0.037 |
| Nucleotide Binding Oligomerization Domain Containing 1 | *NOD1* | 50.857 | 26 | 1.203 | 0.043 |
| Transmembrane Protein 204 | *TMEM204* | 50.857 | 26 | 1.203 | 0.043 |
| Eomesodermin | *EOMES* | 53 | 24.125 | 1.166 | 0.048 |

## Supplementary table 15: Genes Associated with slower disease progression from FFPE spinal cord.

| *Full Gene Name* | *Official Gene Symbol* | *Mean Survival (Months)* | | *Cox Proportional Hazards coefficient* | *Log Rank P=* |
| --- | --- | --- | --- | --- | --- |
|  |  | ***Low Expression Cases*** | ***High Expression Cases*** |  |  |
| Interferon Induced Transmembrane Protein 2 | *IFITM2* | 18.857 | 54 | -2.775 | 0.001 |
| Leukocyte Associated Immunoglobulin Like Receptor 1 | *LAIR1* | 19.857 | 53.125 | -2.16 | 0.003 |
| Interferon Regulatory Factor 3 | *IRF3* | 17.143 | 55.5 | -1.976 | 0.002 |
| BCL2 Associated Agonist of Cell Death | *BAD* | 18 | 54.75 | -1.874 | 0.003 |
| T Cell Immune Regulator 1, ATPase H+ Transporting V0 Subunit A3 | *TCIRG1* | 23.571 | 49.875 | -1.587 | 0.017 |
| Uracil DNA Glycosylase | *UNG* | 23.571 | 49.875 | -1.587 | 0.017 |
| Tumour Necrosis Factor Receptor Superfamily 1b | *TNFRSF1B* | 20.857 | 52.25 | -1.5 | 0.012 |
| Cluster of Differentiation 44 | *CD44* | 20.857 | 52.25 | -1.462 | 0.016 |
| Peroxiredoxin 1 | *PRDX1* | 20.429 | 52.625 | -1.376 | 0.015 |
| Sequestesome 1 | *SQSTM1* | 20.429 | 52.625 | -1.376 | 0.015 |
| Thioredoxin Reductase 1 | *TXNRD1* | 23 | 50.375 | -1.279 | 0.024 |
| Vimentin 1 | *VIM* | 24.143 | 49.375 | -1.19 | 0.039 |
| Inhibitor of Nuclear Factor Kappa B Kinase Regulatory Subunit Gamma | *IKBKG* | 24 | 49.5 | -1.142 | 0.048 |
| Vesicle Associated Membrane Protein 7 | *VAMP7* | 23.857 | 49.625 | -1.122 | 0.047 |
| Fc Fragment of Igg Receptor IIIa | *FCGR3A* | 21.857 | 51.375 | -1.118 | 0.044 |
| Calreticulin | *CALR* | 22.143 | 51.125 | -1.112 | 0.042 |
| DNA Mismatch Repair Protein Msh2 | *MSH2* | 22.143 | 51.125 | -1.112 | 0.042 |
