# Supplementary material for: Human amyotrophic lateral sclerosis/motor neuron disease: The disease‐associated microglial pathway is upregulated while APOE genotype governs risk and survival
Source: Brain Pathol. 2025 Jun 12;35(6):e70019. doi: 10.1111/bpa.70019 (PMC12488259; doi:10.1111/bpa.70019)
Supplement: Supplementary file 2 — Data S2. Supplementary figure. [file BPA-35-e70019-s001.pptx]

## Slide 1
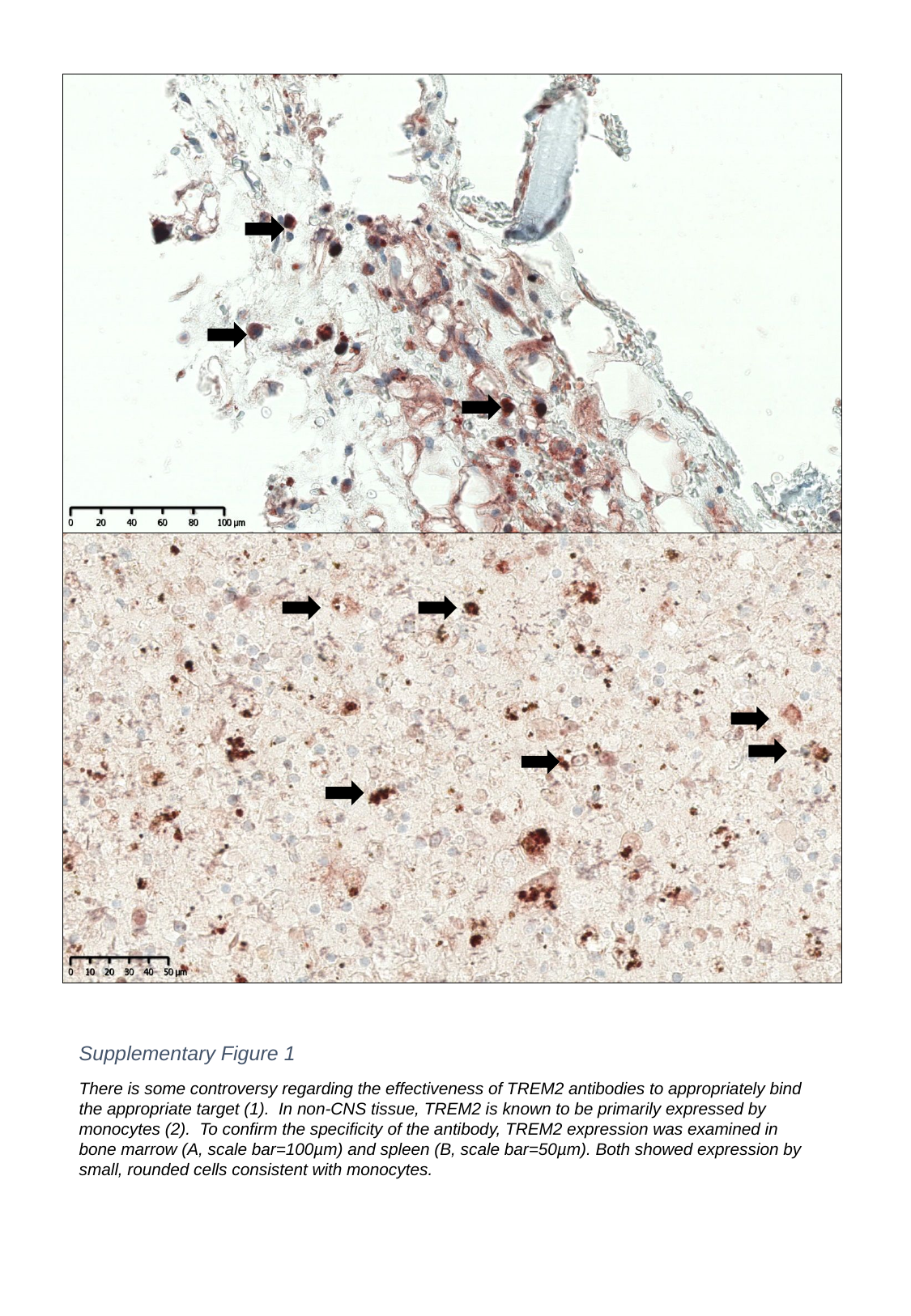

Supplementary Figure 1
There is some controversy regarding the effectiveness of TREM2 antibodies to appropriately bind the appropriate target (1). In non-CNS tissue, TREM2 is known to be primarily expressed by monocytes (2). To confirm the specificity of the antibody, TREM2 expression was examined in bone marrow (A, scale bar=100µm) and spleen (B, scale bar=50µm). Both showed expression by small, rounded cells consistent with monocytes.

## Slide 2
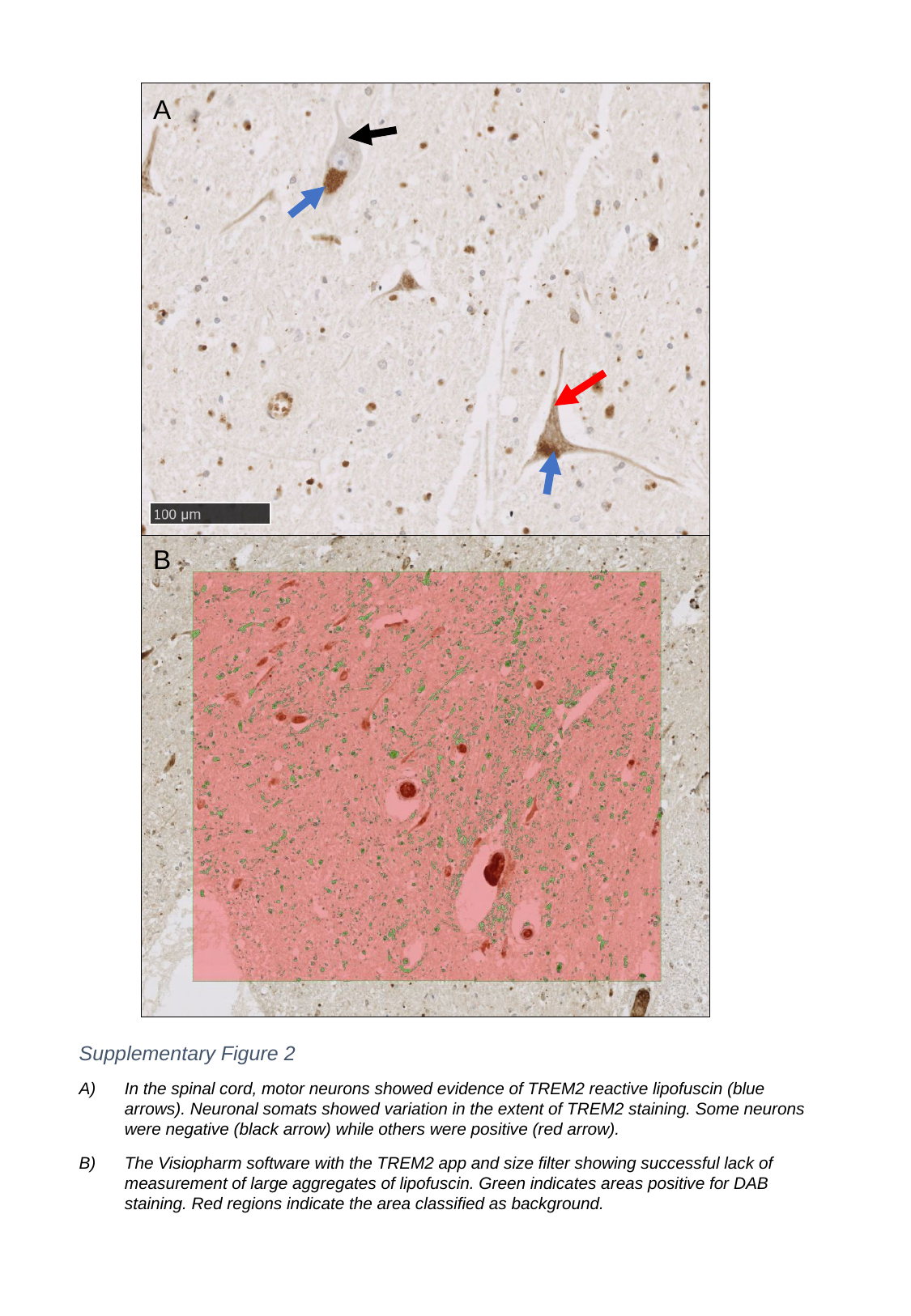

A
B
A
Supplementary Figure 2
In the spinal cord, motor neurons showed evidence of TREM2 reactive lipofuscin (blue arrows). Neuronal somats showed variation in the extent of TREM2 staining. Some neurons were negative (black arrow) while others were positive (red arrow).
The Visiopharm software with the TREM2 app and size filter showing successful lack of measurement of large aggregates of lipofuscin. Green indicates areas positive for DAB staining. Red regions indicate the area classified as background.

## Slide 3
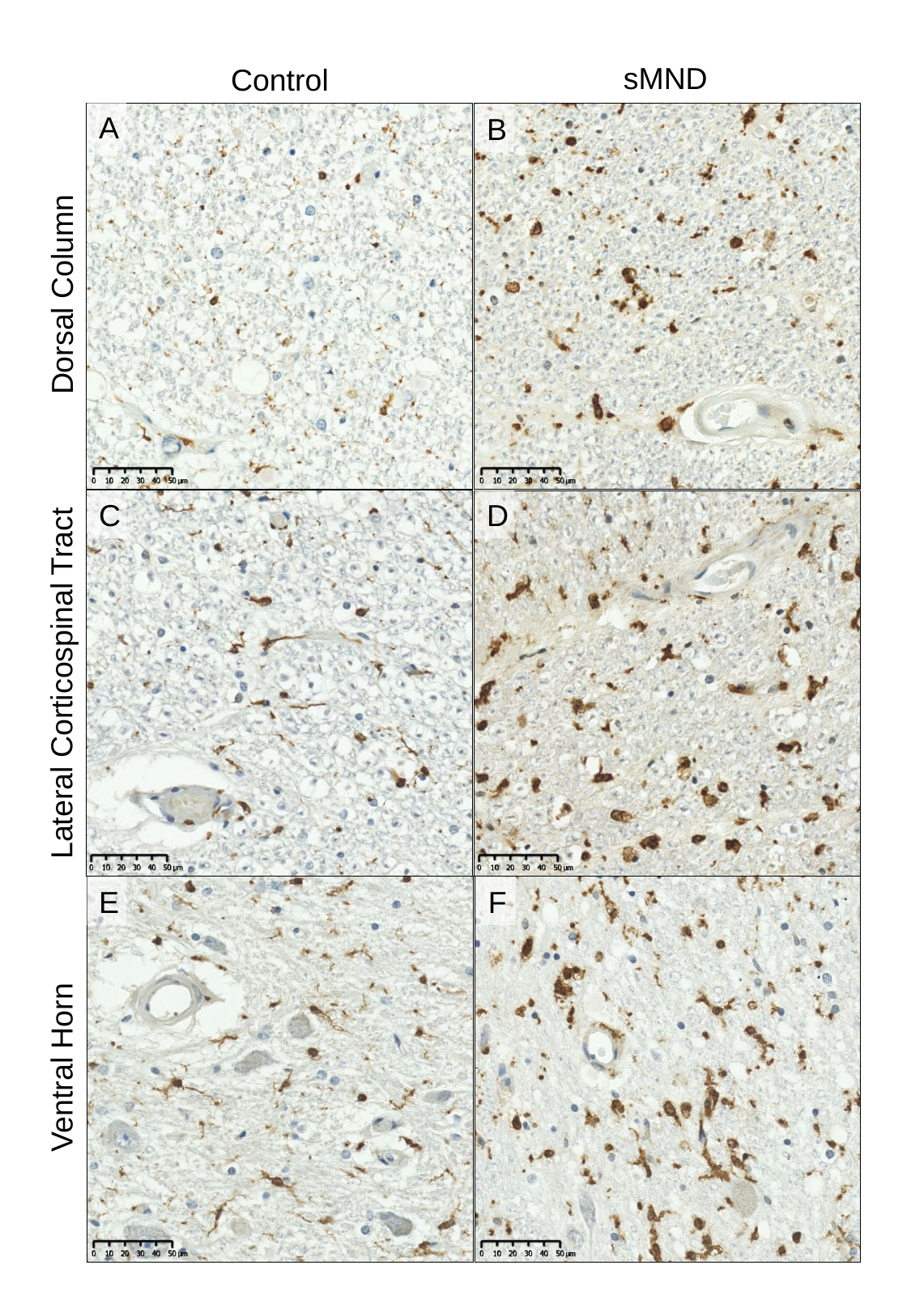

sMND
Control
A
B
Dorsal Column
D
C
Lateral Corticospinal Tract
E
F
Ventral Horn

## Slide 4
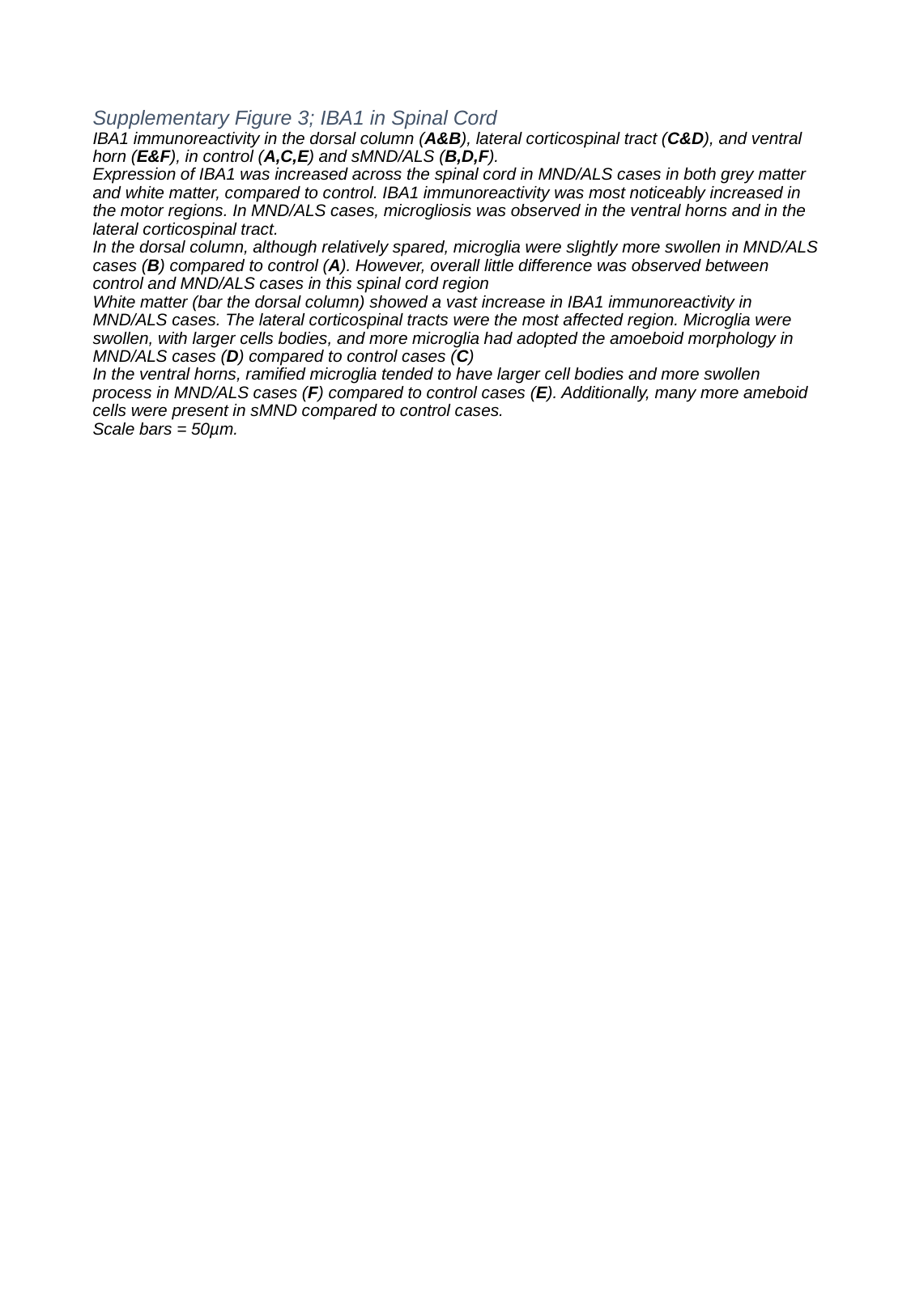

Supplementary Figure 3; IBA1 in Spinal Cord
IBA1 immunoreactivity in the dorsal column (A&B), lateral corticospinal tract (C&D), and ventral horn (E&F), in control (A,C,E) and sMND/ALS (B,D,F).
Expression of IBA1 was increased across the spinal cord in MND/ALS cases in both grey matter and white matter, compared to control. IBA1 immunoreactivity was most noticeably increased in the motor regions. In MND/ALS cases, microgliosis was observed in the ventral horns and in the lateral corticospinal tract.
In the dorsal column, although relatively spared, microglia were slightly more swollen in MND/ALS cases (B) compared to control (A). However, overall little difference was observed between control and MND/ALS cases in this spinal cord region
White matter (bar the dorsal column) showed a vast increase in IBA1 immunoreactivity in MND/ALS cases. The lateral corticospinal tracts were the most affected region. Microglia were swollen, with larger cells bodies, and more microglia had adopted the amoeboid morphology in MND/ALS cases (D) compared to control cases (C)
In the ventral horns, ramified microglia tended to have larger cell bodies and more swollen process in MND/ALS cases (F) compared to control cases (E). Additionally, many more ameboid cells were present in sMND compared to control cases.
Scale bars = 50µm.

## Slide 5
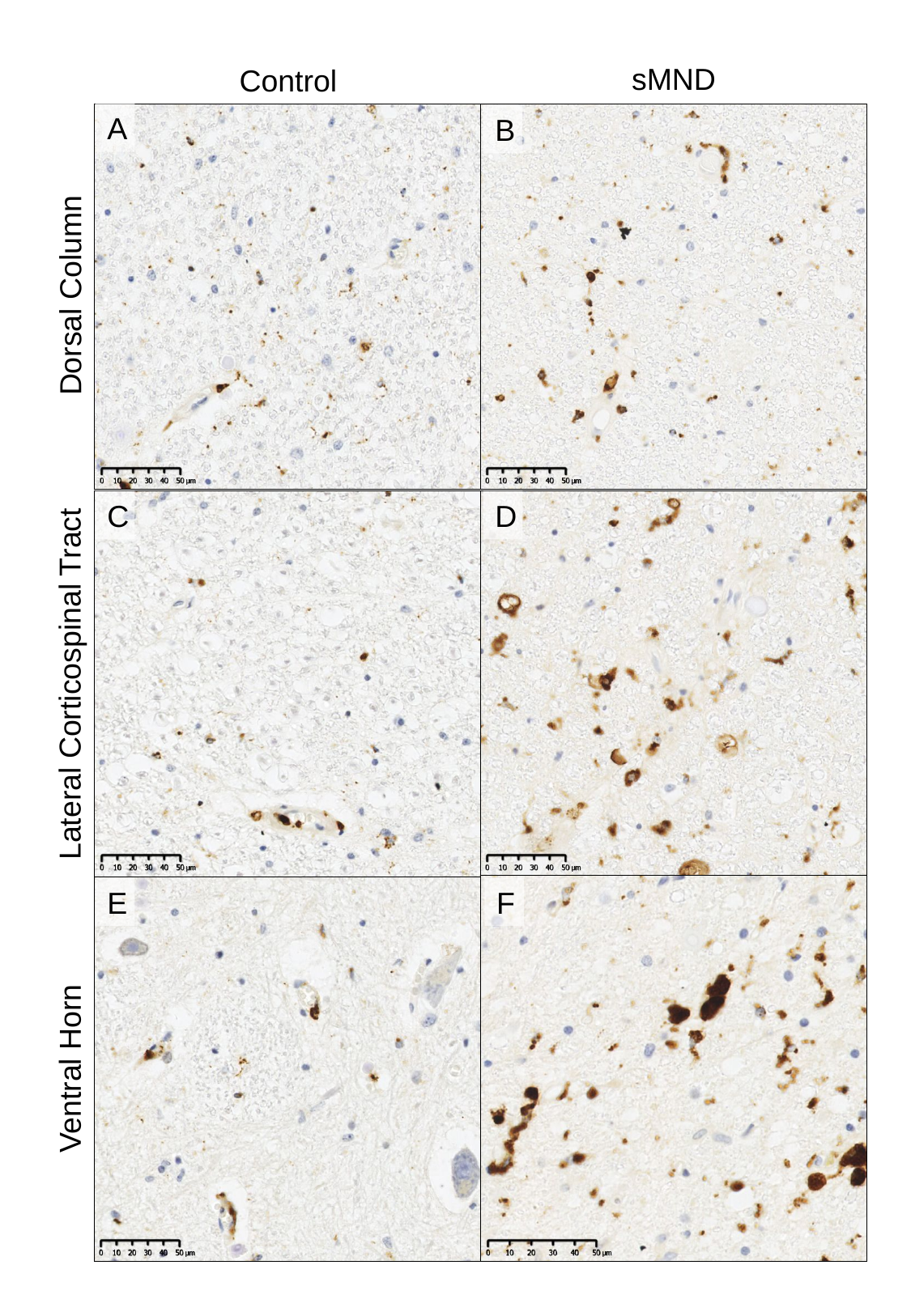

sMND
Control
A
B
Dorsal Column
D
C
Lateral Corticospinal Tract
E
F
Ventral Horn

## Slide 6
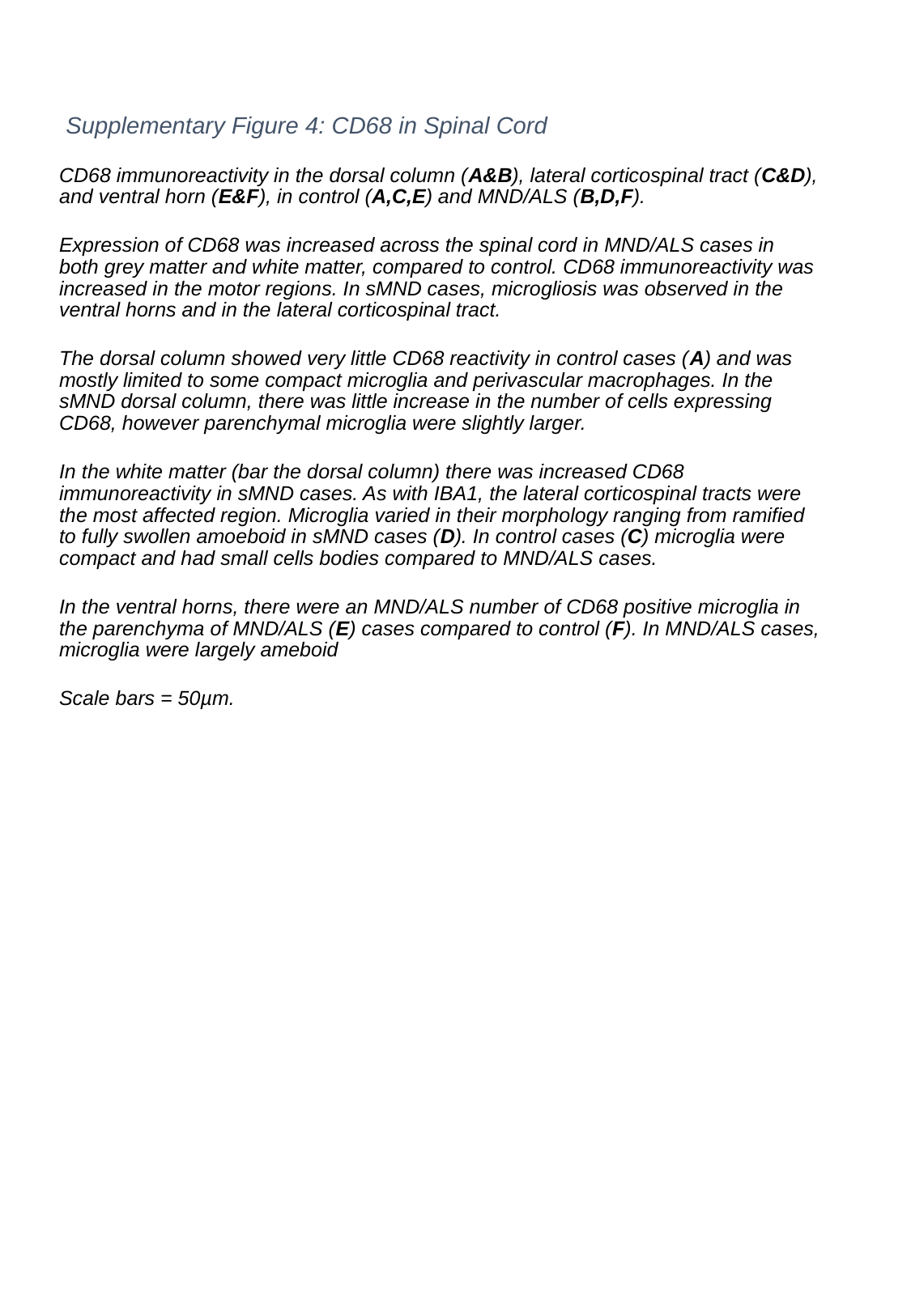

Supplementary Figure 4: CD68 in Spinal Cord
CD68 immunoreactivity in the dorsal column (A&B), lateral corticospinal tract (C&D), and ventral horn (E&F), in control (A,C,E) and MND/ALS (B,D,F).
Expression of CD68 was increased across the spinal cord in MND/ALS cases in both grey matter and white matter, compared to control. CD68 immunoreactivity was increased in the motor regions. In sMND cases, microgliosis was observed in the ventral horns and in the lateral corticospinal tract.
The dorsal column showed very little CD68 reactivity in control cases (A) and was mostly limited to some compact microglia and perivascular macrophages. In the sMND dorsal column, there was little increase in the number of cells expressing CD68, however parenchymal microglia were slightly larger.
In the white matter (bar the dorsal column) there was increased CD68 immunoreactivity in sMND cases. As with IBA1, the lateral corticospinal tracts were the most affected region. Microglia varied in their morphology ranging from ramified to fully swollen amoeboid in sMND cases (D). In control cases (C) microglia were compact and had small cells bodies compared to MND/ALS cases.
In the ventral horns, there were an MND/ALS number of CD68 positive microglia in the parenchyma of MND/ALS (E) cases compared to control (F). In MND/ALS cases, microglia were largely ameboid
Scale bars = 50µm.

## Slide 7
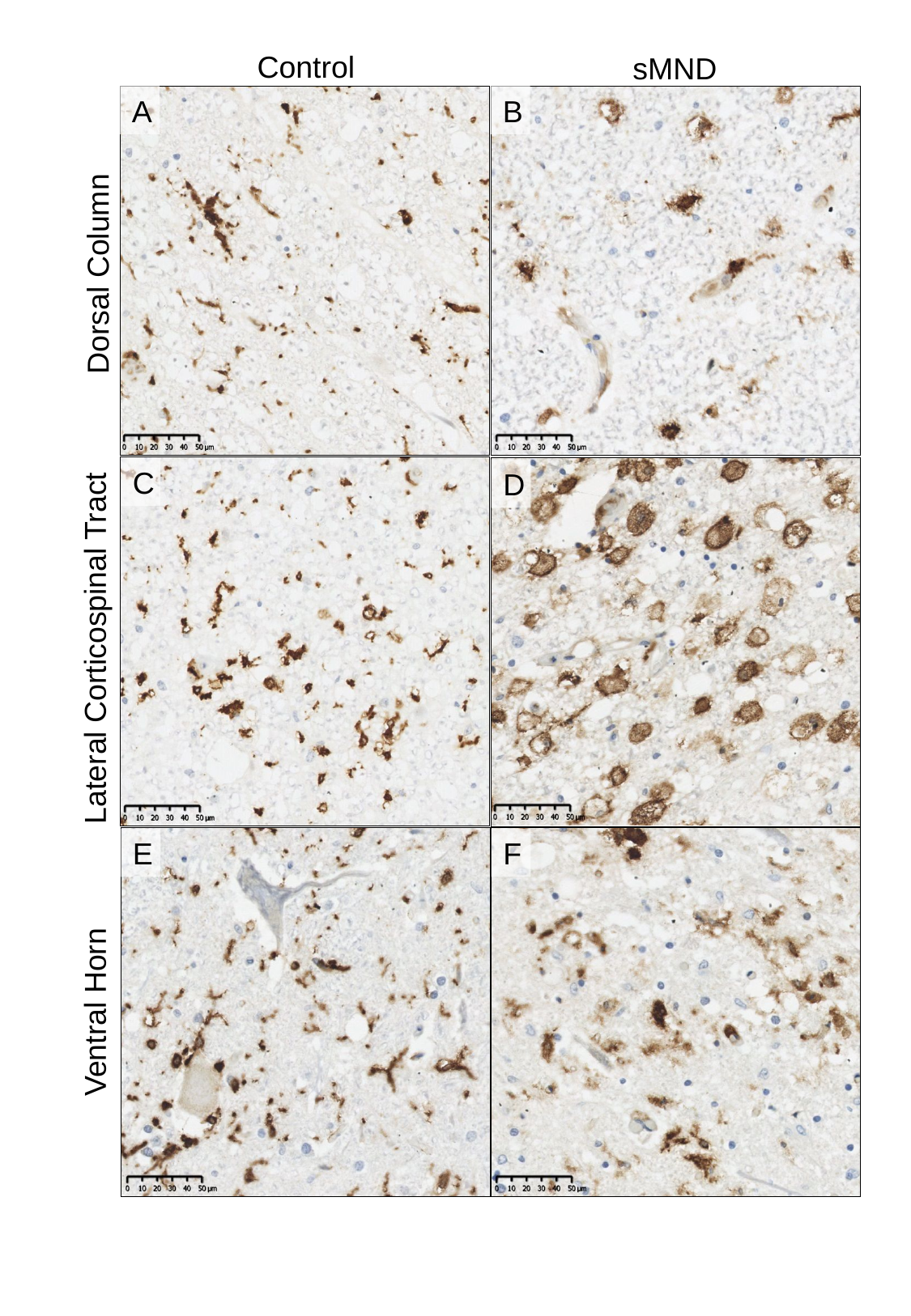

Control
sMND
A
B
Dorsal Column
C
D
Lateral Corticospinal Tract
E
F
Ventral Horn

## Slide 8
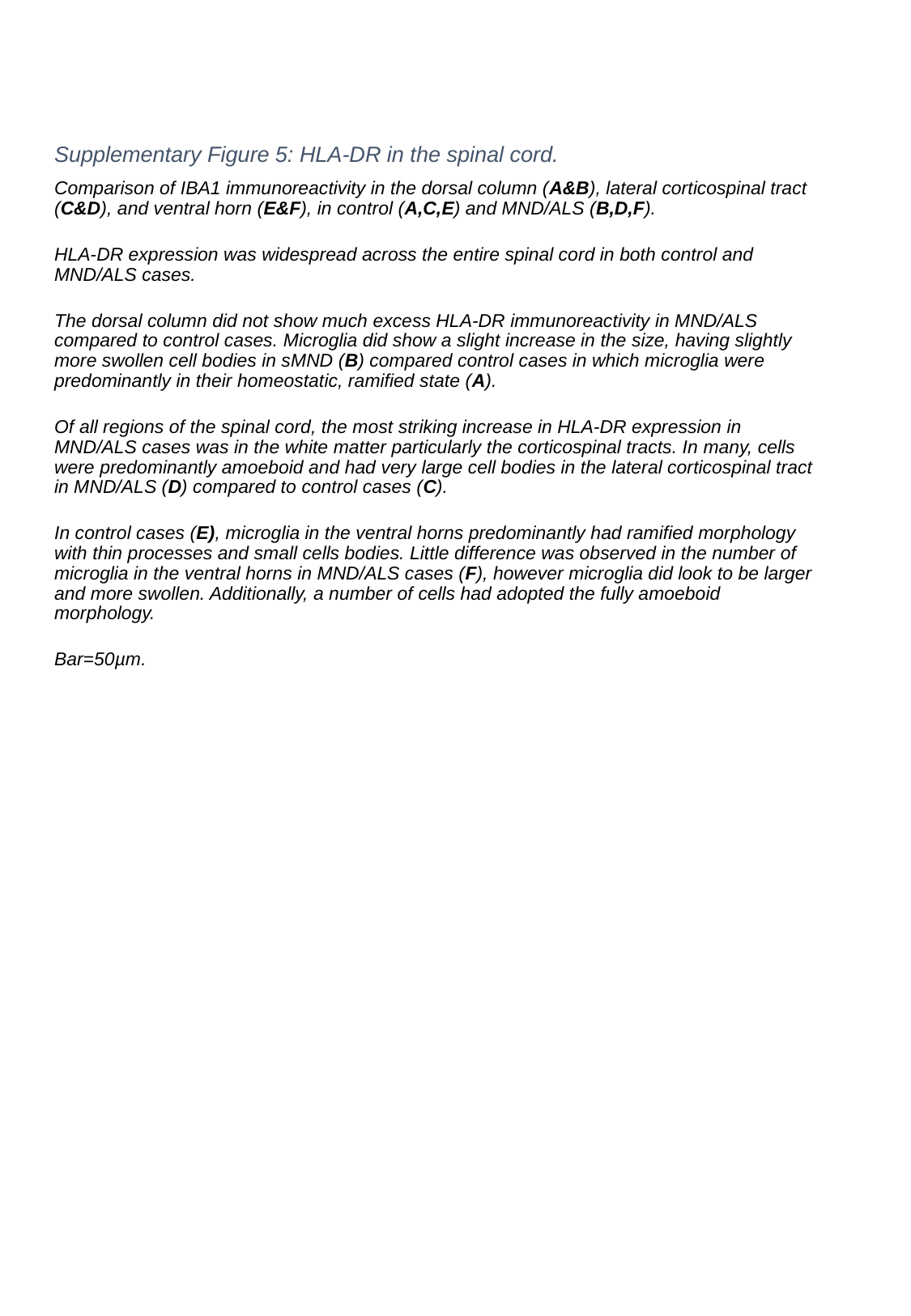

Supplementary Figure ‎5: HLA-DR in the spinal cord.
Comparison of IBA1 immunoreactivity in the dorsal column (A&B), lateral corticospinal tract (C&D), and ventral horn (E&F), in control (A,C,E) and MND/ALS (B,D,F).
HLA-DR expression was widespread across the entire spinal cord in both control and MND/ALS cases.
The dorsal column did not show much excess HLA-DR immunoreactivity in MND/ALS compared to control cases. Microglia did show a slight increase in the size, having slightly more swollen cell bodies in sMND (B) compared control cases in which microglia were predominantly in their homeostatic, ramified state (A).
Of all regions of the spinal cord, the most striking increase in HLA-DR expression in MND/ALS cases was in the white matter particularly the corticospinal tracts. In many, cells were predominantly amoeboid and had very large cell bodies in the lateral corticospinal tract in MND/ALS (D) compared to control cases (C).
In control cases (E), microglia in the ventral horns predominantly had ramified morphology with thin processes and small cells bodies. Little difference was observed in the number of microglia in the ventral horns in MND/ALS cases (F), however microglia did look to be larger and more swollen. Additionally, a number of cells had adopted the fully amoeboid morphology.
Bar=50µm.

## Slide 9
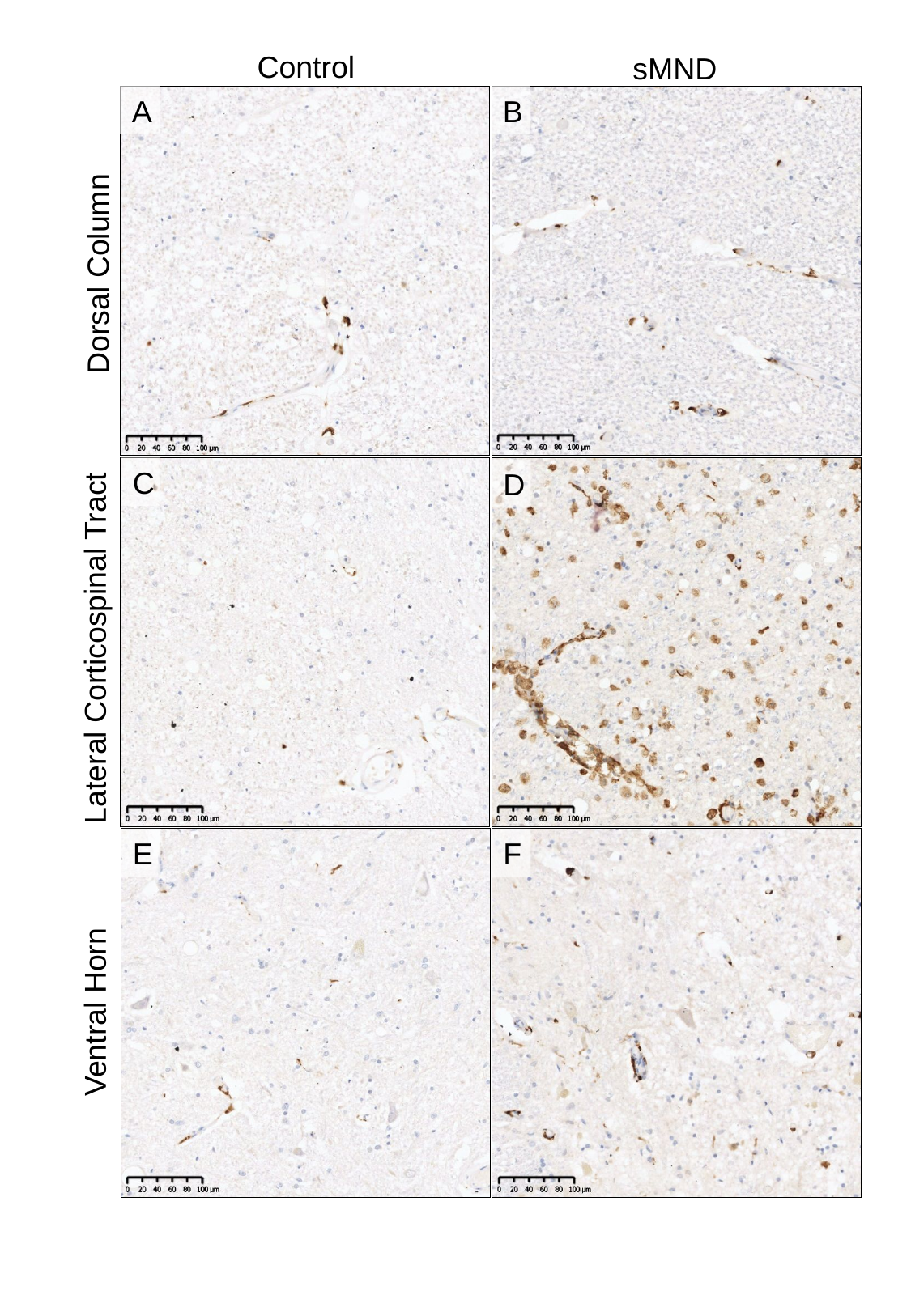

Control
sMND
A
B
Dorsal Column
C
D
Lateral Corticospinal Tract
E
F
Ventral Horn

## Slide 10
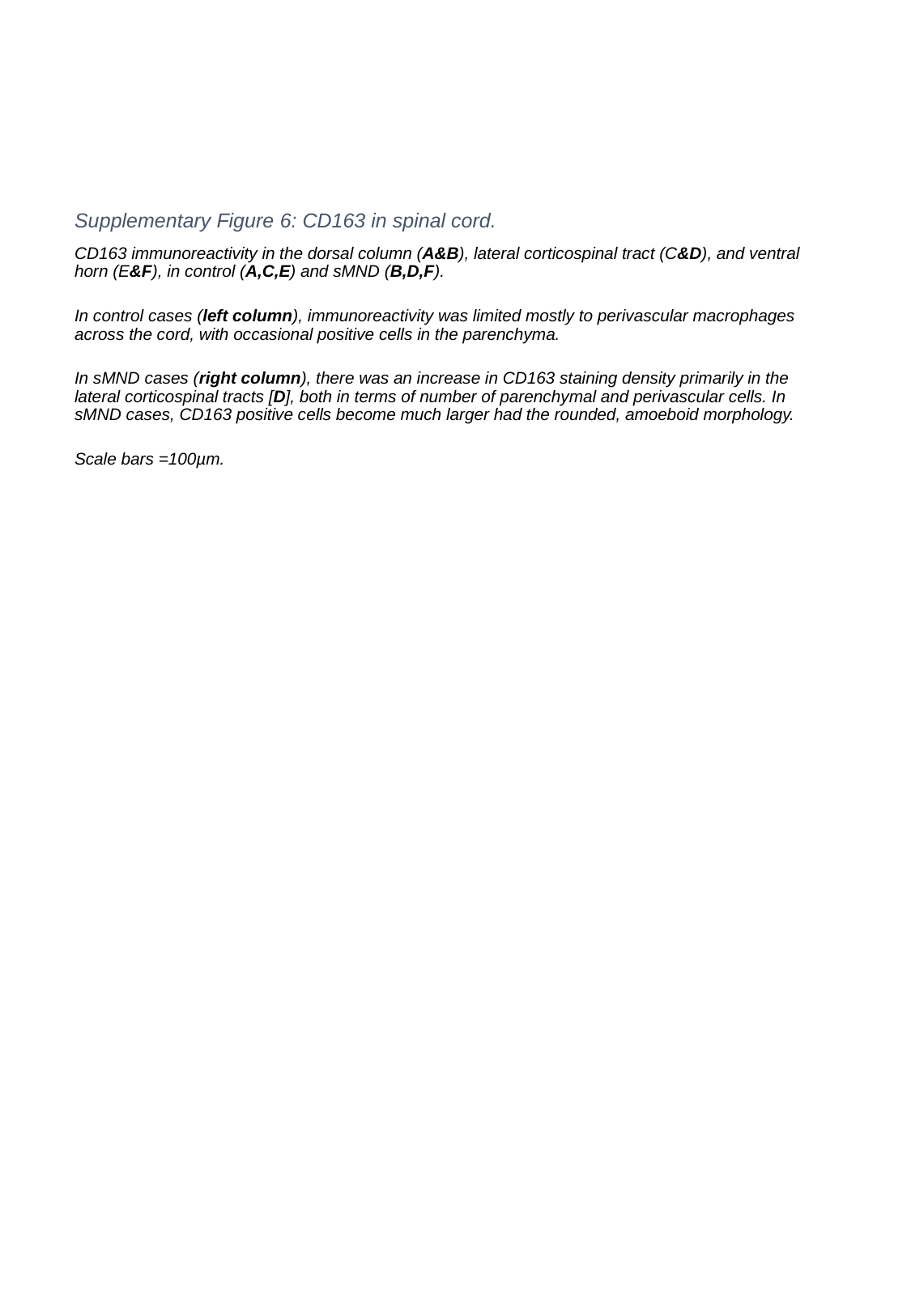

Supplementary Figure ‎6: CD163 in spinal cord.
CD163 immunoreactivity in the dorsal column (A&B), lateral corticospinal tract (C&D), and ventral horn (E&F), in control (A,C,E) and sMND (B,D,F).
In control cases (left column), immunoreactivity was limited mostly to perivascular macrophages across the cord, with occasional positive cells in the parenchyma.
In sMND cases (right column), there was an increase in CD163 staining density primarily in the lateral corticospinal tracts [D], both in terms of number of parenchymal and perivascular cells. In sMND cases, CD163 positive cells become much larger had the rounded, amoeboid morphology.
Scale bars =100µm.

## Slide 11
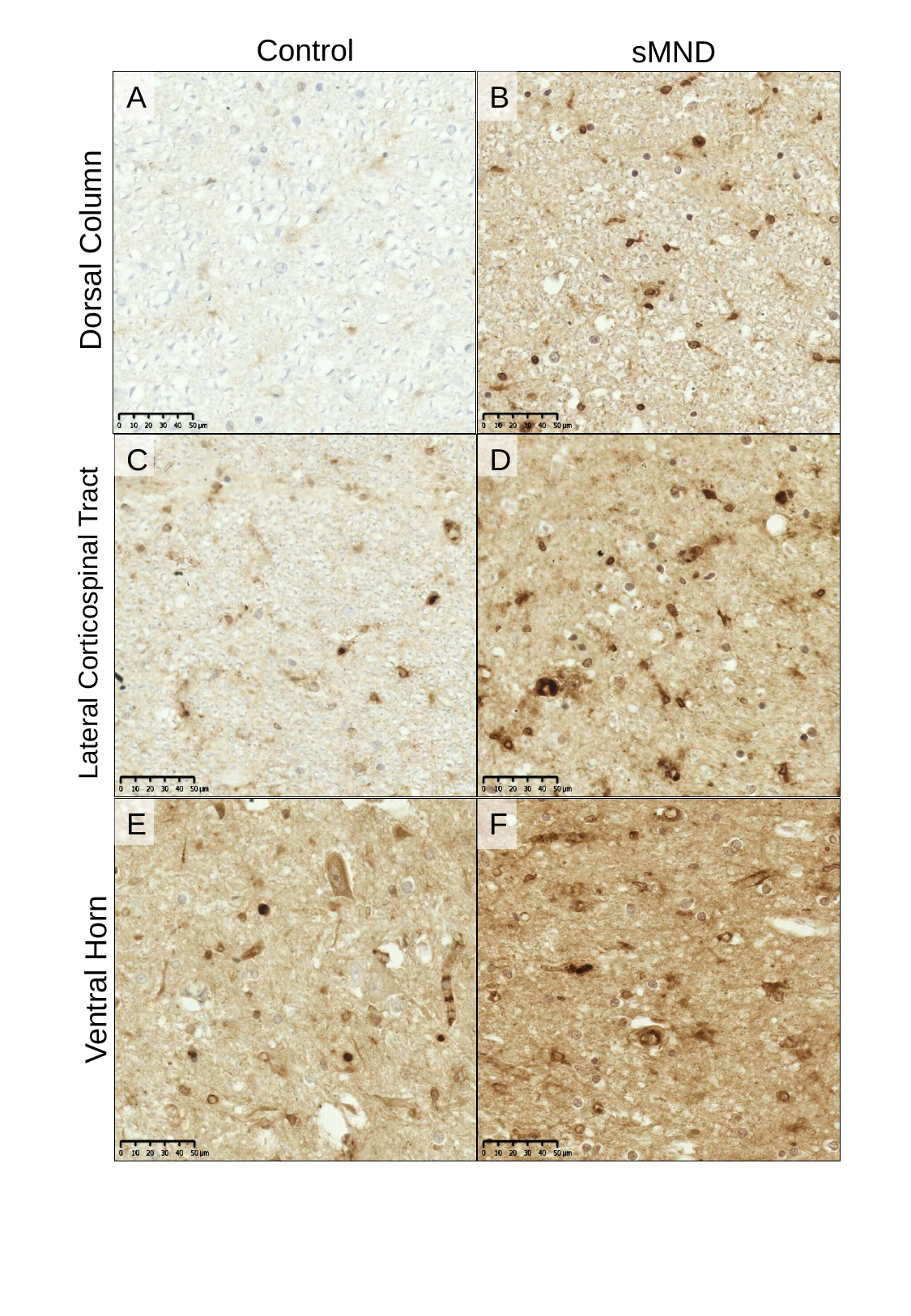

Control
sMND
B
A
Dorsal Column
C
D
Lateral Corticospinal Tract
E
F
Ventral Horn

## Slide 12
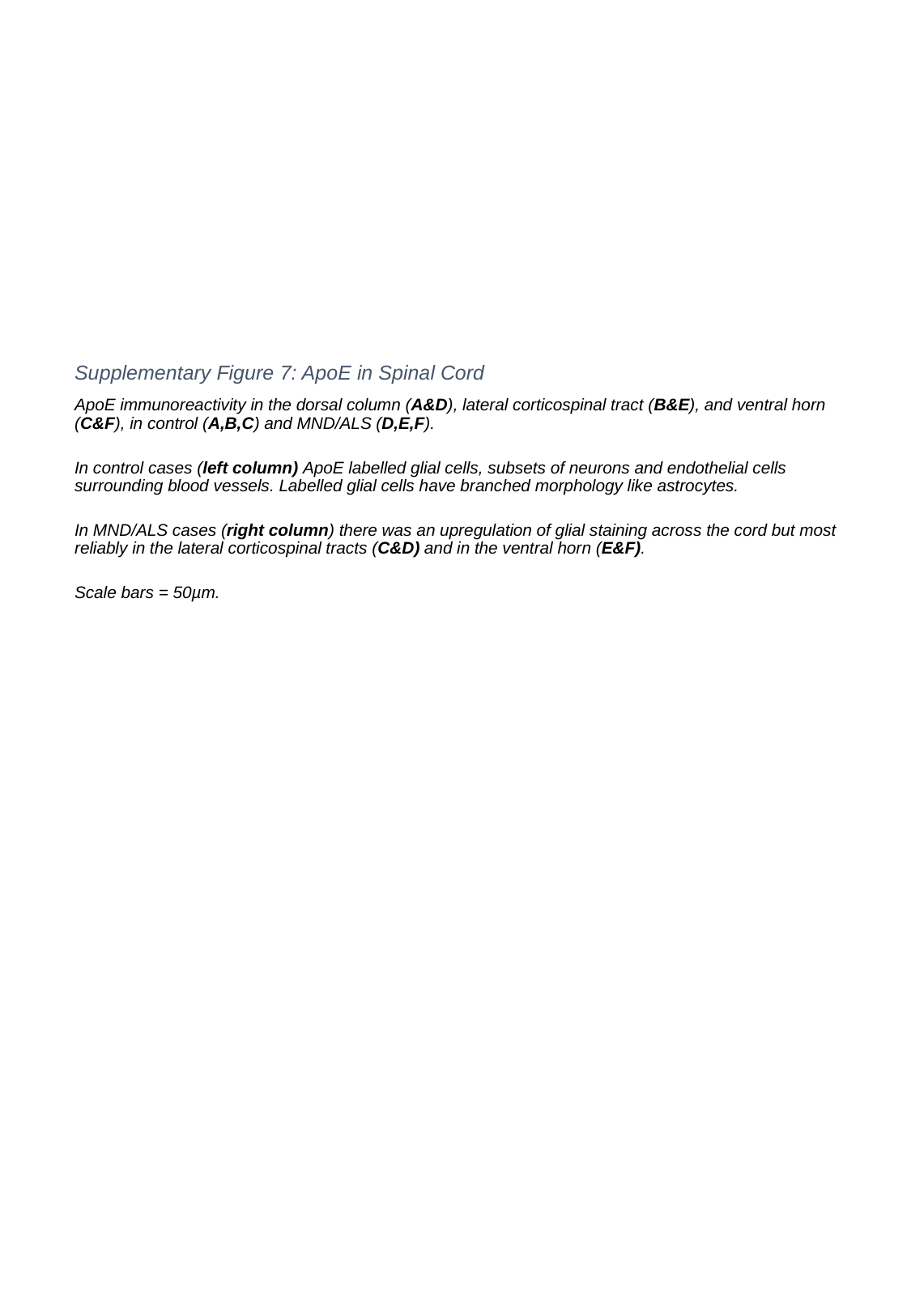

Supplementary Figure ‎7: ApoE in Spinal Cord
ApoE immunoreactivity in the dorsal column (A&D), lateral corticospinal tract (B&E), and ventral horn (C&F), in control (A,B,C) and MND/ALS (D,E,F).
In control cases (left column) ApoE labelled glial cells, subsets of neurons and endothelial cells surrounding blood vessels. Labelled glial cells have branched morphology like astrocytes.
In MND/ALS cases (right column) there was an upregulation of glial staining across the cord but most reliably in the lateral corticospinal tracts (C&D) and in the ventral horn (E&F).
Scale bars = 50µm.

## Slide 13
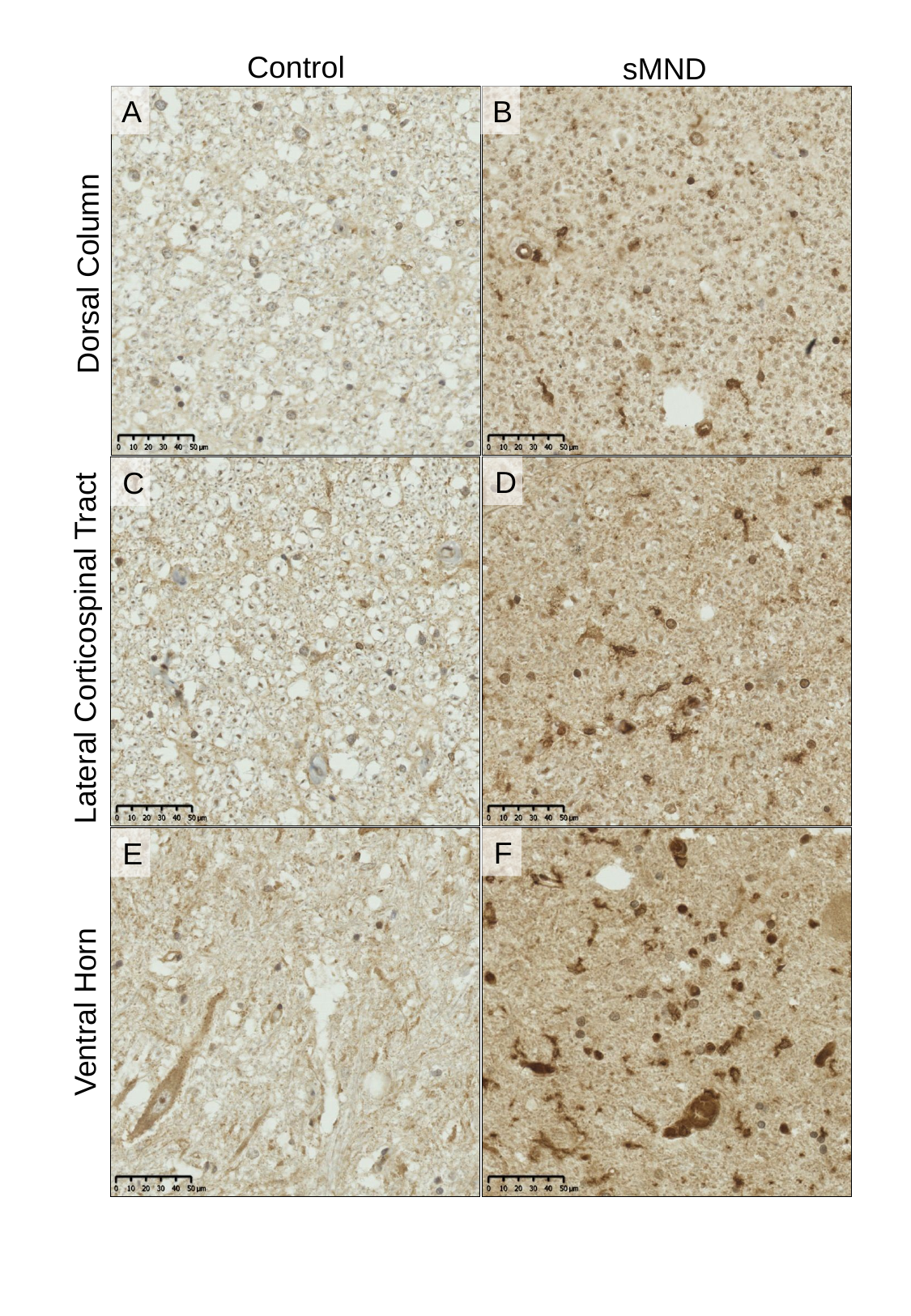

Control
sMND
A
B
Dorsal Column
D
C
Lateral Corticospinal Tract
F
E
Ventral Horn

## Slide 14
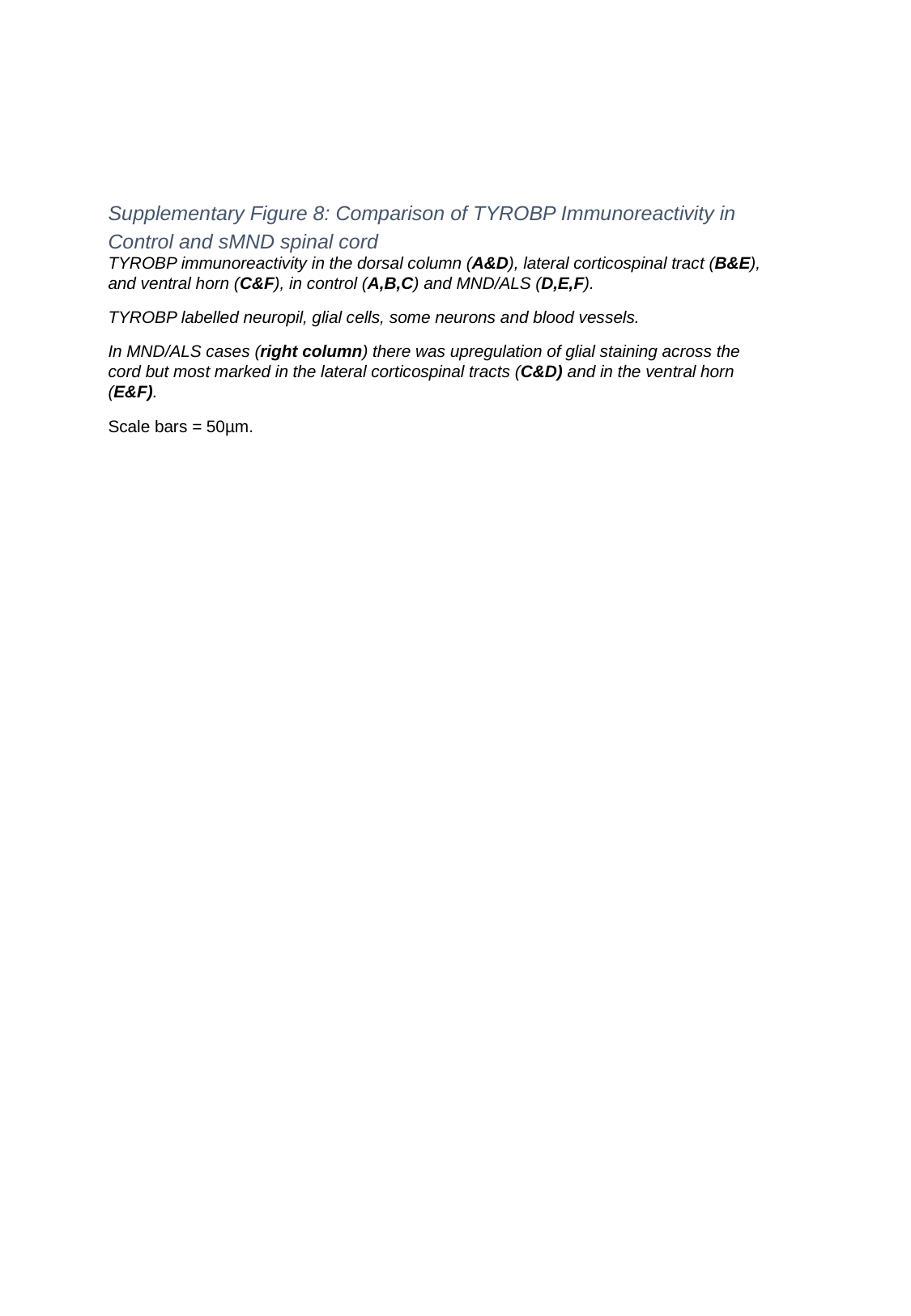

Supplementary Figure 8: Comparison of TYROBP Immunoreactivity in Control and sMND spinal cord
TYROBP immunoreactivity in the dorsal column (A&D), lateral corticospinal tract (B&E), and ventral horn (C&F), in control (A,B,C) and MND/ALS (D,E,F).
TYROBP labelled neuropil, glial cells, some neurons and blood vessels.
In MND/ALS cases (right column) there was upregulation of glial staining across the cord but most marked in the lateral corticospinal tracts (C&D) and in the ventral horn (E&F).
Scale bars = 50µm.

## Slide 15
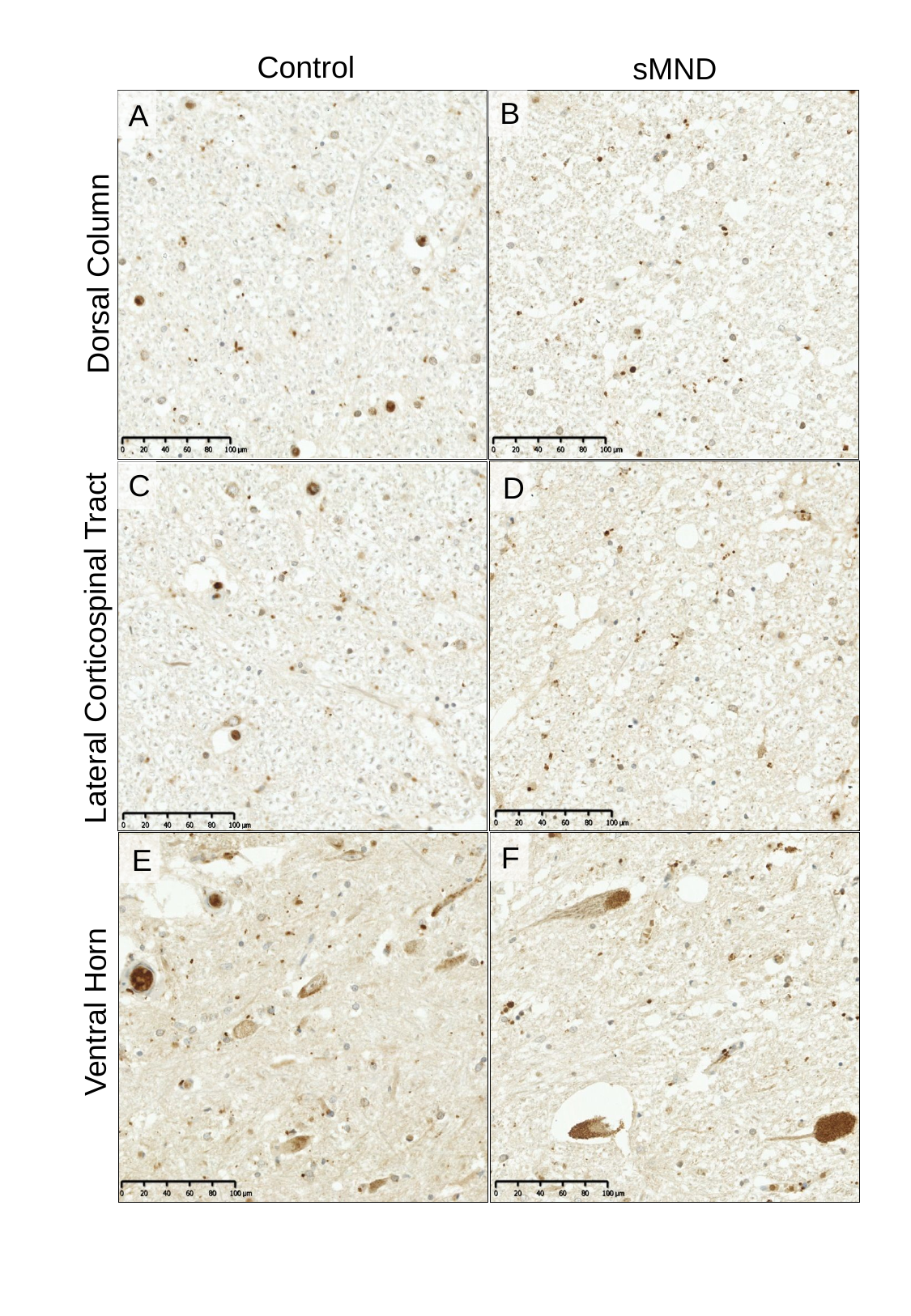

Control
sMND
B
A
Dorsal Column
C
D
Lateral Corticospinal Tract
F
E
Ventral Horn

## Slide 16
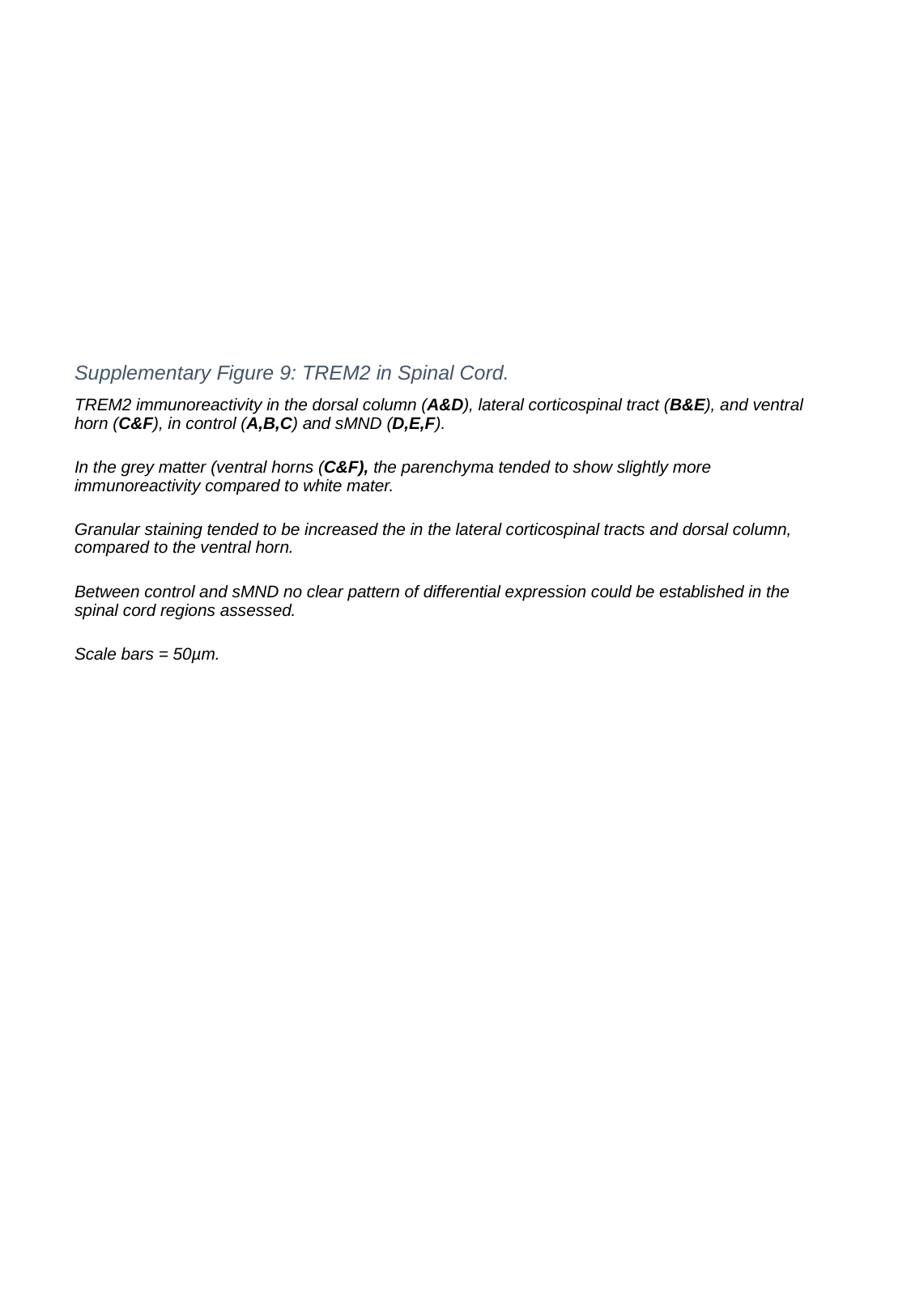

Supplementary Figure 9: TREM2 in Spinal Cord.
TREM2 immunoreactivity in the dorsal column (A&D), lateral corticospinal tract (B&E), and ventral horn (C&F), in control (A,B,C) and sMND (D,E,F).
In the grey matter (ventral horns (C&F), the parenchyma tended to show slightly more immunoreactivity compared to white mater.
Granular staining tended to be increased the in the lateral corticospinal tracts and dorsal column, compared to the ventral horn.
Between control and sMND no clear pattern of differential expression could be established in the spinal cord regions assessed.
Scale bars = 50µm.

## Slide 17
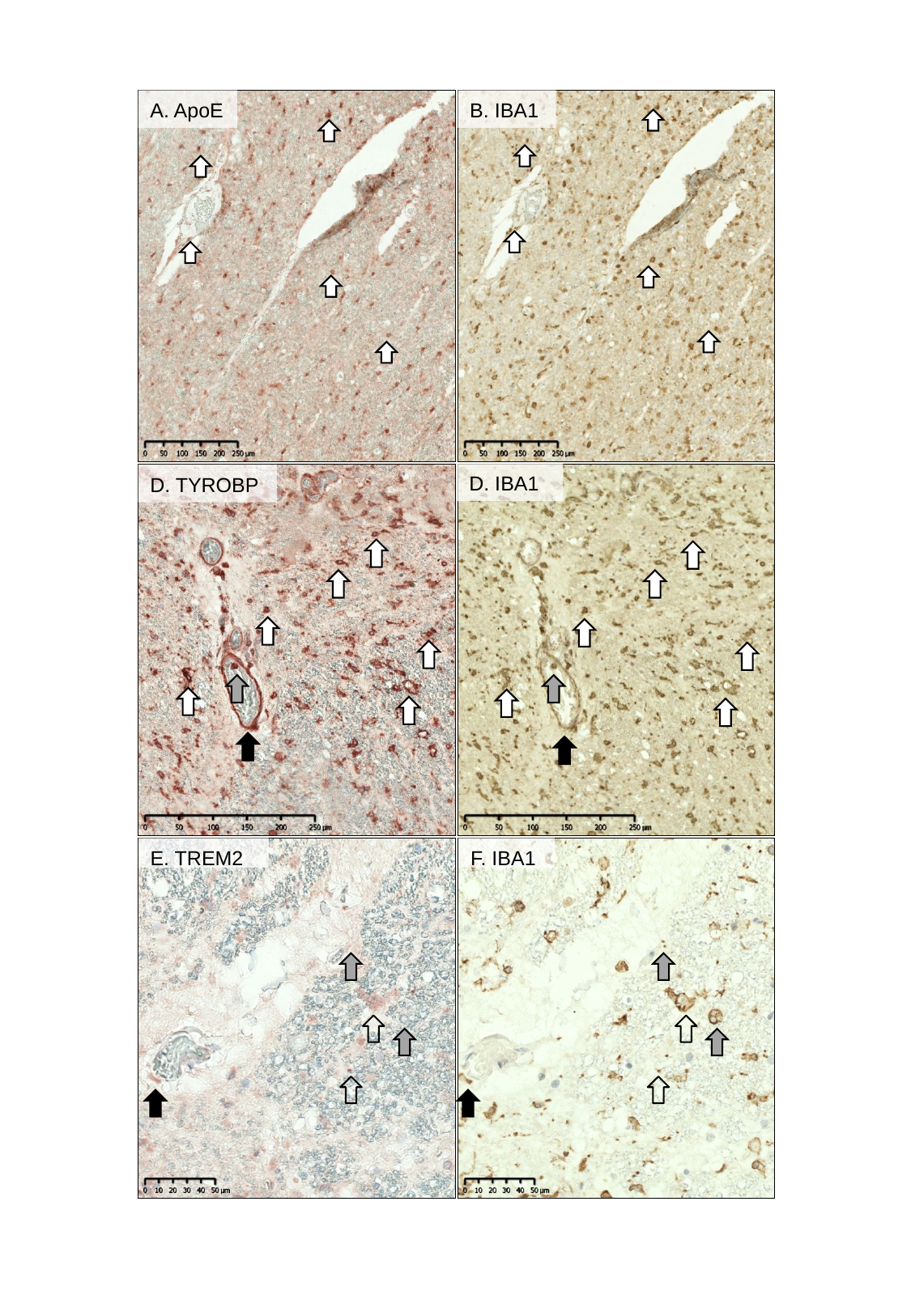

A. ApoE
B. IBA1
D. IBA1
D. TYROBP
E. TREM2
F. IBA1

## Slide 18
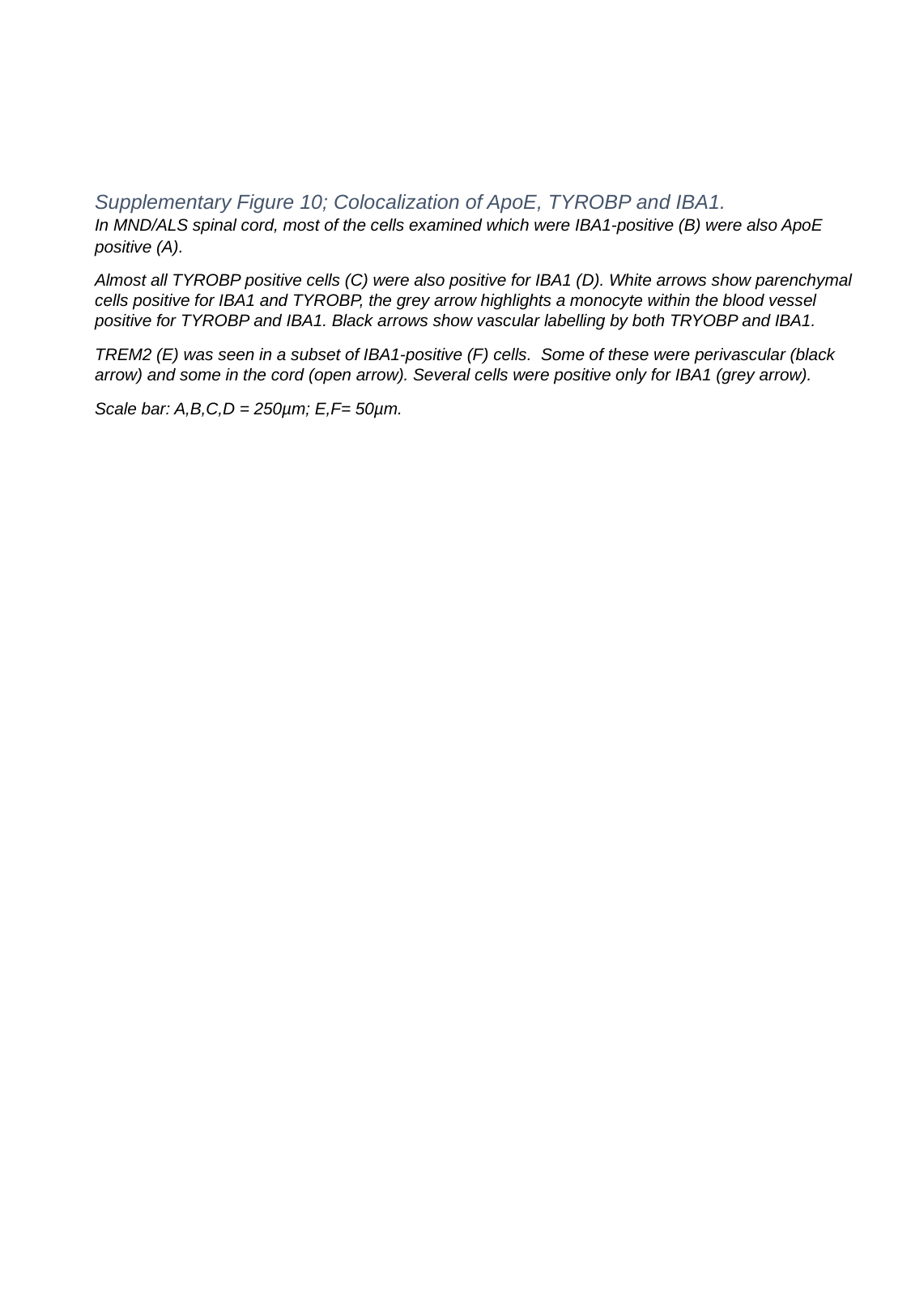

Supplementary Figure 10; Colocalization of ApoE, TYROBP and IBA1.
In MND/ALS spinal cord, most of the cells examined which were IBA1-positive (B) were also ApoE positive (A).
Almost all TYROBP positive cells (C) were also positive for IBA1 (D). White arrows show parenchymal cells positive for IBA1 and TYROBP, the grey arrow highlights a monocyte within the blood vessel positive for TYROBP and IBA1. Black arrows show vascular labelling by both TRYOBP and IBA1.
TREM2 (E) was seen in a subset of IBA1-positive (F) cells. Some of these were perivascular (black arrow) and some in the cord (open arrow). Several cells were positive only for IBA1 (grey arrow).
Scale bar: A,B,C,D = 250µm; E,F= 50µm.

## Slide 19
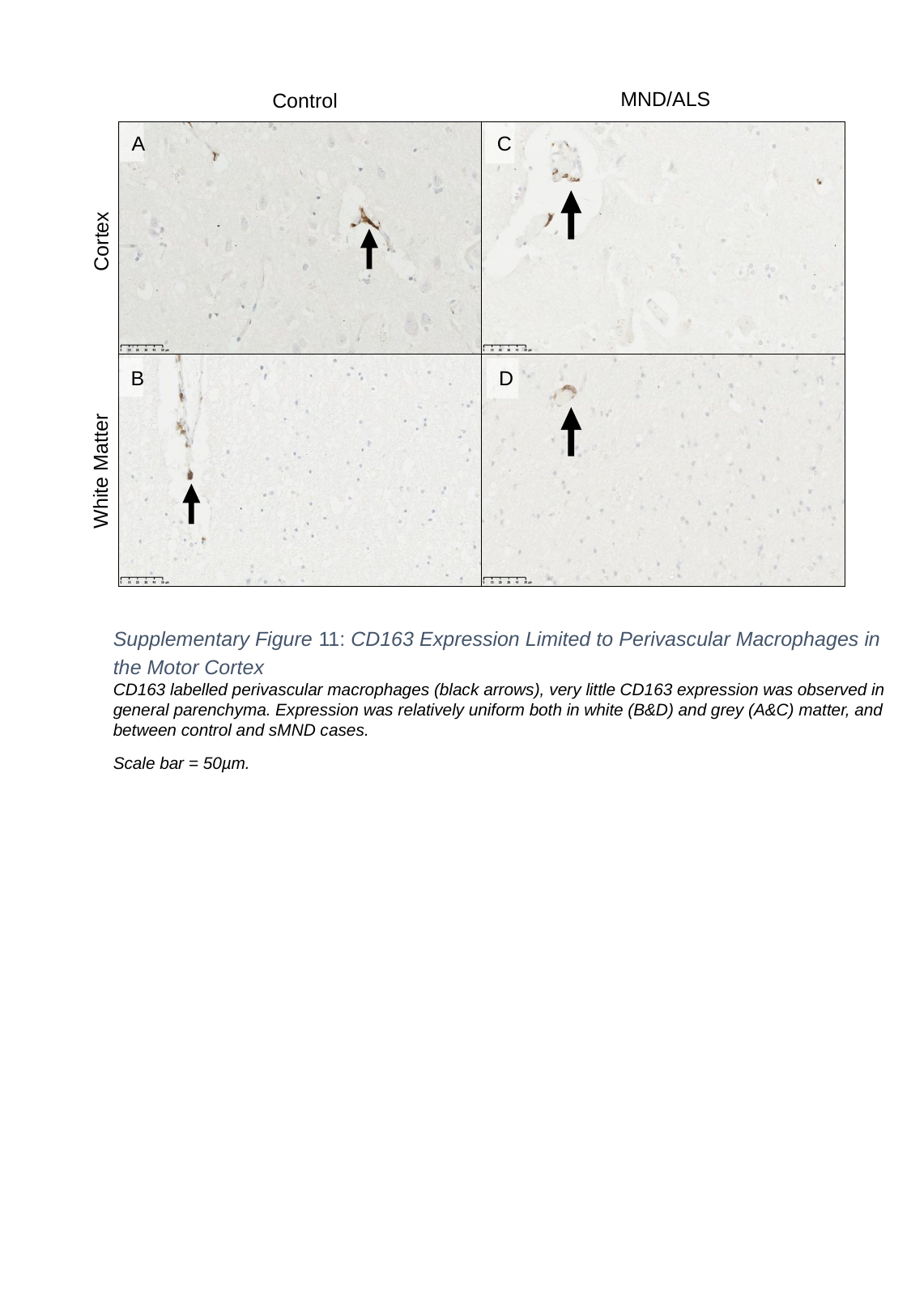

MND/ALS
Control
A
C
Cortex
B
D
White Matter
Supplementary Figure ‎11: CD163 Expression Limited to Perivascular Macrophages in the Motor Cortex
CD163 labelled perivascular macrophages (black arrows), very little CD163 expression was observed in general parenchyma. Expression was relatively uniform both in white (B&D) and grey (A&C) matter, and between control and sMND cases.
Scale bar = 50µm.

## Slide 20
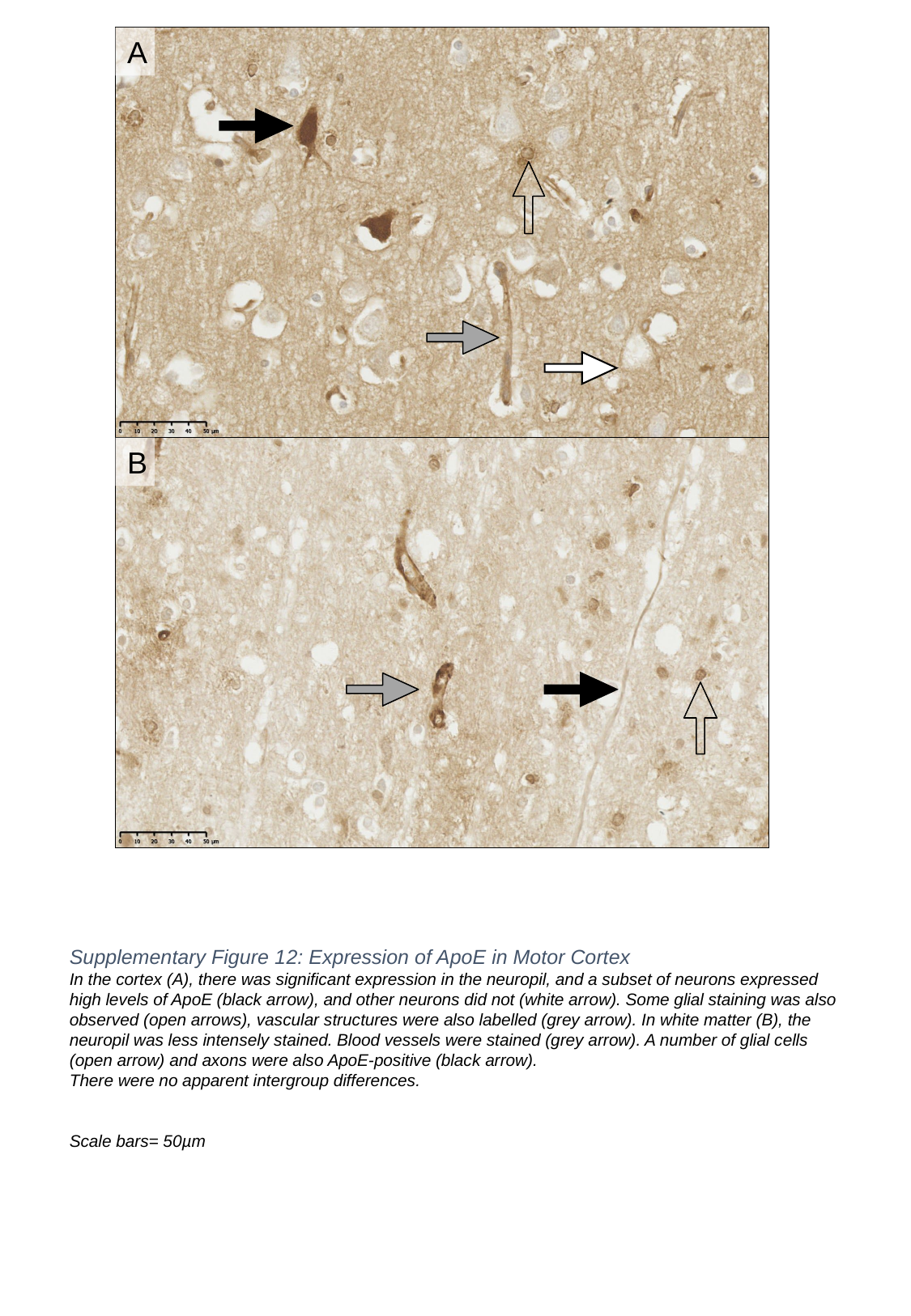

A
B
Supplementary Figure 1‎2: Expression of ApoE in Motor Cortex
In the cortex (A), there was significant expression in the neuropil, and a subset of neurons expressed high levels of ApoE (black arrow), and other neurons did not (white arrow). Some glial staining was also observed (open arrows), vascular structures were also labelled (grey arrow). In white matter (B), the neuropil was less intensely stained. Blood vessels were stained (grey arrow). A number of glial cells (open arrow) and axons were also ApoE-positive (black arrow).
There were no apparent intergroup differences.
Scale bars= 50µm

## Slide 21
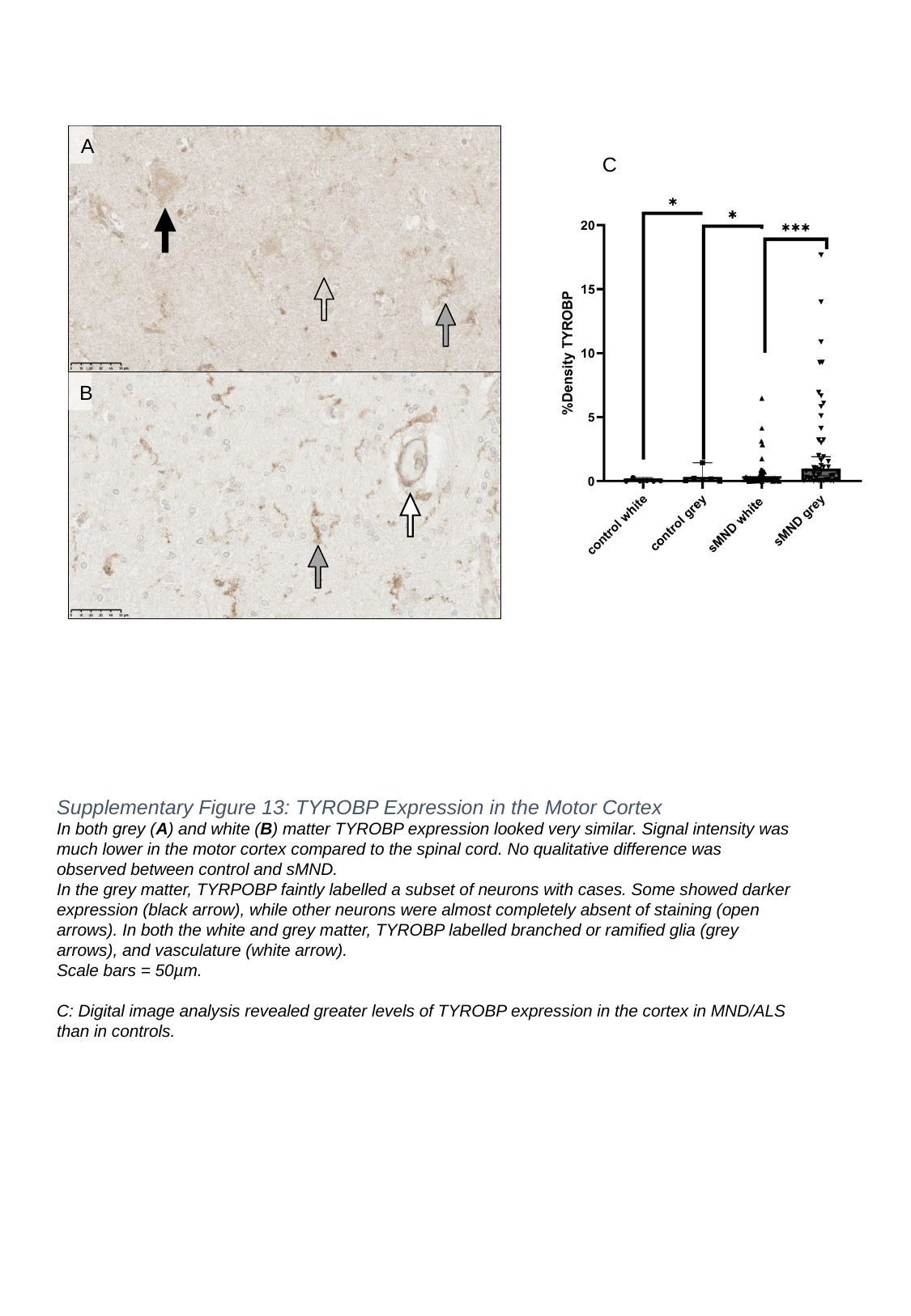

A
C
B
Supplementary Figure 13: TYROBP Expression in the Motor Cortex
In both grey (A) and white (B) matter TYROBP expression looked very similar. Signal intensity was much lower in the motor cortex compared to the spinal cord. No qualitative difference was observed between control and sMND.
In the grey matter, TYRPOBP faintly labelled a subset of neurons with cases. Some showed darker expression (black arrow), while other neurons were almost completely absent of staining (open arrows). In both the white and grey matter, TYROBP labelled branched or ramified glia (grey arrows), and vasculature (white arrow).
Scale bars = 50µm.
C: Digital image analysis revealed greater levels of TYROBP expression in the cortex in MND/ALS than in controls.

## Slide 22
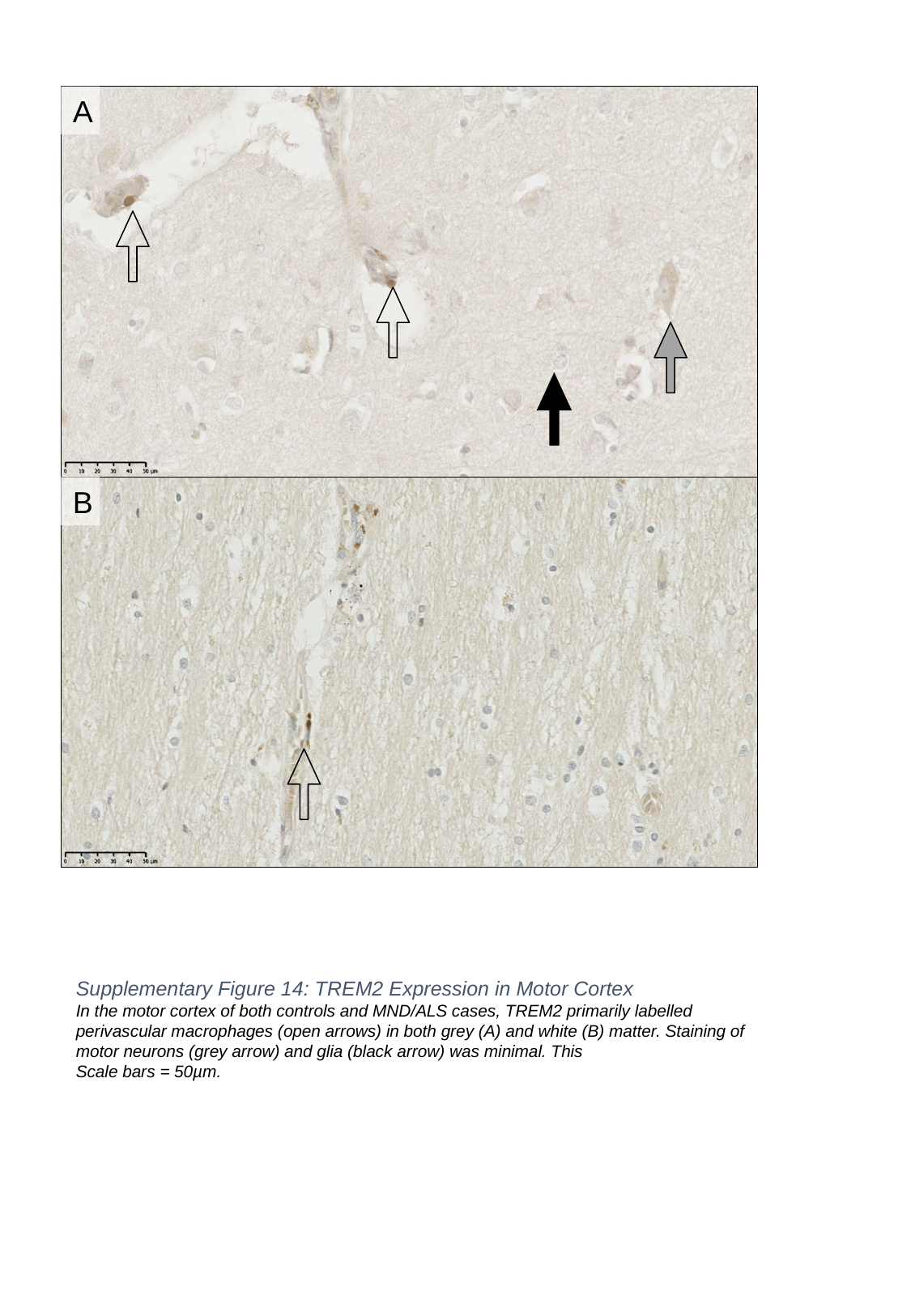

A
B
Supplementary Figure 14: TREM2 Expression in Motor Cortex
In the motor cortex of both controls and MND/ALS cases, TREM2 primarily labelled perivascular macrophages (open arrows) in both grey (A) and white (B) matter. Staining of motor neurons (grey arrow) and glia (black arrow) was minimal. This
Scale bars = 50µm.

## Slide 23
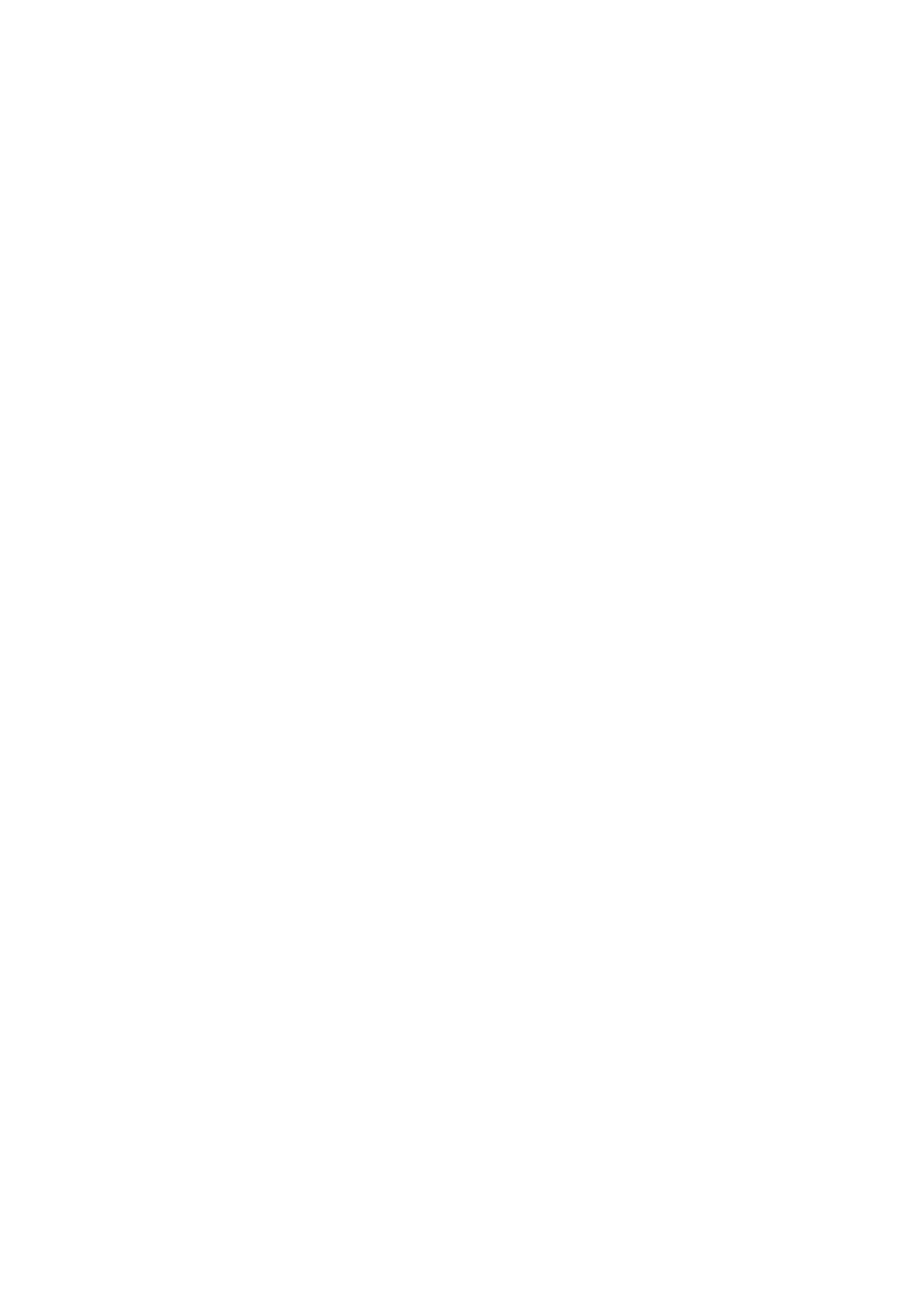

## Slide 24
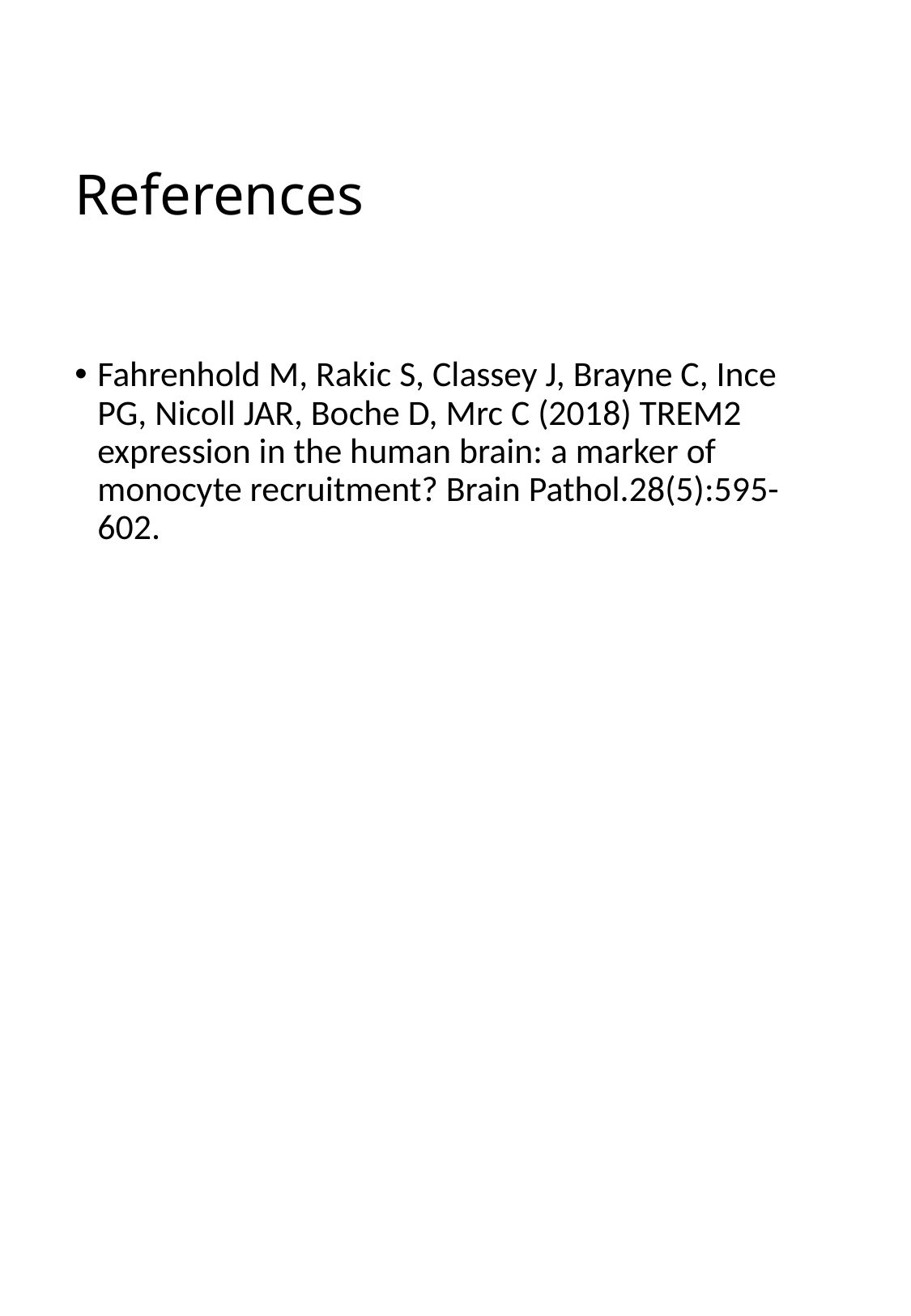

# References
Fahrenhold M, Rakic S, Classey J, Brayne C, Ince PG, Nicoll JAR, Boche D, Mrc C (2018) TREM2 expression in the human brain: a marker of monocyte recruitment? Brain Pathol.28(5):595-602.
